# Supplementary material for: Industry views of the UK Soft Drinks Industry Levy: a thematic analysis of elite interviews with food and drink industry professionals, 2018–2020
Source: BMJ Open. 2023 Aug 9;13(8):e072223. doi: 10.1136/bmjopen-2023-072223 (PMC10414076; doi:10.1136/bmjopen-2023-072223)
Supplement: Supplementary data [file bmjopen-2023-072223supp001.pdf]

Supplementary File 1: Researcher Reflexivity

Braun and Clarke discuss reflexivity as a fundamental characteristic of thematic analysis, involving critical reflection of researcher perspectives, and how these will be integrated within the analysis and interpretation of data [22]. The complete elimination of bias is not something that can be achieved in qualitative research and more importantly should not be an aim. Unlike statistical analysis, the researcher is the tool of analysis. The researcher therefore is an integral part of the analytic process and to conduct qualitative thematic analysis well, they must develop an understanding or how their own perspectives, position and view of reality helps illuminate will influence the analysis [22].

This study aimed to centre participants' words in a descriptive manner to preserve their intention. This approach was also chosen due to reflection by the research team on our positionality as public health academics. We aimed to understand industry perspectives regarding the SDIL; however, it is important to acknowledge that the personal and professional goals of the research team (authors on this paper) as public health researchers are likely to be different from those of people working in the food and drink industry. Therefore, a descriptive approach was selected which prizes participants' words and perspectives over and above researcher interpretations. Whilst our perspectives have still influenced the analysis, as they should in good qualitative practice, we sought to minimise the influence of our biases and negativity towards some of the practices of the food and drink industry, to truly 'listen' to the perspectives of our participants. As a result, a modified version of Braun and Clarke's thematic analysis was used; reflexivity was a priority throughout the analysis in line with the approach however we sought to be less interpretive than their more recent guidance proposes [22].

It is also important to note that, although we have taken a descriptive approach, the results represent participant perspectives. Whilst the researchers work to put aside their biases which may lean towards those more critical of the food and drink industry; statements, findings and themes found do not represent an objective truth, rather the reported perspectives of participants. We urge readers of this work to use their own critical reflection when interpreting and using these findings.
