# Supplementary material for: Industry views of the UK Soft Drinks Industry Levy: a thematic analysis of elite interviews with food and drink industry professionals, 2018–2020
Source: BMJ Open. 2023 Aug 9;13(8):e072223. doi: 10.1136/bmjopen-2023-072223 (PMC10414076; doi:10.1136/bmjopen-2023-072223)
Supplement: Author's manuscript [file bmjopen-2023-072223.draft_revisions.pdf]

BMJ Open

BMJ Open is committed to open peer review. As part of this commitment we make the peer review history of every article we publish publicly available.

When an article is published we post the peer reviewers' comments and the authors' responses online. We also post the versions of the paper that were used during peer review. These are the versions that the peer review comments apply to.

The versions of the paper that follow are the versions that were submitted during the peer review process. They are not the versions of record or the final published versions. They should not be cited or distributed as the published version of this manuscript.

BMJ Open is an open access journal and the full, final, typeset and author-corrected version of record of the manuscript is available on our site with no access controls, subscription charges or pay-per-view fees (http://bmjopen.bmj.com).

If you have any questions on BMJ Open's open peer review process please email info.bmjopen@bmj.com

BMJ Open

Industry views of the UK Soft Drinks Industry Levy: a thematic analysis of elite interviews with food and drink industry professionals, 2018-20

Journal:	*BMJ Open*
Manuscript ID	bmjopen-2023-072223
Article Type:	Original research
Date Submitted by the Author:	01-Feb-2023
Complete List of Authors:	Jones, Catrin; University of Cambridge, MRC Epidemiology Unit<br>Forde, Hannah; University of Cambridge, MRC Epidemiology Unit; University of Oxford, Nuffield Department of Primary Care Health Sciences<br>Penney, Tarra; York University, Global Food System and Policy Research, School of Global Health, Faculty of Health<br>Theis, Dolly; University of Cambridge, MRC Epidemiology Unit<br>Cummins, Steven; London School of Hygiene & Tropical Medicine, Population Health Innovation Lab, Department of Public Health, Environments & Society, Faculty of Public Health and Policy<br>Adams, J; University of Cambridge, MRC Epidemiology Unit<br>Law, Cherry; University of Reading, Department of Agri-Food Economics and Marketing<br>Rutter, Harry; University of Bath Department of Social and Policy Sciences, Social and Policy Sciences<br>Smith, Richard ; University of Exeter, Faculty of Health and Life Sciences<br>White, Martin; University of Cambridge, MRC Epidemiology Unit
Keywords:	Health policy < HEALTH SERVICES ADMINISTRATION & MANAGEMENT, Public health < INFECTIOUS DISEASES, QUALITATIVE RESEARCH

SCHOLARONE™
Manuscripts

I, the Submitting Author has the right to grant and does grant on behalf of all authors of the Work (as defined in the below author licence), an exclusive licence and/or a non-exclusive licence for contributions from authors who are: i) UK Crown employees; ii) where BMJ has agreed a CC-BY licence shall apply, and/or iii) in accordance with the terms applicable for US Federal Government officers or employees acting as part of their official duties; on a worldwide, perpetual, irrevocable, royalty-free basis to BMJ Publishing Group Ltd ("BMJ") its licensees and where the relevant Journal is co-owned by BMJ to the co-owners of the Journal, to publish the Work in this journal and any other BMJ products and to exploit all rights, as set out in our licence.

The Submitting Author accepts and understands that any supply made under these terms is made by BMJ to the Submitting Author unless you are acting as an employee on behalf of your employer or a postgraduate student of an affiliated institution which is paying any applicable article publishing charge ("APC") for Open Access articles. Where the Submitting Author wishes to make the Work available on an Open Access basis (and intends to pay the relevant APC), the terms of reuse of such Open Access shall be governed by a Creative Commons licence – details of these licences and which Creative Commons licence will apply to this Work are set out in our licence referred to above.

Other than as permitted in any relevant BMJ Author's Self Archiving Policies, I confirm this Work has not been accepted for publication elsewhere, is not being considered for publication elsewhere and does not duplicate material already published. I confirm all authors consent to publication of this Work and authorise the granting of this licence.

For peer review only

Industry views of the UK Soft Drinks Industry Levy: a thematic analysis of elite interviews with food and drink industry professionals, 2018-20

Dr Catrin P Jones[a*], Dr Hannah Forde [a,<], Dr Tarra L Penney[b], Dr Dolly Theis [a], Prof Steven Cummins[c], Prof Jean Adams [a], Dr Cherry Law[d], Prof Harry Rutter[e], Prof Rich Smith[f], Prof Martin White[a].

a MRC Epidemiology Unit, School of Clinical Medicine, University of Cambridge, United Kingdom

b Global Food System and Policy Research, School of Global Health, Faculty of Health, York University, Canada

c Population Health Innovation Lab, Department of Public Health, Environments & Society, Faculty of Public Health and Policy, London School of Hygiene & Tropical Medicine, United Kingdom

d Department of Agri-Food Economics and Marketing, University of Reading, Reading, UK

e Department of Social and Policy Sciences, University of Bath, Bath, UK

f Faculty of Health and Life Sciences, University of Exeter, Exeter, UK

For peer review only

< Present address: Nuffield Department of Primary Care Health Sciences, University of Oxford, Oxford, United Kingdom

***Corresponding Author:** Dr Catrin Pedder Jones, MRC Epidemiology Unit, University of Cambridge School of Clinical Medicine, Box 285 Institute of Metabolic Science, Cambridge Biomedical Campus, Cambridge, CB2 0QQ, United Kingdom. Catrin.jones@mrc-epid.cam.ac.uk +44 (0)1223 769 114

Keywords: Soft drinks, Soft drinks industry levy, Food and drink industry, sugar sweetened beverage

Word count: *6799 (without quotes 5192, with reflexivity section in supplementary file 4915)*

Abstract

Background: The UK Soft Drinks Industry Levy (SDIL), implemented in 2018, has been successful in reducing the sugar content, and purchasing, of soft drinks, with limited financial impact on industry. Understanding the views of food and drink industry professionals involved in reacting to the SDIL is important for policymaking. However, their perceptions of the challenges of implementation and strategic responses are unknown.

For peer review only - http://bmjopen.bmj.com/site/about/guidelines.xhtml

Aim: To explore how senior food and drink industry professionals viewed the SDIL.

Methods: We undertook a qualitative descriptive study using elite interviews with 14 senior professionals working in the food and drink industry. Braun and Clarke's approach to thematic analysis was used to analyse the data, taking an inductive exploratory and descriptive approach not informed by prior theory or frameworks.

Results: Five main themes were identified: *(1) A level playing field…for some*; Industry accepted the SDIL as an attempt to create a level playing field but due to the exclusion of milk-based drinks, this was viewed as inadequate. *(2) Complex to implement, but no lasting negatives*; The SDIL was complex, expensive and time consuming to implement, with industry responses dependent on leadership buy-in. "(3) *Why us? – the SDIL unfairly targets the drinks industry*"; soft drinks are an unfair target when other categories also contain high sugar. "(4) *The consumer is king";* Consumers were a key focus of the industry response to this policy. "(5) *The future of the SDIL*"; There appeared to be a wider ripple effect, which primed industry to prepare for future regulation in support of health and environmental sustainability.

For peer review only - http://bmjopen.bmj.com/site/about/guidelines.xhtml

Conclusions: Insights from senior food and drink industry professionals illustrate how sugar sweetened beverage taxes might be successfully implemented and improve understanding of industry responses to taxes and other food and drink policies.

Strengths and Limitations

- This qualitative study explored how senior food and drink industry professionals viewed the SDIL.
- We undertook elite interviews with 14 professionals working in the food and drink industry, who have often been difficult to recruit in other studies.
- Braun and Clarke's thematic analysis taking a descriptive approach was used to analyse the data.
- Elite interviewing methods allow for the building of relationships to elicit meaningful responses from participants.
- Due to recruitment challenges, interviews were carried out over a long period of time, meaning participants experienced different political contexts when data was collected.

For peer review only

For peer review only

For peer review only - http://bmjopen.bmj.com/site/about/guidelines.xhtml

Introduction

Diet-related non-communicable diseases are a major and growing problem, responsible for over 11 million deaths globally each year [1]. Sugar consumption is of particular concern, with the World Health Organization (WHO) recommending member states introduce sugar-sweetened beverage (SSB) taxes (World Health Organization, 2017). The Soft Drinks Industry Levy (SDIL), announced on 16th March 2016 and implemented in the United Kingdom (UK) on 6th April 2018, was designed to incentivise manufacturers of SSBs to reformulate their products [3] via charging a levy on soft drinks produced by companies when they leave the warehouse or when imported into the country [4]. Part of the UK Government's Childhood Obesity: A Plan For Action [5], the SDIL consists of two tiers (for particulars of the tax see box 1). The Government published a second chapter of its childhood obesity plan in 2018, which suggested the SDIL may be extended to milk-based drinks, though this has not yet occurred (Department of Health and Social Care, 2018).

Box 1: Soft Drinks Industry Levy Particulars [3]

Eligible drinks:

- ≥8 g total sugar per 100 mL charged at 24 pence per litre

For peer review only - http://bmjopen.bmj.com/site/about/guidelines.xhtml

- ≥5 g and <8 g total sugar per 100 mL charged at 18 pence per litre

Exemptions:

- Drinks containing more than 75% milk or 1.2% alcohol
- Alcohol replacement drinks
- Powdered drinks
- 100% fruit juices

Manufacturers selling under one million litres of drinks per year

For peer review only

The SDIL was one of the first SSB fiscal interventions explicitly designed to incentivise reformulation [3,6]. This aim was largely achieved, substantially reducing overall SSB sugar content, and inducing a major shift of drinks from the higher levy tier to the lower tier and untaxed bracket between 2016 and 2018 (Scarborough et al., 2020). This shift was reflected in purchases of sugar from SSBs, with a 2.7% or 8g reduction in sugar purchased per household per week in soft drinks one year after implementation [7]. Prior to implementation of the SDIL, the food and drinks industry (hereafter referred to as 'industry') viewed the SDIL as having a potentially negative impact on profits resulting in job losses [8–10]. A negative stock market reaction to the SDIL announcement was observed, but this only lasted

For peer review only - http://bmjopen.bmj.com/site/about/guidelines.xhtml

two days [11]. Similarly, a negative impact on company domestic turnover was observed following the announcement of the SDIL, but this resolved by the time of its implementation [12].

Previous work has investigated industry perspectives of the SDIL expressed through the news media [8,13–16] and the views of industry, civil society and academic participants on how marketing changed in response to the SDIL [17]. A notable gap in the literature, however, is perspectives of the SDIL from the commercial sector, not communicated publicly through news media nor focused solely on marketing responses. Important learning can be obtained by exploring the perspectives of commercial actors involved in responding to regulation. Interviews with senior members of industry can help examine the impact of the SDIL on both the soft drinks industry and wider food and drink industry, an avenue not previously explored. This study therefore aimed to address these knowledge gaps and inform policymaking by exploring the perspectives of senior industry professionals regarding the UK SDIL.

Methods

For peer review only

Study Design

This study adopted a qualitative descriptive design involving elite interviews with senior industry professionals.

Methodological Orientation

This research took an experiential qualitative approach, within a critical realist position. Participant perspectives and perceptions were prized over researcher interpretations, and reality was derived from our participants' words and meaning, rather than a reality constructed through researchers' interpretation of their words [18]. A descriptive approach was used to explore how the SDIL was viewed from the position of our participants.

Research Team

ANONYMISED AUTHORS secured funding for the overall evaluation of the SDIL within which this study formed a part [19]. Interviews were conducted by Postdoctoral Research Associates ANONYMISED AUTHORS. AUTHOR led the design of data collection and AUTHOR led the design of the analysis. AUTHOR & AUTHOR provided guidance on the design of both elements. AUTHOR and AUTHOR recruited and interviewed participants.

For peer review only

AUTHOR led the analysis with support from AUTHOR. AUTHOR conducted secondary coding to support theme generation and interpretation. All authors previously mentioned, as well as AUTHOR and AUTHOR were involved in data analysis and interpretation, as well as drafting this manuscript.

Reflexivity

Braun and Clarke discuss reflexivity as a fundamental characteristic of thematic analysis, involving critical reflection of researcher perspectives, and how these will be integrated within the analysis and interpretation of data [20]. The complete elimination of bias is not something that can be conducted in qualitative research and more importantly should not be an aim. Unlike statistical analysis, the researcher is the tool of analysis. The researcher therefore is an integral part of the analytic process and to conduct qualitative thematic analysis well, they must develop an understanding or how their own perspectives, position and view of reality helps illuminate will influence the analysis [20].

This study aimed to centre participants' words in a descriptive manner to preserve their intention. This approach was also chosen due to reflection by the research team on our

For peer review only

positionality as public health academics. We aimed to understand industry perspectives regarding the SDIL; however, it is important to acknowledge that the personal and professional goals of the research team (authors on this paper) as public health researchers are likely to be different from those of people working in the food and drink industry. Therefore, a descriptive approach was selected which prizes participants' words and perspectives over and above researcher interpretations. Whilst our perspectives have still influenced the analysis, as they should in good qualitative practice, we sought to put aside our biases and negativity towards some of the practices of the food and drink industry, to truly 'listen' to the perspectives of our participants. As a result, a modified version of Braun and Clarke's thematic analysis was used; reflexivity was a priority throughout the analysis in line with the approach.

It is also important to note that, although we have taken a descriptive approach, the results represent participant perspectives. Whilst the researchers work to put aside their biases which may lean towards the critical; statements, findings and themes found do not represent an objective truth, rather the reported perspectives of participants. We urge readers of this work to use their own critical reflection when interpreting and using these findings.

For peer review only

Participants

Senior professionals from the soft drinks, food and other drinks industries were recruited to this study using purposive and snowball sampling. We adopted 'elite interviewing' methods to maximise involvement of senior professionals in positions of influence within their organisation and with high levels of responsibility [21]. This technique provides a series of strategies to support recruitment of difficult to access key participants, and to ensure the validity and reliability of data [22]. The principles of elite interviewing were used to inform recruitment including the maintenance of trust, gauging the tone of the interview, preparing appropriately for the interview, and engaging in dialogue relevant to each informant [23,24].

Individuals were considered eligible to participate based on the following criteria: a) currently or previously held a high-level industry position (at the managerial, director or chief officer level), b) their organisation and their professional role was directly or indirectly impacted by the SDIL and c) they could provide a novel perspective, not previously heard in our interviews, to ensure a range of views. Recruitment typically involved an email introduction by a member of the team or informant contact, although AUTHOR also attended

For peer review only - http://bmjopen.bmj.com/site/about/guidelines.xhtml

industry food events and recruited face to face. Initial contact was followed by an informal telephone conversation with AUTHOR or AUTHOR to discuss the research purpose, team and informant interests and perspectives, ultimately proceeding to full participation via telephone interview.

Data Collection

Telephone interviews were conducted from June 2018 to June 2020. Participant information sheets were sent to potential participants prior to participating in the informal discussion. Informed consent was obtained verbally prior to commencement of the formal telephone interview, which was digitally audio-recorded. Interviews were undertaken using a minimally structured topic guide containing three broad areas of inquiry: a) Can you tell me about your role and organization? b) Can you tell me about your sector as a whole? c) What do you know about the UK Soft Drinks Industry Levy and its impacts? Elite interviewing necessitates informed and adaptive dialogue [23,25], meaning participants could engage in ways most relevant to their specific expertise or experiences within these broad areas. Interviews were transcribed verbatim by a trusted external company, and transcripts were

For peer review only - http://bmjopen.bmj.com/site/about/guidelines.xhtml

checked against the audio files by AUTHOR to identify any inaccuracies. Transcripts were anonymised prior to analysis by removing names of people, organisations, and brands.

Analysis

Analysis commenced once all interviews had been conducted and transcribed. Braun and Clarke's thematic analysis was used, taking an inductive exploratory and descriptive approach not informed by any prior theory or framework [26]. This approach is flexible due to lack of alignment with specific epistemological and ontological stance [27,28]. Six analytic steps were conducted: 1) familiarisation, 2) data coding, 3) initial theme generation, 4) theme development and review, 5) theme refining, defining and naming, 6) writing up.

AUTHOR listened to audio files and read transcripts at least twice to become familiar with them, whilst making notes on initial impressions and patterns (step 1). Following familiarisation, AUTHOR worked systematically through the entire data set and conducted complete coding of all data, in which segments of data were given a label to describe their area of interest. Coding was supported by NVivo software version 12. Semantic codes were derived directly from participants' speech or codes where phrases of speech were brief

For peer review only - http://bmjopen.bmj.com/site/about/guidelines.xhtml

enough to be directly coded (step 2). AUTHOR then sorted these initial codes into concise categories (overarching codes), which clearly described the content of the data (step 3). A reflective diary was kept throughout the coding process by AUTHOR to note reflections on findings and to ensure a data-driven analytic process.

AUTHOR also familiarised herself with the transcripts (step 1) and then examined AUTHOR's coding to ensure the codes were data driven with as little interpretation as possible (step 2). AUTHOR then collated codes that shared a common pattern into themes (step 3). Again, AUTHOR and AUTHOR met to discuss and refine the themes to ensure they were descriptive with minimal interpretation (step 4).

A document containing themes, codes within them, and extensive anonymised quotes was shared with all co-authors in two phases: phase 1 March 2022 and phase 2 October 2022 (step 5). This data clinic aimed to minimise researcher interpretation. A document presented theme descriptions and asked co-authors to answer the following questions for each theme: 1) Is the theme descriptive? 2) Does the theme represent the data accurately? 3) What do you think the theme tells us about the SDIL from the perspective of industry? AUTHORS

For peer review only

For peer review only - http://bmjopen.bmj.com/site/about/guidelines.xhtml

completed the data clinic document in phase 1. Themes were amended based on their reflections and the document updated in October 2022. AUTHORS completed the data clinic form in phase 2. Final themes and the manuscript were written up by AUTHOR and reviewed by all co-authors (step 6).

Patient and Public Involvement Statement

This study is part of the 'Evaluation of the health impacts of the UK Treasury Soft Drinks Industry Levy (SDIL)' funded by NIHR (award no. 16/130/01). Project oversight is provided by an independent study steering committee which contains members of the public.

Results

Fourteen participants were recruited (Table 1). Participants' roles within organisations were diverse; chief officers, directors, and managers with overall responsibility or with specialist responsibilities for finance, strategy, operations, marketing, public relations or nutrition. Interviews ranged in length from 26 to 62 minutes. Six additional participants were approached and took part in informal discussions; three did not participate due to scheduling

For peer review only

issues, and three refused to take part. Five inductively derived, interlinked themes and 15 subthemes were identified (Box 2).

Table 1: Participant details

**Sector Category**	**N**
Drink manufacturers	4
Food and drink manufacturers	3
Supermarkets	3
Industry associations	1
Out-of-home* food and drink manufacturers	1
Out-of-home retailers	1
Advertising consultants	1

*** "The out-of-home sector is generally considered to be any outlet where food or drink is prepared in a way that means it is ready for immediate consumption, on or off the premises"* [29]

For peer review only

For peer review only - http://bmjopen.bmj.com/site/about/guidelines.xhtml

Box 2: Theme and sub-theme summary.

Theme 1: A level playing field…for some

The SDIL created a level playing field

Milk-based drinks increased the complexity in the out-of-home sector

Challenges for supermarkets with large product portfolios

Theme 2: Complex to implement but no lasting negative effects

Complexities in strategic response – price and product are key

Global companies and internal systems

Contradictory government messaging

Leadership buy-in dictates strategic response

Few long-lasting negative effects & SDIL provided opportunities

Theme 3: Why us? – The SDIL unfairly targets the drinks industry

Sugary drinks in isolation were unfair targets for regulation

Distrust of government's motivations to introduce the SDIL

Theme 4: Consumer is king

Consumer response to product changes resulting from the SDIL

Consumer momentum towards healthier products

Theme 5: The future of the SDIL

Extending to milk and fruit-based drinks

Impact on the wider food and drink industry and on other sectors

Proposal to reverse the SDIL

For peer review only

For peer review only - http://bmjopen.bmj.com/site/about/guidelines.xhtml

Theme 1: A level playing field…for some

The SDIL created a level playing field

Industry professionals accepted that the SDIL helped create a level playing field, where no organisation lost out by taking action on health that their competitors did not. Soft drinks manufacturers also discussed that the two years to prepare for the implementation of the SDIL was sufficient and they were happy they could develop an adequate response within that time.

For peer review only - http://bmjopen.bmj.com/site/about/guidelines.xhtml

"… legislation level playing fields is so important and that's why with these big public health initiatives…I'm actually really quite pro government intervention" – Supermarket

"We need to create an even playing field, I mean so therefore we need a government to say this is the rule and it's all going to affect you evenly, now go and do your thing."- Advertising consultant

"I'm not aware of any significant implementation or challenges that our members have encountered, I mean they did have time to adapt, the legislation was published in good time to allow them to understand exactly what they would be required to do" – Trade association

However, participants also stated that a lack of understanding and consultation from government meant a 'true' level playing field for all sectors involved in the sales of sugary drinks had not been not achieved. The lack of consultation by the government with sectors who were not soft drinks manufacturers and the exclusion of milk-based sugary drinks led to this perception.

For peer review only - http://bmjopen.bmj.com/site/about/guidelines.xhtml

"If you're going to do something like this you want to really do it smartly so everybody feels they're 100% equally affected and you don't get this interpretations and this 'my product is in scope, your product is out of scope'…it doesn't create the sense of unilateral 'let's do this', you know, which is what it should be, if that makes sense" – Food and drink manufacturer

"and then even within the levy itself, you know, we saw that milk-based drinks often carried bigger serving sizes and had more total sugar in them than any of our products would. They were excluded from the levy as well which looked like a big shortcoming." Drink manufacturer

Milk-based drinks increased the complexity in the out-of-home sector

Interviewees explained that the government did not think clearly about the technical implications for retailers and out-of-home sector and that it was easier for soft drink manufacturers to respond to the levy than it was for other industry actors. A high level of complexity within the out-of-home sector to manufacture and produce drinks for immediate consumption led to higher implementation costs; specifically, the exclusion of milk-based

For peer review only - http://bmjopen.bmj.com/site/about/guidelines.xhtml

drinks and specification around eligibility of drinks mixed with carbon dioxide, water and ice, and those with and without milk.

"…I don't think they understood the ways of working and the preparation methods in the out-of-home sector…they were looking at the likes of drinks fountains for carbonated soft drinks because… you would have them coming through a bag and box syrup, they would be mixed with ice or carbon dioxide to give the carbonation or either they could be mixed with water and that would capture those drinks in the out-of-home sector, but there was a vagueness to milk-based drinks" – Out-of-home food and drink manufacturer

Thus, the out-of-home sector had to interpret the legislation themselves and apply the SDIL according to their interpretation, as queries to Her Majesty's Revenue and Customs (HMRC – the tax collecting authority in the UK) went unanswered. The out-of-home sector did not perceive two years as enough time to have prepared due to confusion surrounding eligibility. In contrast, soft drinks manufacturers stated they had had time to prepare.

Challenges for supermarkets with large product portfolios

For peer review only - http://bmjopen.bmj.com/site/about/guidelines.xhtml

Supermarkets felt disadvantaged compared to soft drink manufacturers by the complexities of their sector. They highlighted sector-specific challenges to adapting to the SDIL, including that their product portfolio not only contains branded drinks, about which they have to make decisions, but also private label (own brand) drinks. It was described as challenging and time consuming to manage such a large portfolio and make decisions on each product. Retailers also felt that they were disadvantaged as their customers expressed confusion at differing responses by different brands – e.g. 'sugary' drinks reformulated to just below the SDIL threshold but containing both sugar and sweeteners confused customers, with queries directed at retailers rather than drinks manufacturers.

"…what branded suppliers chose to do was their choice…different brands choosing to reformulate, resize or inflate, which I think led to a fair bit of customer confusion as to what the hell was going on. " – Supermarket

"… we tried to make it as clear for customers by putting on all the [shelves] sugar levy applied, so they could very much see…But…when they see a sugar line that's not [included in the SDIL], that's when the questions start coming." – Supermarket

For peer review only - http://bmjopen.bmj.com/site/about/guidelines.xhtml

Theme 2: Complex to implement but no lasting negative effects

Complexities in strategic response – price and product are key

Industry responded to the SDIL by reviewing product portfolios and strategically selecting responses at the individual product level. This portfolio review approach is why responses differed between companies and between products. Research and development (R&D) and consumer testing were costly for industry during this process, and, linking to theme 1, there were increased costs for those companies with larger product portfolios (e.g. supermarkets). For the out-of-home sector, additional complications were noted due to confusion over eligibility of some milk-based drinks.

"…government is very keen to always say "oh just reformulate, it will be easy" but it's not easy. It actually takes a lot of time and investment, which isn't necessarily something that all companies have the resources to do." – Drink manufacturer

Consumer testing was vital during the reformulation and decision-making process and consumer preference dictated the strategy taken. An additional challenge in reformulating

For peer review only - http://bmjopen.bmj.com/site/about/guidelines.xhtml

drinks described by manufacturers was that sugar serves a functional purpose, in the mouthfeel of drinks mixed with ice and to prevent 'brain freeze', as well as to provide sweetness.

"… we invested a significant amount of money then actually in developing lots and lots of different formulations with lower sugar to see and testing them with consumers in Great Britain to see whether those recipes, those new recipes would be acceptable to consumers." – Drink manufacturer

"Because, actually, yes, we could stick sweeteners in everything, but, actually, sugar also has like a functional role" – Out-of-home retailer

Packaging, merchandising and placement were challenges to overcome, particularly for supermarkets. Decisions were made on own brand products but also on how to retail other branded products with different responses to the SDIL (e.g. reformulated drinks, reduced and increased portion sizes, rebranding).

For peer review only - http://bmjopen.bmj.com/site/about/guidelines.xhtml

"…there were a number of products that didn't reformulate but did drop size. So, again, there's just small considerations in that around how you merchandise it, on shelf all our products are measured and then the shelf layout is essentially structured around the size of these products… So what sounds like a relatively simple change, of dropping from 330ml to, I don't know, 250ml, in reality kind of that complexity flows back through the value chain" – Supermarket

Leadership buy-in dictates strategic response

Leadership buy-in to health within a company was discussed as vital in dictating the strategic response to the SDIL. Participants described this buy-in as making the process simpler and a lack of buy-in as a barrier to making timely progress.

"… I think such a review requires strong leadership and … our COO was very clear that we needed to step in and we needed to do, you know, do the responsible, brave thing." -Drink manufacturer

For peer review only

"There are parts of the business that were like "we've always produced sugary drinks and we should continue to produce sugary drinks" and I guess you'll get that from any organisation but having that strong leadership and, you know, complete buy-in from the top team and actually pretty much all the other levels of the organisation, then it's actually quite simple" – Drink manufacturer

Global companies and internal systems

The cost of setting up internal systems to account for and pay the SDIL was expensive, due to the requirement to report to HMRC, regardless of whether or not a company involved in the manufacture or selling of soft drinks was liable to pay the levy. The global nature of many of these companies was an additional challenge. Response strategies appropriate for a UK market may not be transferable to other countries, for example reformulation recipes vary due to differences in consumer palate and storage temperatures/facilities.

"…It's ridiculous that, you know, it's cost us half a million pounds just to tell Treasury that actually we don't need to pay it. I mean it's ludicrous really. It doesn't make any sense." – Drink manufacturer

For peer review only

"We want the system to be able to recognise this product has sugar tax applied to it, this is the rate and this is the finished goods litre in terms of that product that you need to apply that rate to. But, of course, that's for the UK, and then Ireland have a separate system, France have a separate system, Mexico have a separate system. " – Food and drink manufacturer

Contradictory government messaging

There was confusion over the aim of the SDIL and contradictory government messaging; whether manufacturers needed to pass on price increases to change consumer behaviour. Participants indicated that they thought price increases should have been passed on to target individual behaviour change; however, manufacturers stated they had no control over whether this occurred as retailers set the price for consumers.

"…[the] government had slightly mixed messages so it was pretty clear from the Department of Health and PHE [Public Health England] *and, for example, back-benchers like the Health Select Committee… that they expected to see prices passed on … I think the Treasury were trying to say, oh soft drinks manufacturers don't have to pass this on, you know, they could*

For peer review only - http://bmjopen.bmj.com/site/about/guidelines.xhtml

absorb it if they wanted to. Well, apart from the fact that most businesses won't absorb a cost if they can avoid it for obvious reasons, it was the opposite of what the Health Department and others wanted…" – Drink manufacturer

Few long-lasting negative effects & SDIL provided opportunities

Participants acknowledged that the SDIL did achieve its aim in stimulating product reformulation to avoid the levy. Although implementation was complex and costly, as previously illustrated, there were few long-lasting negative effects. Some participants suggested the SDIL provided opportunities. However, participants were sceptical that the SDIL would achieve intended reductions in childhood obesity in the UK.

"we… question why a tax on… on a very small number of sugar containing products that are consumed by either adults or children, why it was thought that that would be a, that policy in isolation would be sufficient to reduce obesity rates." – Drink manufacturer

For peer review only

"… but certainly from our perspective we didn't see volume decline as a result of this, that would have been... So you could argue it's positive fiscal policy from that perspective on it, yeah." – Food and drink manufacturer

"I think some of them would have switched back but we've gained new consumers as well which is, you know, how we, which through sampling and advertising essentially." – Drink manufacturer

Theme 3: Why us? – The SDIL unfairly targets the drinks industry

Sugary drinks in isolation were unfair targets for regulation

Participants felt that the SDIL unfairly targets the soft drinks industry. Participants expressed their frustration that a single food category was targeted when other food categories bear a significant proportion of the responsibility for childhood obesity. They expressed the view that multiple nutrients or calories across many food and drink sectors should be targeted by regulation if the government is serious about reducing childhood obesity, particularly as substitution to other non-regulated food categories could negate the impact of the SDIL on health.

For peer review only

"I think one of the other questions that wasn't answered at the very beginning was why would it be just the soft drink levy, why would you not target cakes and biscuits…that's what we didn't understand at the time." - Food and drink manufacturer

There was consensus among participants that it didn't make sense for the government to target a category that they considered was already reducing sugar faster than other food categories. Although the SDIL had accelerated the reformulation progress for some, this was stated to be the existing direction of travel. Participants expressed the view that the sector had been unfairly penalised, and that sectors which reformulate should be praised rather than targeted by regulation when other unregulated categories have contributed little towards achieving health goals.

"… the soft drinks category was already well embarked on the journey to reformulation…part of the industry's disappointment and frustration about the announcement of the levy was that they were already absolutely going to deliver what the levy has now kind of made them deliver" – Trade association

For peer review only - http://bmjopen.bmj.com/site/about/guidelines.xhtml
For peer review only

Distrust of government's motivations to introduce the SDIL

Participants stated that the SDIL was politically motivated, not an evidence-based policy. Government policies targeting obesity were described as contradictory and not aligned with one another, particularly the proposed ban on advertising of less healthy foods on TV and online [30]. According to participants, the advertising ban does not distinguish between reformulated and non-reformulated products, and acts as a disincentive to spending on reformulation if they cannot recoup their investment through advertising new products.

"So if you can take something from 40g of sugar to 20g of sugar but you'd only advertise on TV is it's 5(g), then why bother, right, and it also means that they can't tell the world, look at this amazing thing we've done, we've reformulated this, you know" – Advertising consultant

Perceived disconnectedness between policies led to distrust in the government and a belief that government obesity policy is poorly planned. Distrust was compounded by some companies appearing to be successful at lobbying the government following the announcement, resulting in changes to the regulations as a result of this lobbying, rather than

For peer review only - http://bmjopen.bmj.com/site/about/guidelines.xhtml

on the basis of health or nutrition, in particular the decision to exclude milk-based drinks. Participants stated this was motivated by some companies being able to gain a competitive advantage, as some milk-based drinks have higher sugar content than soft drinks. Participants also referred to the SDIL as a political tool to distract from other things in the budget in which it was announced. The fact that the proposal to establish the SDIL had been kept secret, and the announcement was a shock to many, led to this view.

"… I think this was a decision taken within the Treasury by quite a small group of people and it was announced during a Budget by a Chancellor who was trying to distract from some other economic figures that he maybe wasn't too pleased about." – Drink manufacturer

"I think the timing was a surprise…Yeah and the way it was done without any form of consultation or pre-announcement." – Drink manufacturer

Theme 4: Consumer is king

Consumer response to product changes resulting from the SDIL

For peer review only

Industry participants discussed throughout all previous themes that meeting the wishes of consumers was the priority when responding to the SDIL. Taste preferences and tolerance of reformulation changes were critical and companies expressed concerns that consumers might dislike reformulated products if they changed dramatically in a short time period. Company responses to the SDIL, as well as health and environmental issues more broadly, were vital to maintaining brand loyalty and company reputation in the eyes of consumers. The media were seen as influential in shaping consumer preferences and company reputation, as some newspapers had used graphics to show the sugar content of drinks and this was considered to have influenced purchasing patterns. A small group of very loyal consumers can cause a backlash publicly, which can be picked up by both the news media and social media.

"… obviously what's critical from our perspective is developing a product that consumers still like the taste of whilst reducing their sugar intake so that we were trying to marry-up those two things." – Drink manufacturer

"I think the overall ethos is that you know we can make foods that little bit healthier, we can produce healthier foods but if they don't taste good the consumer isn't going to consume

For peer review only

them, they're not going to repeat purchase them so we really do believe that bringing the two together is a real winning space and is where consumers' heads are at." – Out-of-home food and drink manufacturer

Consumer momentum towards healthier products

Participants stated consumer purchasing patterns are changing, with consumers increasingly choosing lower sugar products, which may also have driven reformulation prior to the SDIL. The policy acted as a catalyst for increasing consumer demand for sugar reduction and some respondents also highlighted the role of social media in driving these trends. Consumers were also reported by participants as "moving away from" artificial ingredients, which leds to challenges in reformulation using non-nutritive sweeteners. Some participants suggested that consumers were not lost when sugar was reduced in their favourite products, due to consumer preferences moving towards prioritising health. It was important to participants and their organisations that consumers have enough choice and there were concerns that regulation could limit choice from some.

For peer review only

" …consumer research that we did which proved that consumers are moving away from sugary drinks, they still want sweet, great-tasting drinks but they just don't want the sugar and the calories" – Drink manufacturer

"A lot of our consumers like ..., , they don't want to have sweeteners, they don't want to have preservatives" – Drink manufacturer

Theme 5: The future of the SDIL

Participants discussed the potential of expanding the SDIL to fruit and milk-based drinks, the wider threat to other products, reformulation in other categories, changes in other sectors as a result of the SDIL and the possibility of its reversal by government.

Extending to milk and fruit-based drinks

Concerns were expressed over the Chancellor's proposal to extend the SDIL to milk-based and fruit-based drinks at the time of the announcement. Participants stated the nutritional benefits of these meant that natural sugars (fructose and lactose) should not be subject to the same regulation as soft drinks. The vitamin and mineral content of these drinks was also

For peer review only - http://bmjopen.bmj.com/site/about/guidelines.xhtml

discussed as a benefit to children who may not be consuming sufficient fruit, vegetables or calcium from other sources. Reformulation of these drinks was considered particularly challenging, as naturally occurring sugars cannot be removed in the same way as added sugars in soft drinks.

"I don't think politicians think it's done. Obviously we've got the review next year on whether milk-based drinks should be included, and then I think it's 2021 when they'll review the levels as well." – Drink manufacturer

"… Now you have products that are being developed with high levels of sugar in them so that really does need to be addressed but you don't want to go down the route of demonising milk because it is still a great source of nutrition." – Out-of-home food and drink manufacturer

Impact on the wider food and drink industry and in other sectors

A wider threat to other products, particularly those included in the PHE Sugar Reduction Strategy [31] (another element of the Childhood Obesity Plan that encouraged voluntary industry reformulation) was discussed. The SDIL demonstrated that the government was

For peer review only

willing to implement policy to regulate the food industry in a way that has not been done before. Food and drink companies discussed their companies' attempts to reformulate products not included in the SDIL. The SDIL was described as a rallying call for industry to improve the healthfulness of products. It was also perceived to cause a ripple effect not just regarding health but also sustainability, environment, media and promotions.

"Yeah, I think there is a ripple effect. So, I think it can be both positive and negative. I think in terms of positive, I think it can force companies to reformulate and be more innovative in driving the use of other ways of sweetening products" - Food and drink manufacturer

"I think the social justice piece is only going to get louder and I think that's probably an opportunity for the environment and public health " - Supermarket

Proposal to reverse the SDIL

Comments made by Boris Johnson in his leadership campaign to become prime minister (July 2019), suggesting he might consider repealing the SDIL, were not taken well by some

For peer review only - http://bmjopen.bmj.com/site/about/guidelines.xhtml

participants; who indicated that companies had invested heavily in implementing the levy. However, some participants suggested that reversing the SDIL would be well tolerated.

" If it was removed…we'd still be doing what we're doing and we'd still be aiming to keep the sugar low…if it is removed we're not going to change our core brand back, you know, they're staying where they're staying." – Drinks manufacturer

"I think, yeah, the industry would be happy to see the back of it because it's just cumbersome, it's just something, it's just another thing to administer." - Food and drink manufacturer

"I suppose it does feel like a backtrack [reversing the SDIL]. Like we've made all this work and it was at the time quite painful in the sense of it was such a massive change through the supply chain so there was so many things to think about" – Out-of-home food and drink manufacturer

Discussion

For peer review only - http://bmjopen.bmj.com/site/about/guidelines.xhtml

Summary

Senior industry perspectives on the SDIL are described in five main themes. Theme 1: *A level playing field…for some*, Theme 2: *Complex to implement, but no lasting negatives*, Theme 3: *Why us? – the SDIL unfairly targets the drinks industry,* Theme 4: *The consumer is king*, and Theme 5: *The future of the SDIL*. The SDIL appeared to create a level playing field which industry accepted, however, this was perceived as inadequate due to the exclusion of milk-based drinks and targeting only SSBs, giving some a competitive advantage. Implementation of the SDIL was time consuming and complex, leading to high financial investment to prepare for it. Strategic response to the SDIL was dependent on leadership buy-in and particularly governed by potential consumer responses to product changes associated with the policy. The announcement and subsequent implementation of the SDIL caused a ripple effect beyond the soft drinks industry. The wider food and drink industry perceived it as evidence of the government being willing to regulate to help achieve health goals.

Strengths and limitations

Strengths include the use of elite interviewing techniques to build relationships with and solicit meaningful responses from participants. These techniques allowed us to obtain the

For peer review only - http://bmjopen.bmj.com/site/about/guidelines.xhtml

views of senior professionals from commercial organisations who have often been difficult to recruit to other studies [32]. As evident from the challenges described in the out-of-home sector and supermarkets, including respondents outside of manufacturing allowed wider exploration of the systemic impacts of the SDIL. A limitation of this work, however, is that interviews were carried out over a long period of time due to challenges in recruitment. Therefore, not all participants experienced the same political context, such as Boris Johnson's threats to reverse the SDIL in July 2019.

A descriptive approach was taken and emphasised throughout, including through our use of independent double coding and interpretation checked by researchers not directly involved in the initial analysis, to ensure that participant perspectives were represented accurately and neutrally in theme generation and commentary. Adopting a descriptive approach could, however, also be viewed as a limitation of this work. Participants were recruited based on the condition that their responses were anonymous and the researchers were adopting a position of neutrality. Therefore, it would be unethical to pursue more interpretive analyses of these responses. There is, however, overlap between some of the responses provided by participants in this work to the 'typical' responses explored by other researchers as an industry 'playbook' [33]. Although it was not the aim of the work to

For peer review only

explore participant responses in relation to the commercial determinants of health, it is possible that participant responses were censored and did not represent the reality of what occurred behind the scenes in the food and drink industry in relation to the SDIL. Further, although researcher neutrality was expressed to participants during recruitment and data collection, the position of interviewers AUTHOR and AUTHOR as public health academics could also have led to participants censoring their responses.

Relationship to prior knowledge

Our findings indicate that the UK soft drinks industry was reformulating products to lower sugar alternatives several years before the SDIL was introduced. The direction of travel was towards healthier drinks as the primary offer for consumers, with the SDIL accelerating the pace of change. This finding is consistent with previous qualitative and quantitative findings [17,34]. Suggestions by our participants of the possibility of consumers substituting other product categories such as confectionary for SSBs, however, have not been born out in quantitative analyses (Rogers et al., 2022).

Consumer preferences for healthier products, and our finding that industry prioritises these health preferences in their decision making, are likely to have triggered the soft drinks

For peer review only - http://bmjopen.bmj.com/site/about/guidelines.xhtml

industry to reformulate products prior to the announcement of the SDIL. The advocacy (e.g. Jamie Oliver and Action on Sugar) in the early 2010s and government threats to regulate industry [35] may have also increased consumer awareness about the health impact of sugar consumption and had a 'signalling effect' to consumers to reduce their sugar consumption [36].

Participants in our study suggested that the SDIL was adopted by the Government because of the existing popularity of sugar reduction among the public. It is likely that the UK public was aware that SSBs harm health much earlier than the policy announcement, resulting from media activity, such as that related to Jamie Oliver's campaigning [37], PHE's [38] and WHO's reports on sugar[39].

Participants also discussed adopting a portfolio review approach, where each product would be assessed individually, when determining their response to the SDIL, which aligns with previous findings that soft drink companies monitor their internal and external contexts, to determine their products' market position in response to a stimulus such as the SDIL, and then respond with marketing or non-marketing activity to influence the purchasing of soft drinks [17].

For peer review only

For peer review only - http://bmjopen.bmj.com/site/about/guidelines.xhtml

Interpretation and implications for policy and practice

Participants expressed concern that policies introduced to combat obesity and other societal issues should be complementary not contradictory. The proposed ban on TV and online advertising of high fat, salt and sugar (HFSS) products by the UK Government [40] was viewed by industry to be misguided as they stated it may stop them being able to advertise their reformulated products; not just those impacted by the SDIL but products voluntarily reformulated which would still be classified as HFSS. Stakeholder requests for consistency across policy areas was also expressed by interviewees regarding this advertising ban [32]. This indicates that a more consistent approach to determining which products government wants industry to change would help ensure policies do not undermine one another and build trust in government amongst industry.

Interviewees reported that the technical aspects of drink production, particularly in the out-of-home sector, were not adequately accounted for in the design of the SDIL. An unintended consequence of the milk-based drink exclusion, led to some organisations having to interpret the particulars of the SDIL whilst their queries to HMRC went unanswered. Experiences of participants in this work align with findings that UK Government policy is set up poorly for the purposes of adequate monitoring and evaluation (D. R. Z. Theis & White,

For peer review only - http://bmjopen.bmj.com/site/about/guidelines.xhtml

2021). Future policy should engage with the wider food and beverage sector once a policy is certain to be implemented, to design and communicate technicalities in ways that avoid industry having to interpret themselves what is required and provide timely responses to queries surrounding implementation.

Unanswered questions and future research

Respondents indicated that lobbying regarding the exclusion of milk-based drinks from the SDIL had been successful. A further avenue for research would be to understand in more detail the access the food and drink industry had to government during the policy making process, and to explore why some of these attempts to influence policy were successful and others not. While this research focused on the SDIL, it would be interesting to explore industry stakeholder views on health regulation more broadly – particularly to examine whether the sentiment of desiring a level playing field can be generalised to other policy areas.

Conclusion

For peer review only

For peer review only - http://bmjopen.bmj.com/site/about/guidelines.xhtml

This study explored food and drink industry perspectives on the SDIL. We found that industry accepted that legislation was useful in levelling the commercially competitive playing field. However, in practice participants stated that the SDIL had not created a ‘true’ level playing field as little consideration had been given to excluded product categories during policy design. Implementation of the SDIL was seen as challenging and costly and leadership buy-in dictated strategic responses to the SDIL. However, no lasting negative effects of implementing the SDIL were reported. Participants stated that only targeting sugary soft drinks was unfair due to the progress already made in the category compared to others (e.g., confectionary). The impact of the SDIL was felt beyond the soft drinks industry, driving other product sectors to reformulate in anticipation of future regulation. The possibility of repeal of the SDIL was met with a mixed response: some stated it would be welcomed by industry while others suggested that given the significant investment already made to comply with the policy, reversing the SDIL would be unwelcome.

Acknowledgements: For the purpose of Open Access, the author has applied a Creative Commons Attribution (CC BY) licence to any Author Accepted Manuscript version arising.

For peer review only - http://bmjopen.bmj.com/site/about/guidelines.xhtml

Competing Interests: None

Contributorship Statement: Dr. Catrin P Jones: Conceptualization, Methodology, Formal analysis, Investigation, Data Curation, Writing - Original Draft, Project administration**.** Dr. Hannah Forde: Formal analysis, Writing - Review & Editing**.** Dr Tarra L. Penney: Conceptualization, Methodology, Investigation, Data Curation, Writing - Review & Editing, Funding acquisition**.** Dr. Dolly R. Z. Theis: Formal analysis, Writing - Review & Editing Prof. Steven Cummins: Conceptualization, Methodology, Formal analysis, Writing - Review & Editing, Supervision, Funding acquisition. Prof. Jean Adams: Formal analysis, Writing - Review & Editing, Funding acquisition. Dr. Cherry Law: Formal analysis, Writing - Review & Editing. Prof. Harry Rutter: Formal analysis, Writing - Review & Editing, Funding acquisition. Prof. Richard Smith: Formal analysis, Writing - Review & Editing, Funding acquisition. Prof. Martin White: Conceptualization, Methodology, Resources, Supervision, Project administration, Funding acquisition

For peer review only - http://bmjopen.bmj.com/site/about/guidelines.xhtml

Funding: This project was funded by the NIHR Public Health Research programme (Grant Nos. 16/49/01 and 16/130/01). At the time this study was conducted CPJ, MW, ELR, HF, TLP, DT, JA, OA, SA, were also supported in part by: Programme grants to the MRC Epidemiology Unit from the Medical Research Council (grant No. MC_UU_12015/6 and MC_UU_00006/7); and the Centre for Diet and Activity Research (CEDAR), a UKCRC Public Health Research Centre of Excellence – funding from the British Heart Foundation, Cancer Research UK, the Economic and Social Research Council, the Medical Research Council, the National Institute for Health Research, and the Wellcome Trust, under the auspices of the UK Clinical Research Collaboration is gratefully acknowledged. HF received funding for her PhD studentship from the Economic and Social Research Council and Public Health England, and she has received further discretionary funding from the Economic and Social Research Council and Murray Edwards College, Cambridge. The views expressed are those of the authors and not necessarily those of the any of the above-named funders. The funders had no role in study design, data collection and analysis, decision to publish, or preparation of the manuscript.

Data sharing statement: The lead author has full access to the data reported in the manuscript.

For peer review only

Ethics approval statement: The study was approved by the Humanities and Social Science Ethics Committee at the University of Cambridge, UK. No approval ID was provided by this committee.

References

1 Afshin A, Sur PJ, Fay KA, *et al.* Health effects of dietary risks in 195 countries, 1990–2017: a systematic analysis for the Global Burden of Disease Study 2017. *The Lancet* 2019;**393**:1958–72. doi:10.1016/S0140-6736(19)30041-8

2 World Health Organization. Taxes on sugary drinks: Why do it? World Health Organization 2017.

3 HM Revenue & Customs. Soft Drinks Industry Levy. 2016.https://www.gov.uk/government/publications/soft-drinks-industry-levy/soft-drinks-industry-levy#:~:text=from the levy.-,Policy objective,to reduce the sugar content (accessed 16 Jun 2020).

4 HM Revenue and Customs. Soft Drinks Industry Levy statistics background and references. 2022.https://www.gov.uk/government/statistics/soft-drinks-industry-levy-statistics/soft-drinks-industry-levy-statistics-background-and-references (accessed 10 Nov 2022).

5 Cabinet Office, Department of Health and Social Care, HM Treasury, *et al.* Childhood obesity: a plan for action. 2016.https://www.gov.uk/government/publications/childhood-obesity-a-plan-for-action (accessed 8 Apr 2021).

6 Gauke D. Budget 2016. 2016. https://www.gov.uk/government/publications/budget-2016-documents

7 Rogers NT, Cummins S, Forde H, *et al.* Associations between trajectories of obesity prevalence in English primary school children and the UK soft drink industry levy: an

For peer review only

For peer review only - http://bmjopen.bmj.com/site/about/guidelines.xhtml

interrupted time series analysis of surveillance data. *medRxiv* Published Online First: 2022. doi:https://doi.org/10.1101/2022.09.16.22280030

8 Hilton S, Buckton CH, Patterson C, *et al.* Following in the footsteps of tobacco and alcohol? Stakeholder discourse in UK newspaper coverage of the Soft Drinks Industry Levy. *Public Health Nutr* 2019;**22**:2317–28. doi:10.1017/S1368980019000739

9 Economics O. The Economic Impact of the Soft Drinks Levy, Final Report. *London: Oxford Economics* 2016.

10 Penney TL, Adams J, White M. Industry reactions to the UK Soft Drinks Industry Levy: Unpacking the evolving discourse from announcement to implementation. *J Epidemiol Community Health (1978)* 2018;**72**:A43. doi:10.1136/jech-2018-SSMabstracts.88

11 Law C, Cornelsen L, Adams J, *et al.* An analysis of the stock market reaction to the announcements of the UK Soft Drinks Industry Levy. *Econ Hum Biol* 2020;**38**:100834. doi:10.1016/j.ehb.2019.100834

12 Law C, Cornelsen L, Adams J, *et al.* The impact of UK Soft Drinks Industry Levy on manufacturers' domestic turnover. *Econ Hum Biol* 2020;:100866.

13 Penney T, Adams J, White M. LB4 Industry reactions to the UK soft drinks industry levy: unpacking the evolving discourse from announcement to implementation. BMJ 2018. A43.1-A43. doi:10.1136/jech-2018-ssmabstracts.88

14 Hilton S, Buckton CH, Katikireddi SV, *et al.* Who says what about sugar-sweetened beverage tax? Stakeholders' framing of evidence: a newspaper analysis. *The Lancet* 2017;**390**:S44. doi:10.1016/s0140-6736(17)32979-3

15 Buckton CH, Hilton S, Patterson C, *et al.* DID PROPONENTS AND OPPONENTS OF THE SOFT DRINKS INDUSTRY LEVY USE THE NEWS MEDIA TO INFLUENCE THE POLICY DEBATE? A QUALITATIVE DISCOURSE ANALYSIS USING PRACTICAL REASONING. *J Epidemiol Community Health (1978)* 2018;**72**:A1–2. doi:10.1136/jech-2018-SSMabstracts.2

16 Buckton CH, Patterson C, Hyseni L, *et al.* The palatability of sugar-sweetened beverage taxation: A content analysis of newspaper coverage of the UK sugar debate. *PLoS One* 2018;**13**:1–15. doi:10.1371/journal.pone.0207576

17 Forde H, Penney TL, White M, *et al.* Understanding Marketing Responses to a Tax on Sugary Drinks: A Qualitative Interview Study in the United Kingdom, 2019. *Int J Health Policy Manag* Published Online First: 23 February 2022. doi:10.34172/ijhpm.2022.5465

For peer review only

18 Braun V, Clarke V. *Successful Qualitative Research: A Practical Guide for Beginners*. SAGE 2013.

19 White M. Evaluation of the health impacts of the UK Treasury Soft Drinks Industry Levy (SDIL). 2017;:1–46.https://www.journalslibrary.nihr.ac.uk/programmes/phr/1613001/#/

20 Braun V, Clarke V. *Thematic analysis: A practical guide*. Sage 2021.

21 Harvey WS. Methodological Approaches for Interviewing Elites. *Geogr Compass* 2010;**4**:193–205. doi:10.1111/j.1749-8198.2009.00313.x

22 Berry JM, Browne W. Validity and Reliability Issues In Elite Interviewing. *PS Polit Sci Polit* 2003;**35**:679–82.

23 Harvey WS. Strategies for conducting elite interviews. *Qualitative Research* 2011;**11**:431–41. doi:10.1177/1468794111404329

24 Goldstein K. Getting in the Door: Sampling and Completing Elite Interviews. *Political Science & Politics* 2002;**35**:669–72. doi:10.1017/S1049096502001130

25 Stephens N. Collecting data from elites and ultra elites: Telephone and face-to-face interviews with macroeconomists. *Qualitative Research* 2007;**7**:203–16. doi:10.1177/1468794107076020

26 Braun V, Clarke V. Using thematic analysis in psychology. *Qual Res Psychol* 2006;**3**:77–101.

27 Braun V, Clarke V. Can I use TA? Should I use TA? Should I not use TA? Comparing reflexive thematic analysis and other pattern-based qualitative analytic approaches. *Couns Psychother Res* 2020;:37–47. doi:10.1002/capr.12360

28 Braun V, Clarke V. What can "thematic analysis" offer health and wellbeing researchers? *Int J Qual Stud Health Well-being* 2014;**9**. doi:10.3402/qhw.v9.26152

29 Department of Health and Social Care. Calorie labelling in the out of home sector: implementation guidance. 2021.https://www.gov.uk/government/publications/calorie-labelling-in-the-out-of-home-sector/calorie-labelling-in-the-out-of-home-sector-implementation-guidance#:~:text=The%20out%20of%20home%20sector%20is%20generally%20considered%20to%20be,on%20or%20off%20the%20premises (accessed 14 Dec 2022).

30 Department for Digital Culture Media & Sport, Department of Health & Social Care. Introducing a total online advertising restriction for products high in fat, sugar and salt (HFSS). 2021.https://www.gov.uk/government/consultations/total-restriction-of-online-advertising-for-products-high-in-fat-sugar-and-salt-hfss/introducing-a-total-

For peer review only - http://bmjopen.bmj.com/site/about/guidelines.xhtml

online-advertising-restriction-for-products-high-in-fat-sugar-and-salt-hfss (accessed 6 Jan 2023).

31 Office for Health Improvement and Disparities. Sugar, salt and calorie reduction and reformulation. 2017.https://www.gov.uk/government/collections/sugar-reduction (accessed 11 Jan 2023).

32 Forde H, Boyland EJ, Scarborough P, *et al.* Exploring the potential impact of the proposed UK TV and online food advertising regulations: a concept mapping study. *BMJ Open* 2022;**12**. doi:10.1136/bmjopen-2021-060302

33 Petticrew M, Katikireddi SV, Knai C, *et al.* "Nothing can be done until everything is done": The use of complexity arguments by food, beverage, alcohol and gambling industries. *J Epidemiol Community Health (1978)* 2017;**71**:1078–83. doi:10.1136/jech-2017-209710

34 Scarborough P, Adhikari V, Harrington RA, *et al.* Impact of the announcement and implementation of the UK Soft Drinks Industry Levy on sugar content, price, product size and number of available soft drinks in the UK, 2015-19: A controlled interrupted time series analysis. *PLoS Med* 2020;**17**:e1003025.

35 Nieburg O. Kellogg shuns "silver bullet" 30% UK cereals sugar cap. bakeryandsnacks.com. 2013.https://www.bakeryandsnacks.com/Article/2013/01/07/Kellogg-shuns-30-sugar-cap-proposal-for-UK-cereals (accessed 6 Jan 2023).

36 Theis D. *What influences government policymaking? The case of childhood obesity in England.* 2022.

37 Boseley S. Jamie Oliver's Sugar Rush: a crusade to save Britain's health. The Guardian. 2015.

38 Public Health England. Sugar Reduction The evidence for action. 2015. https://www.gov.uk/government/publications/sugar-reduction-from-evidence-into-action (accessed 14 Oct 2022).

39 World Health Organization. Sugars intake for adults and children. 2015. https://www.who.int/publications/i/item/9789241549028 (accessed 14 Oct 2022).

40 Department for Digital CM& S, Department of Health & Social Care. Introducing further advertising restrictions on TV and online for products high in fat, salt or sugar: consultation on secondary legislation. 2022.https://www.gov.uk/government/consultations/introducing-further-advertising-restrictions-on-tv-and-online-for-products-high-in-fat-salt-or-sugar-secondary-

For peer review only

legislation/introducing-further-advertising-restrictions-on-tv-and-online-for-products-high-in-fat-salt-or-sugar-consultation-on-secondary-legislation (accessed 15 Dec 2022).

41 Theis D, White M. Is Obesity Policy in England Fit for Purpose? Analysis of Government Strategies and Policies, 1992–2020. *Milbank Quarterly* 2021;**99**:126–70. doi:10.1111/1468-0009.12498

For peer review only

Industry views of the UK Soft Drinks Industry Levy: a thematic analysis of elite interviews with food and drink industry professionals, 2018-20

Journal:	*BMJ Open*
Manuscript ID	bmjopen-2023-072223.R1
Article Type:	Original research
Date Submitted by the Author:	08-Jun-2023
Complete List of Authors:	Jones, Catrin; University of Cambridge, MRC Epidemiology Unit<br>Forde, Hannah; University of Cambridge, MRC Epidemiology Unit; University of Oxford, Nuffield Department of Primary Care Health Sciences<br>Penney, Tarra; York University, Global Food System and Policy Research, School of Global Health, Faculty of Health<br>Theis, Dolly; University of Cambridge, MRC Epidemiology Unit<br>Cummins, Steven; London School of Hygiene & Tropical Medicine, Population Health Innovation Lab, Department of Public Health, Environments & Society, Faculty of Public Health and Policy<br>Adams, J; University of Cambridge, MRC Epidemiology Unit<br>Law, Cherry; University of Reading, Department of Agri-Food Economics and Marketing<br>Rutter, Harry; University of Bath Department of Social and Policy Sciences, Social and Policy Sciences<br>Smith, Richard ; University of Exeter, Faculty of Health and Life Sciences<br>White, Martin; University of Cambridge, MRC Epidemiology Unit
**Primary Subject Heading**:	Health policy
Secondary Subject Heading:	Qualitative research
Keywords:	Health policy < HEALTH SERVICES ADMINISTRATION & MANAGEMENT, Public health < INFECTIOUS DISEASES, QUALITATIVE RESEARCH

I, the Submitting Author has the right to grant and does grant on behalf of all authors of the Work (as defined in the below author licence), an exclusive licence and/or a non-exclusive licence for contributions from authors who are: i) UK Crown employees; ii) where BMJ has agreed a CC-BY licence shall apply, and/or iii) in accordance with the terms applicable for US Federal Government officers or employees acting as part of their official duties; on a worldwide, perpetual, irrevocable, royalty-free basis to BMJ Publishing Group Ltd ("BMJ") its licensees and where the relevant Journal is co-owned by BMJ to the co-owners of the Journal, to publish the Work in this journal and any other BMJ products and to exploit all rights, as set out in our licence.

The Submitting Author accepts and understands that any supply made under these terms is made by BMJ to the Submitting Author unless you are acting as an employee on behalf of your employer or a postgraduate student of an affiliated institution which is paying any applicable article publishing charge ("APC") for Open Access articles. Where the Submitting Author wishes to make the Work available on an Open Access basis (and intends to pay the relevant APC), the terms of reuse of such Open Access shall be governed by a Creative Commons licence – details of these licences and which Creative Commons licence will apply to this Work are set out in our licence referred to above.

Other than as permitted in any relevant BMJ Author's Self Archiving Policies, I confirm this Work has not been accepted for publication elsewhere, is not being considered for publication elsewhere and does not duplicate material already published. I confirm all authors consent to publication of this Work and authorise the granting of this licence.

For peer review only

Industry views of the UK Soft Drinks Industry Levy: a thematic analysis of elite interviews with food and drink industry professionals, 2018-20

Dr Catrin P Jones[a*], Dr Hannah Forde [a,<], Dr Tarra L Penney[b], Dr Dolly Theis [a], Prof Steven Cummins[c], Prof Jean Adams [a], Dr Cherry Law[d], Prof Harry Rutter[e], Prof Rich Smith[f], Prof Martin White[a].

a MRC Epidemiology Unit, School of Clinical Medicine, University of Cambridge, United Kingdom

b Global Food System and Policy Research, School of Global Health, Faculty of Health, York University, Canada

c Population Health Innovation Lab, Department of Public Health, Environments & Society, Faculty of Public Health and Policy, London School of Hygiene & Tropical Medicine, United Kingdom

d Department of Agri-Food Economics and Marketing, University of Reading, Reading, UK

e Department of Social and Policy Sciences, University of Bath, Bath, UK

f Faculty of Health and Life Sciences, University of Exeter, Exeter, UK

For peer review only - http://bmjopen.bmj.com/site/about/guidelines.xhtml
For peer review only

< Present address: Nuffield Department of Primary Care Health Sciences, University of Oxford, Oxford, United Kingdom

***Corresponding Author:** Dr Catrin Pedder Jones, MRC Epidemiology Unit, University of Cambridge School of Clinical Medicine, Box 285 Institute of Metabolic Science, Cambridge Biomedical Campus, Cambridge, CB2 0QQ, United Kingdom. Catrin.jones@mrc-epid.cam.ac.uk +44 (0)1223 769 114

Keywords: Soft drinks, Soft drinks industry levy, Food and drink industry, sugar sweetened beverage

Word count: *6550*

Abstract

Background: The UK Soft Drinks Industry Levy (SDIL), implemented in 2018, has been successful in reducing the sugar content, and purchasing, of soft drinks, with limited financial impact on industry. Understanding the views of food and drink industry professionals involved in reacting to the SDIL is important for policymaking. However, their perceptions of the challenges of implementation and strategic responses are unknown.

For peer review only - http://bmjopen.bmj.com/site/about/guidelines.xhtml

Aim: To explore how senior food and drink industry professionals viewed the SDIL.

Methods: We undertook a qualitative descriptive study using elite interviews with 14 senior professionals working in the food and drink industry. Braun and Clarke's approach to thematic analysis was used to analyse the data, taking an inductive exploratory and descriptive approach not informed by prior theory or frameworks.

Results: Five main themes were identified: *(1) A level playing field…for some*; Industry accepted the SDIL as an attempt to create a level playing field but due to the exclusion of milk-based drinks, this was viewed as inadequate. *(2) Complex to implement, but no lasting negatives*; The SDIL was complex, expensive and time consuming to implement, with industry responses dependent on leadership buy-in. "(3) *Why us? – the SDIL unfairly targets the drinks industry*"; soft drinks are an unfair target when other categories also contain high sugar. "(4) *The consumer is king";* Consumers were a key focus of the industry response to this policy. "(5) *The future of the SDIL*"; There appeared to be a wider ripple effect, which primed industry to prepare for future regulation in support of health and environmental sustainability.

For peer review only - http://bmjopen.bmj.com/site/about/guidelines.xhtml

For peer review only

Conclusions: Insights from senior food and drink industry professionals illustrate how sugar sweetened beverage taxes might be successfully implemented and improve understanding of industry responses to taxes and other food and drink policies.

Strengths and Limitations

- This qualitative study explored how senior food and drink industry professionals viewed the SDIL.
- We undertook elite interviews with 14 professionals working in the food and drink industry, who have often been difficult to recruit in other studies.
- Braun and Clarke's thematic analysis taking a descriptive approach was used to analyse the data.
- Elite interviewing methods allow for the building of relationships to elicit meaningful responses from participants.
- Due to recruitment challenges, interviews were carried out over a long period of time, meaning participants experienced different political contexts when data was collected.

For peer review only

Introduction

Diet-related non-communicable diseases are a major and growing problem, responsible for over 11 million deaths globally each year [1]. Sugar consumption is of particular concern, with the World Health Organization (WHO) recommending member states introduce sugar-sweetened beverage (SSB) taxes [2]. Reviews suggest that they reduce sales of, increases prices of and encourage reformulation of SSBs [3–5] and over 100 SSB taxes have been implemented worldwide covering 52% of the world's population [6]. SSB taxes have a variety of designs with 87% excise taxes [6]. The WHO recommend that a tiered SSB tax be introduced in companies with high administrative capacity, similar to that which has been introduced in the UK [2]. The Soft Drinks Industry Levy (SDIL) was announced on 16th March 2016 and implemented in the United Kingdom (UK) on 6th April 2018. According to the budget speech by George Osborne, Chancellor of the Exchequer at the time, it was designed to incentivise manufacturers of SSBs to reformulate their products [7] via charging a levy on soft drinks produced by companies when they leave the warehouse or when imported into the country [8]. Integrated in August 2016 as part of the UK Government's Childhood Obesity: A Plan For Action [9], the SDIL consists of two tiers (for particulars of the tax see box 1). A public consultation on the proposals between August - October 2016 set

For peer review only - http://bmjopen.bmj.com/site/about/guidelines.xhtml

out the plans for the tiers and exclusions as described in Box 1. Few changes were made as a result of this consultation and the SDIL was given royal assent on 27th April 2017. The Government published a second chapter of its childhood obesity plan in 2018, which suggested the SDIL may be extended to milk-based drinks, though this has not yet occurred [10].

For peer review only

Box 1: Soft Drinks Industry Levy Particulars [7]

Eligible drinks:

- ≥8 g total sugar per 100 mL charged at 24 pence per litre
- ≥5 g and <8 g total sugar per 100 mL charged at 18 pence per litre

Exemptions:

- Drinks containing more than 75% milk or 1.2% alcohol
- Alcohol substitute drinks
- Powdered drinks

For peer review only - http://bmjopen.bmj.com/site/about/guidelines.xhtml

- 100% fruit juices
- Manufacturers selling under one million litres of drinks per year[11]

The SDIL was one of the first SSB fiscal interventions explicitly designed to incentivise reformulation [7,12,13]. This aim was largely achieved, substantially reducing overall SSB sugar content, and inducing a major shift of drinks from the higher levy tier to the lower tier and untaxed bracket between 2016 and 2018 (Scarborough et al., 2020). Reformulation is reflected in purchases of sugar from SSBs, with [14]. Prior to implementation of the SDIL, the food and drinks industry (hereafter referred to as 'industry') viewed the SDIL as having a potentially negative impact on profits resulting in job losses [15–17]. A negative stock market reaction to the SDIL announcement was observed, but this only lasted two days [18]. Similarly, a negative impact on company domestic turnover was observed following the announcement of the SDIL, but this resolved by the time of its implementation [19].

Critical to the success of the SDIL is the implementation of and reaction to the regulations by the drink industry. Therefore, it is important to understand perspectives of the industry as well as those who work in it regarding the implementation of such taxes. Previous work has

For peer review only - http://bmjopen.bmj.com/site/about/guidelines.xhtml

investigated industry perspectives of the SDIL expressed through the news media [15,20–23] and the views of industry, civil society and academic participants on how marketing changed in response to the SDIL [24]. A notable gap in the literature, however, is perspectives of the SDIL from the commercial sector, not communicated publicly through news media nor focused solely on marketing responses. Important learning can be obtained by exploring the perspectives of commercial actors involved in responding to regulation. Interviews with senior members of industry can help examine the impact of the SDIL on both the soft drinks industry and wider food and drink industry, an avenue not previously explored. This study therefore aimed to address these knowledge gaps and inform policymaking by exploring the perspectives of senior industry professionals regarding the UK SDIL.

Methods

Study Design

This study adopted a qualitative descriptive design involving elite interviews with senior industry professionals.

Methodological Orientation

For peer review only

This research took an experiential qualitative approach, within a critical realist position. Participant perspectives and perceptions were prized over researcher interpretations, and reality was derived from our participants' words and meaning, rather than a reality constructed through researchers' interpretation of their words [25]. A descriptive approach was used to explore how the SDIL was viewed from the position of our participants.

Research Team

Prof Martin White (MW), Prof Steven Cummins (SC), Prof Jean Adams (JA), Prof Rich Smith (RS) and Prof Harry Rutter (HR) secured funding for the overall evaluation of the SDIL within which this study formed a part [26]. Interviews were conducted by Postdoctoral Research Associates Dr. Tarra L Penney (TLP) and Dr. Catrin P Jones (CPJ). TLP led the design of data collection and CPJ led the design of the analysis. MW & SC provided guidance on the design of both elements. TLP and CPJ recruited and interviewed participants. CPJ led the analysis with support from Dr Hannah Forde (HF). HF conducted secondary coding to support theme generation and interpretation. All authors previously mentioned, as well as Dr Dolly Theis (DT) and Dr Cherry Law (CL) were involved in data analysis and interpretation, as well as drafting this manuscript.

For peer review only

For peer review only - http://bmjopen.bmj.com/site/about/guidelines.xhtml

Participants

Senior professionals from the soft drinks, food and other drinks industries were recruited to this study using purposive and snowball sampling. We adopted 'elite interviewing' methods to maximise involvement of senior professionals in positions of influence within their organisation and with high levels of responsibility [27]. This technique provides a series of strategies to support recruitment of difficult to access key participants, and to ensure the validity and reliability of data [28]. The principles of elite interviewing were used to inform recruitment including stronger emphasis on the maintenance of trust, importance of interview tone of the interview, preparing appropriately, and engaging in and tailoring dialogue relevant to each informant, more so than in traditional interviews [29,30].

Individuals were considered eligible to participate based on the following criteria: a) currently or previously held a high-level industry position (at the managerial, director or chief officer level), b) their organisation and their professional role was directly or indirectly impacted by the SDIL and c) they could provide a novel perspective, determined by their job role or the company they work for not previously heard in our interviews, to ensure a range

For peer review only

of views. Recruitment typically involved an email introduction by a member of the team or informant contact, although CPJ also attended industry food events and recruited face to face. Initial contact was followed by an informal telephone conversation with TLP or CPJ to discuss the research purpose, team and informant interests and perspectives, ultimately proceeding to full participation via telephone interview.

Data Collection

Telephone interviews were conducted from June 2018 to June 2020. Participant information sheets were sent to potential participants prior to participating in the informal discussion. Informed consent was obtained verbally prior to commencement of the formal telephone interview, which was digitally audio-recorded. Interviews were undertaken using a minimally structured topic guide containing three broad areas of inquiry: a) Can you tell me about your role and organization? b) Can you tell me about your sector as a whole? c) What do you know about the UK Soft Drinks Industry Levy and its impacts? Elite interviewing necessitates informed and adaptive dialogue [29,31], meaning participants could engage in ways most relevant to their specific expertise or experiences within these broad areas. Interviews were transcribed verbatim by a trusted external company, and transcripts were

For peer review only

checked against the audio files by CPJ to identify any inaccuracies. Transcripts were anonymised prior to analysis by removing names of people, organisations, and brands.

Analysis

Analysis commenced once all interviews had been conducted and transcribed. Braun and Clarke's thematic analysis was used, taking an inductive exploratory and descriptive approach not informed by any prior theory or framework [32]. This approach is flexible due to lack of alignment with specific epistemological and ontological stance [33,34]. Six analytic steps were conducted: 1) familiarisation, 2) data coding, 3) initial theme generation, 4) theme development and review, 5) theme refining, defining and naming, 6) writing up.

CPJ listened to audio files and read transcripts at least twice to become familiar with them, whilst making notes on initial impressions and patterns (step 1). Following familiarisation, CPJ worked systematically through the entire data set and conducted complete coding of all data, in which segments of data were given a label to describe their area of interest. Coding was supported by NVivo software version 12. Semantic codes were derived directly from participants' speech or codes where phrases of speech were brief enough to be directly coded

For peer review only

(step 2). CPJ then sorted these initial codes into concise categories (overarching codes), which clearly described the content of the data (step 3). A reflective diary was kept throughout the coding process by CPJ to note reflections on findings and to ensure a data-driven analytic process.

HF also familiarised themself with the transcripts (step 1) and then examined CPJ's coding to ensure the codes were data driven with as little interpretation as possible (step 2). CPJ then collated codes that shared a common pattern into themes (step 3). Again, CPJ and HF met to discuss and refine the themes to ensure they were descriptive with minimal interpretation (step 4).

A document containing themes, codes within them, and extensive anonymised quotes was shared with all co-authors in two phases: phase 1 March 2022 and phase 2 October 2022 (step 5). This data clinic aimed to minimise researcher interpretation. A document presented theme descriptions and asked co-authors to answer the following questions for each theme: 1) Is the theme descriptive? 2) Does the theme represent the data accurately? 3) What do you think the theme tells us about the SDIL from the perspective of industry? JA, DT, CL

For peer review only - http://bmjopen.bmj.com/site/about/guidelines.xhtml

completed the data clinic document in phase 1. Themes were amended based on their reflections and the document updated in October 2022. SC, MW, HR & RS completed the data clinic form in phase 2. Final themes and the manuscript were written up by CPJ and reviewed by all co-authors (step 6).

Patient and Public Involvement Statement

This study is part of the 'Evaluation of the health impacts of the UK Treasury Soft Drinks Industry Levy (SDIL)' funded by NIHR (award no. 16/130/01). Project oversight is provided by an independent study steering committee (ISSC) which contains members of the public. The ISSC for the overall project met biannually from 2017 – 2023 and were asked to provide advice on methodology as well as interpretation of our findings.

Results

Fourteen participants were recruited (Table 1). Participants' roles within organisations were diverse; chief officers, directors, and managers with overall responsibility or with specialist responsibilities for finance, strategy, operations, marketing, public relations or nutrition. Interviews ranged in length from 26 to 62 minutes. Six additional participants were

For peer review only - http://bmjopen.bmj.com/site/about/guidelines.xhtml

approached and took part in informal discussions; three did not participate due to scheduling issues, and three refused to take part. Five inductively derived, interlinked themes and 15 subthemes were identified (Box 2).

For peer review only

Table 1: Participant details

**Sector Category**	**N**
Drink manufacturers	4
Food and drink manufacturers	3
Supermarkets	3
Industry associations	1
Out-of-home* food and drink manufacturers	1
Out-of-home retailers	1
Advertising consultants	1

*** "The out-of-home sector is generally considered to be any outlet where food or drink is prepared in a way that means it is ready for immediate consumption, on or off the premises"* [35]

Box 2: Theme and sub-theme summary.

For peer review only - http://bmjopen.bmj.com/site/about/guidelines.xhtml

Theme 1: A level playing field…for some

The SDIL created a level playing field

Milk-based drinks increased the complexity in the out-of-home sector

Challenges for supermarkets with large product portfolios

Theme 2: Complex to implement but no lasting negative effects

Complexities in strategic response – price and product are key

Global companies and internal systems

Contradictory government messaging

Leadership buy-in dictates strategic response

Few long-lasting negative effects & SDIL provided opportunities

Theme 3: Why us? – The SDIL unfairly targets the drinks industry

Sugary drinks in isolation were unfair targets for regulation

Distrust of government's motivations to introduce the SDIL

Theme 4: Consumer is king

Consumer response to product changes resulting from the SDIL

Consumer momentum towards healthier products

Theme 5: The future of the SDIL

Extending to milk and fruit-based drinks

Impact on the wider food and drink industry and on other sectors

Proposal to reverse the SDIL

For peer review only

Theme 1: A level playing field…for some

The SDIL created a level playing field

Industry professionals accepted that the SDIL helped create a level playing field, where no organisation lost out by taking action on health that their competitors did not *"… legislation*

For peer review only - http://bmjopen.bmj.com/site/about/guidelines.xhtml

level playing fields is so important and that's why with these big public health initiatives…I'm actually really quite pro government intervention" – Supermarket. Soft drinks manufacturers also discussed that the two years to prepare for the implementation of the SDIL was sufficient and they were happy they could develop an adequate response within that time. *"I'm not aware of any significant implementation or challenges that our members have encountered, I mean they did have time to adapt, the legislation was published in good time to allow them to understand exactly what they would be required to do" – Trade association*

However, participants also stated that a lack of understanding and consultation from government meant a 'true' level playing field for all sectors involved in the sales of sugary drinks had not been not achieved *"…you want to really do it smartly so everybody feels they're 100% equally affected and you don't get this…'my product is in scope, your product is out of scope'…it doesn't create the sense of unilateral 'let's do this'…which is what it should be, if that makes sense" – Food and drink manufacturer.* The lack of consultation by the government with sectors who were not soft drinks manufacturers (for example out of home retailers) and the exclusion of milk-based sugary drinks led to this perception *"…milk-*

For peer review only - http://bmjopen.bmj.com/site/about/guidelines.xhtml

based drinks often carried bigger serving sizes and had more total sugar in them than any of our products would. They were excluded from the levy as well which looked like a big shortcoming." Drink manufacturer.

Milk-based drinks increased the complexity in the out-of-home sector

Interviewees explained that, from their perspective, the government did not think clearly about the technical implications for retailers and out-of-home sector and that it was easier for soft drink manufacturers to respond to the levy than it was for other industry actors *"…I don't think they understood the ways of working and the preparation methods in the out-of-home sector…" – Out-of-home food and drink manufacturer.* A high level of complexity within the out-of-home sector to manufacture and produce drinks for immediate consumption led to higher implementation costs; specifically, the exclusion of milk-based drinks and specification around eligibility of drinks mixed with carbon dioxide, water and ice, and those with and without milk. *"…they were looking at the likes of drinks fountains for carbonated soft drinks because… a bag and box syrup, they would be mixed with ice or carbon dioxide to give the carbonation or either they could be mixed with water and that would capture those drinks in the out-of-home sector, but there was a vagueness to milk-based drinks" – Out-of-*

For peer review only

home food and drink manufacturer. Some queries to Her Majesty's Revenue and Customs (HMRC – the tax collecting authority in the UK) went unanswered, thus, the out-of-home sector had to interpret the legislation themselves and apply the SDIL according to their interpretation. Representatives of the out-of-home sector did not perceive two years as enough time to have prepared due to confusion surrounding eligibility. In contrast, soft drinks manufacturers stated they had had time to prepare.

Challenges for supermarkets with large product portfolios

Supermarkets felt disadvantaged compared to soft drink manufacturers by the complexities of their sector. They highlighted sector-specific challenges to adapting to the SDIL, including that their product portfolio not only contains branded drinks, about which they have to make decisions, but also private label (own brand) drinks *"…what branded suppliers chose to do was their choice…different brands choosing to reformulate, resize or inflate, which I think led to a fair bit of customer confusion as to what the hell was going on. " – Supermarket.* It was described as challenging and time consuming to manage such a large portfolio and make decisions on each product. Particularly as reformulation decisions and portion size reduction reportedly differed between brands yet had to be merchandised together within stores.

For peer review only - http://bmjopen.bmj.com/site/about/guidelines.xhtml

Retailers also felt that they were disadvantaged as their customers expressed confusion at differing responses by different brands – e.g. 'sugary' drinks reformulated to just below the SDIL threshold but containing both sugar and sweeteners confused customers, with queries directed at retailers rather than drinks manufacturers *"… we tried to make it as clear for customers by putting on all the [shelves] sugar levy applied, so they could very much see…But…when they see a sugar line that's not [included in the SDIL], that's when the questions start coming." – Supermarket.*

Theme 2: Complex to implement but no lasting negative effects

Complexities in strategic response – price and product are key

Industry responded to the SDIL by reviewing product portfolios and strategically selecting responses at the individual product level. This portfolio review approach is why responses differed between companies and between products. Research and development (R&D) and consumer testing were costly for industry during this process, and, linking to theme 1, there were increased costs for those companies with larger product portfolios (e.g. supermarkets). For the out-of-home sector, additional complications were noted due to confusion over eligibility of some milk-based drinks *"…government is very keen to always say "oh just*

For peer review only - http://bmjopen.bmj.com/site/about/guidelines.xhtml

reformulate, it will be easy" but it's not easy. It actually takes a lot of time and investment." – Drink manufacturer.

Consumer testing was vital during the reformulation and decision-making process and consumer preference dictated the strategy taken *"… we invested a significant amount of money…in developing lots and lots of different formulations with lower sugar to see and testing them with consumers in Great Britain to see whether those recipes…would be acceptable to consumers." – Drink manufacturer.* An additional challenge in reformulating drinks described by manufacturers was that sugar serves a functional purpose, in the mouthfeel of drinks mixed with ice and to prevent 'brain freeze', as well as to provide sweetness *"Because, actually, yes, we could stick sweeteners in everything, but, actually, sugar also has like a functional role" – Out-of-home retailer.*

Packaging, merchandising and placement were challenges to overcome, particularly for supermarkets. Decisions were made on own brand products but also on how to retail other branded products with different responses to the SDIL (e.g. reformulated drinks, reduced and increased portion sizes, rebranding) *"…there were a number of products that didn't*

For peer review only

reformulate but did drop size. So, again, there's just small considerations in that around how you merchandise it… So what sounds like a relatively simple change, of dropping from 330ml to, I don't know, 250ml, in reality kind of that complexity flows back through the value chain" – Supermarket.

Leadership buy-in dictates strategic response

Leadership buy-in to health, where senior management 'buy-in' to the idea that their company should be making pro-health decisions, was discussed as vital in dictating the strategic response to the SDIL *"… I think such a review requires strong leadership and … our COO was very clear that we needed to step in and we needed to do, you know, do the responsible, brave thing." -Drink manufacturer*. Participants described this buy-in as making the process simpler and a lack of buy-in as a barrier to making timely progress *"… having that strong leadership and, you know, complete buy-in from the top team and actually pretty much all the other levels of the organisation, then it's actually quite simple" – Drink manufacturer.*

Global companies and internal systems

For peer review only - http://bmjopen.bmj.com/site/about/guidelines.xhtml

The cost of setting up internal systems to account for and pay the SDIL was expensive, due to the requirement to report to HMRC, regardless of whether or not a company involved in the manufacture or selling of soft drinks was liable to pay the levy *"…It's ridiculous that, you know, it's cost us half a million pounds just to tell Treasury that actually we don't need to pay it." – Drink manufacturer.* The global nature of many of these companies was an additional challenge. Response strategies appropriate for a UK market may not be transferable to other countries, for example reformulation recipes vary due to differences in consumer palate and storage temperatures/facilities *"…that's [computer system] for the UK, and then Ireland have a separate system, France have a separate system, Mexico have a separate system. " – Food and drink manufacturer.*

Contradictory government messaging

There was confusion over whether manufacturers needed to pass on price increases to change consumer behaviour due to contradictory government messaging over the aim of the SDIL. Participants indicated that they thought price increases should have been passed on to target individual behaviour change; however, manufacturers stated they had no control over whether this occurred as retailers set the price for consumers *"…[the] government had*

For peer review only - http://bmjopen.bmj.com/site/about/guidelines.xhtml

For peer review only

slightly mixed messages so it was pretty clear from the Department of Health and PHE [Public Health England] *… that they expected to see prices passed on … I think the Treasury were trying to say, oh soft drinks manufacturers don't have to pass this on... Well, apart from the fact that most businesses won't absorb a cost if they can avoid it for obvious reasons, it was the opposite of what the Health Department and others wanted…" – Drink manufacturer.*

Few long-lasting negative effects & SDIL provided opportunities

Participants acknowledged that the SDIL did achieve its aim in stimulating product reformulation to avoid the levy. Although implementation was complex and costly, as previously illustrated, there were few long-lasting negative effects. Some participants suggested the SDIL provided opportunities *"I think some of them would have switched back but we've gained new consumers as well which is, you know, how we, which through sampling and advertising essentially." – Drink manufacturer*. However, participants were sceptical that the SDIL would achieve intended reductions in childhood obesity in the UK. *"… why [the SDIL] it was thought that that would be a, that policy in isolation would be sufficient to reduce obesity rates." – Drink manufacturer.*

For peer review only - http://bmjopen.bmj.com/site/about/guidelines.xhtml

Theme 3: Why us? – The SDIL unfairly targets the drinks industry

Sugary drinks in isolation were unfair targets for regulation

Participants felt that the SDIL unfairly targets the soft drinks industry. Participants expressed their frustration that a single food category was targeted when other food categories bear a significant proportion of the responsibility for childhood obesity. They expressed the view that multiple nutrients or calories across many food and drink sectors should be targeted by regulation if the government is serious about reducing childhood obesity, particularly as substitution to other non-regulated food categories could negate the impact of the SDIL on health *"…why would it be just the soft drink levy, why would you not target cakes and biscuits…that's what we didn't understand at the time." - Food and drink manufacturer..*

There was consensus among participants that it did not make sense for the government to target a category that they considered was already reducing sugar faster than other food categories. Although the SDIL had accelerated the reformulation progress for some, this was stated to be already occurring prior to the SDIL announcement. Participants expressed the view that the sector had been unfairly penalised, and that sectors which reformulate should be praised rather than targeted by regulation when other unregulated categories have contributed

For peer review only - http://bmjopen.bmj.com/site/about/guidelines.xhtml

little towards achieving health goals. *"… the soft drinks category was already well embarked on the journey to reformulation…part of the industry's disappointment and frustration about the announcement of the levy was that they were already absolutely going to deliver what the levy has now kind of made them deliver" – Trade association*

Distrust of government's motivations to introduce the SDIL

Participants stated that the SDIL was politically motivated, not an evidence-based policy. Government policies targeting obesity were described as contradictory and not aligned with one another, particularly the proposed ban on advertising of less healthy foods on TV and online [36]. According to participants, the advertising ban does not distinguish between reformulated and non-reformulated products, and acts as a disincentive to spending on reformulation if they cannot recoup their investment through advertising new products. *"So if you can take something from 40g of sugar to 20g of sugar but you'd only advertise on TV is it's 5(g), then why bother, right, and it also means that they can't tell the world, look at this amazing thing we've done, we've reformulated this" – Advertising consultant.*

For peer review only - http://bmjopen.bmj.com/site/about/guidelines.xhtml

Perceived disconnectedness between policies led to distrust in the government and a belief that government obesity policy is poorly planned. Distrust was compounded by some companies appearing to be successful at lobbying the government following the announcement, resulting in changes to the regulations as a result of this lobbying, rather than on the basis of health or nutrition, in particular the decision to exclude milk-based drinks. Participants stated this was motivated by some companies being able to gain a competitive advantage, as some milk-based drinks have higher sugar content than soft drinks. Participants also referred to the SDIL as a political tool to distract from other things in the budget in which it was announced *"… I think this was a decision taken within the Treasury by quite a small group of people and it was announced during a Budget by a Chancellor who was trying to distract from some other economic figures that he maybe wasn't too pleased about." – Drink manufacturer*. The fact that the proposal to establish the SDIL had been kept secret, and the announcement was a shock to many, led to this view. *"I think the timing was a surprise... Yeah and the way it was done without any form of consultation or pre-announcement." – Drink manufacturer.*

Theme 4: Consumer is king

For peer review only - http://bmjopen.bmj.com/site/about/guidelines.xhtml

Consumer response to product changes resulting from the SDIL

Industry participants discussed throughout all previous themes that meeting the wishes of consumers was the priority when responding to the SDIL. Taste preferences and tolerance of reformulation changes were critical and companies expressed concerns that consumers might dislike reformulated products if they changed dramatically in a short time period *"… obviously what's critical from our perspective is developing a product that consumers still like the taste of whilst reducing their sugar intake so that we were trying to marry-up those two things." – Drink manufacturer.* Company responses to the SDIL, as well as health and environmental issues more broadly, were vital to maintaining brand loyalty and company reputation in the eyes of consumers. The media were seen as influential in shaping consumer preferences and company reputation, as some newspapers had used graphics to show the sugar content of drinks and this was considered to have influenced purchasing patterns. According to informants a small group of very loyal consumers can cause a backlash publicly, which can be picked up by both the news media and social media.

Consumer momentum towards healthier products

For peer review only - http://bmjopen.bmj.com/site/about/guidelines.xhtml

Participants stated consumer purchasing patterns are changing, with consumers increasingly choosing lower sugar products, which may also have driven reformulation prior to the SDIL. The policy acted as a catalyst for increasing consumer demand for sugar reduction and some respondents also highlighted the role of social media in driving these trends. Consumers were also reported by participants as “moving away from" artificial ingredients, which leds to challenges in reformulation using non-nutritive sweeteners *"A lot of our consumers like ..., , they don’t want to have sweeteners, they don’t want to have preservatives" – Drink manufacturer*. Some participants suggested that consumers were not lost when sugar was reduced in their favourite products, due to consumer preferences moving towards prioritising health. It was important to participants and their organisations that consumers have enough choice and there were concerns that regulation could limit choice from some.

Theme 5: The future of the SDIL

Participants discussed the potential of expanding the SDIL to fruit and milk-based drinks, the wider threat to other products, reformulation in other categories, changes in other sectors as a result of the SDIL and the possibility of its reversal by government.

For peer review only - http://bmjopen.bmj.com/site/about/guidelines.xhtml

Extending to milk and fruit-based drinks

Concerns were expressed over the Chancellor's proposal to extend the SDIL to milk-based and fruit-based drinks at the time of the announcement *"I don't think politicians think it's done. Obviously we've got the review next year on whether milk-based drinks should be included, and then I think it's 2021 when they'll review the levels as well." – Drink manufacturer*. Participants stated the nutritional benefits of these meant that natural sugars (fructose and lactose) should not be subject to the same regulation as soft drinks. The vitamin and mineral content of these drinks was also discussed as a benefit to children who may not be consuming sufficient fruit, vegetables or calcium from other sources *"… Now you have products that are being developed with high levels of sugar in them so that really does need to be addressed but you don't want to go down the route of demonising milk because it is still a great source of nutrition." – Out-of-home food and drink manufacturer*. Reformulation of these drinks was considered particularly challenging, as naturally occurring sugars cannot be removed in the same way as added sugars in soft drinks.

Impact on the wider food and drink industry and in other sectors

For peer review only

For peer review only - http://bmjopen.bmj.com/site/about/guidelines.xhtml

A wider threat to other products, particularly those included in the PHE Sugar Reduction Strategy [37] (another element of the Childhood Obesity Plan that encouraged voluntary industry reformulation) was discussed. The SDIL demonstrated that the government was willing to implement policy to regulate the food industry in a way that has not been done before. Food and drink companies discussed their companies' attempts to reformulate products not included in the SDIL. The SDIL was described as a rallying call for industry to improve the healthfulness of products. It was also perceived to cause a ripple effect not just regarding health but also sustainability, environment, media and promotions. *"Yeah, I think there is a ripple effect. So, I think it can be both positive and negative. I think in terms of positive, I think it can force companies to reformulate and be more innovative in driving the use of other ways of sweetening products" - Food and drink manufacturer*

Proposal to reverse the SDIL

Comments made by Boris Johnson in his leadership campaign to become prime minister (July 2019), suggested he might consider repealing the SDIL [38]. These were not taken well by some participants; who indicated that companies had invested heavily in implementing the levy *"I suppose it does feel like a backtrack [reversing the SDIL]. Like we've made all this*

For peer review only

work and it was at the time quite painful in the sense of it was such a massive change through the supply chain so there was so many things to think about" – Out-of-home food and drink manufacturer. However, some participants suggested that reversing the SDIL would be well tolerated. *"I think, yeah, the industry would be happy to see the back of it because it's just cumbersome, it's just something, it's just another thing to administer." - Food and drink manufacturer.*

Discussion

Summary

Senior industry perspectives on the SDIL are described in five main themes. Theme 1: *A level playing field…for some*, Theme 2: *Complex to implement, but no lasting negatives*, Theme 3: *Why us? – the SDIL unfairly targets the drinks industry,* Theme 4: *The consumer is king*, and Theme 5: *The future of the SDIL.* The SDIL appeared to create a level playing field which industry accepted, however, this was perceived as inadequate due to the exclusion of milk-based drinks and targeting only SSBs, giving some a competitive advantage. Implementation of the SDIL was time consuming and complex, leading to high financial investment to prepare for it. Strategic response to the SDIL was dependent on leadership buy-

For peer review only - http://bmjopen.bmj.com/site/about/guidelines.xhtml

in and particularly governed by potential consumer responses to product changes associated with the policy. The announcement and subsequent implementation of the SDIL caused a ripple effect beyond the soft drinks industry. The wider food and drink industry perceived it as evidence of the government being willing to regulate to help achieve health goals.

Strengths and limitations

The use of elite interviewing techniques to build relationships with and solicit meaningful responses from participants is a strength of this work. These techniques allowed us to obtain the views of senior professionals from commercial organisations who have often been difficult to recruit to other studies [39]. As evident from the challenges described in the out-of-home sector and supermarkets, including respondents outside of manufacturing allowed wider exploration of the systemic impacts of the SDIL. A limitation of this work, however, is that interviews were carried out over a long period of time due to challenges in recruitment. Therefore, not all participants experienced the same political context, such as Boris Johnson's threats to reverse the SDIL in July 2019. Initial plans were for longitudinal data collection repeated across the time period of the study. Had all participants been interviewed closer to the implementation of the SDIL in 2018, then repeated in 2020, perspectives on the political

For peer review only

events occurring would have been captured from all participants. Unfortunately, challenges to recruitment and access to elite participants led to the abandonment of this plan. Although researcher neutrality was expressed to participants the position of interviewers as public health academics could have led to these recruitment challenges.

The positionality of the researchers may also have led to censoring of responses by some participants. Whilst we sought to descriptively represent industry perspectives, as well as acknowledge our own biases that are typically pro-health policy, it is important to acknowledge that the food and drink industry will have their own biases against health policy that is detrimental to their business survival, as evidenced in previous work [40,41]. Although it was not the aim of the work to explore participant responses in relation to the commercial determinants of health, it is possible that participant responses did not represent the reality of what occurred behind the scenes in the food and drink industry in relation to the SDIL. Overlap between some of the responses provided in this work and the 'typical' responses explored by other researchers as an industry 'playbook' [42] may support this assertion.

Interpretation and implications

For peer review only

Interviewees reported that the technical aspects of drink production, particularly in the out-of-home sector, were not adequately accounted for in the design of the SDIL. An unintended consequence of the milk-based drink exclusion, led to some organisations having to interpret the particulars of the SDIL whilst their queries to HMRC went unanswered. Experiences of participants in this work align with findings that UK Government policy is set up poorly for the purposes of adequate monitoring and evaluation [43]. Future policy should engage with the wider food and beverage sector once a policy is certain to be implemented, to design and communicate technicalities in ways that avoid industry having to interpret themselves what is required and provide timely responses to queries surrounding implementation. Further, respondents indicated that lobbying against the inclusion of milk-based sugar sweetened beverages in the SDIL resulted in this exclusion. Alongside policy engagement in the technicalities of production, an avenue for future research would be to understand in more detail the policy process surrounding the SDIL, particularly the influence of the food and drink industry on the policy particulars.

Reviewing their product portfolio was also discussed, where assessments of the product mix as a whole and by individual product were conducted when determining the response to the

For peer review only - http://bmjopen.bmj.com/site/about/guidelines.xhtml

SDIL. This aligns with previous findings that soft drink companies monitor their internal and external contexts to determine their products' market position in response to a stimulus such as the SDIL, and then respond with marketing or non-marketing activity to influence the purchasing of soft drinks [44]. A crucial external contextual component to response in our findings appears to be consumer response and preferences towards each product, as well as health as a whole.

The UK soft drinks industry was reformulating products to lower sugar alternatives several years before the SDIL was introduced [44,45]. Perspectives expressed by participants align with this and suggest that there is a shift towards healthier drinks as the primary offer for consumers, with the SDIL accelerating the pace of this change. Consumer preferences for healthier products, and our finding that industry prioritises these health preferences in their decision making, are likely to have triggered the soft drinks industry to reformulate products prior to the announcement of the SDIL. The advocacy (e.g. Jamie Oliver and Action on Sugar) in the early 2010s [46–48]and government threats to regulate industry [49] may have also increased consumer awareness about the health impact of sugar consumption and had a 'signalling effect' to consumers to reduce their sugar consumption [50]. Participants in our

For peer review only - http://bmjopen.bmj.com/site/about/guidelines.xhtml

study suggested that the SDIL was adopted by the Government because of the existing popularity of sugar reduction among the public. It is likely that the UK public was aware that SSBs harm health much earlier than the policy announcement, resulting from media activity, such as that related to Jamie Oliver's campaigning [46] PHE's [47]and WHO's reports on sugar [48]. Therefore, the importance of public momentum towards health could be regarded as a trigger for industry action independently from encouraging government action via policy.

Finally, participants expressed concern that policies introduced to combat obesity and other societal issues should be complementary not contradictory. The proposed ban on TV and online advertising of high fat, salt and sugar (HFSS) products by the UK Government [36] was viewed by industry to be misguided as they stated it may stop them being able to advertise their reformulated products; not just those impacted by the SDIL but products voluntarily reformulated which would still be classified as HFSS. Stakeholder requests for consistency across policy areas was also expressed by interviewees regarding this advertising ban [51]. This indicates that a more consistent approach to determining which products government wants industry to change would help ensure policies do not undermine one another and build trust in government amongst industry.

For peer review only - http://bmjopen.bmj.com/site/about/guidelines.xhtml

Conclusion

This study explored food and drink industry perspectives on the SDIL. We found that industry accepted that legislation was useful in levelling the commercially competitive playing field. However, in practice participants stated that the SDIL had not created a 'true' level playing field as little consideration had been given to excluded product categories during policy design. Technical aspects of implementation were not adequately included and led to complexity for out of home retailers. Legislation on SSBs needs to take account of all industry sectors it affects, including out of home retail, as well as the manufacturing sector. Participants stated that only targeting sugary soft drinks was unfair due to the progress already made in the category compared to others (e.g., confectionary). The critical role of consumers in creating momentum towards sugar reduction in SSBs prior to the SDIL announcement, as well as dictating response to the SDIL was discussed. It is hypothesised that pro-health public views could be a useful lever in encouraging positive industry action independently of food and drink regulation. The impact of the SDIL was felt beyond the soft drinks industry, driving other product sectors to reformulate in anticipation of future regulation.

For peer review only - http://bmjopen.bmj.com/site/about/guidelines.xhtml

Acknowledgements: For the purpose of Open Access, the author has applied a Creative Commons Attribution (CC BY) licence to any Author Accepted Manuscript version arising.

Competing Interests: None

Contributorship Statement: Dr. Catrin P Jones: Conceptualization, Methodology, Formal analysis, Investigation, Data Curation, Writing - Original Draft, Project administration. Dr. Hannah Forde: Formal analysis, Writing - Review & Editing. Dr Tarra L. Penney: Conceptualization, Methodology, Investigation, Data Curation, Writing - Review & Editing, Funding acquisition. Dr. Dolly R. Z. Theis: Formal analysis, Writing - Review & Editing Prof. Steven Cummins: Conceptualization, Methodology, Formal analysis, Writing - Review & Editing, Supervision, Funding acquisition. Prof. Jean Adams: Formal analysis, Writing - Review & Editing, Funding acquisition. Dr. Cherry Law: Formal analysis, Writing - Review & Editing. Prof. Harry Rutter: Formal analysis, Writing - Review & Editing, Funding acquisition. Prof. Richard Smith: Formal analysis, Writing - Review & Editing, Funding

For peer review only

acquisition. Prof. Martin White: Conceptualization, Methodology, Resources, Supervision, Project administration, Funding acquisition, Formal analysis, Writing - Review & Editing.

Funding: This project was funded by the NIHR Public Health Research programme (Grant Nos. 16/49/01 and 16/130/01). At the time this study was conducted CPJ, MW, ELR, HF, TLP, DT, JA, OA, SA, were also supported in part by: Programme grants to the MRC Epidemiology Unit from the Medical Research Council (grant No. MC_UU_12015/6 and MC_UU_00006/7); and the Centre for Diet and Activity Research (CEDAR), a UKCRC Public Health Research Centre of Excellence – funding from the British Heart Foundation, Cancer Research UK, the Economic and Social Research Council, the Medical Research Council, the National Institute for Health Research, and the Wellcome Trust, under the auspices of the UK Clinical Research Collaboration is gratefully acknowledged. HF received funding for her PhD studentship from the Economic and Social Research Council and Public Health England, and she has received further discretionary funding from the Economic and Social Research Council and Murray Edwards College, Cambridge. The views expressed are those of the authors and not necessarily those of the any of the above-named funders. The

For peer review only

funders had no role in study design, data collection and analysis, decision to publish, or preparation of the manuscript.

Data sharing statement: The lead author has full access to the data reported in the manuscript.

Ethics approval statement: The study was approved by the Humanities and Social Science Ethics Committee at the University of Cambridge, UK. No approval ID was provided by this committee.

For peer review only

References

1 Afshin A, Sur PJ, Fay KA, *et al.* Health effects of dietary risks in 195 countries, 1990–2017: a systematic analysis for the Global Burden of Disease Study 2017. *The Lancet* 2019;**393**:1958–72. doi:10.1016/S0140-6736(19)30041-8

2 World Health Organization. Taxes on sugary drinks: Why do it? World Health Organization 2017.

3 Andreyeva T, Marple K, Marinello S, *et al.* Outcomes following taxation of sugar-sweetened beverages: a systematic review and meta-analysis. *JAMA Netw Open* 2022;**5**:e2215276–e2215276.

4 Teng AM, Jones AC, Mizdrak A, *et al.* Impact of sugar-sweetened beverage taxes on purchases and dietary intake: systematic review and meta-analysis. *Obesity Reviews* 2019;**20**:1187–204.

5 Cawley J, Frisvold D. Taxes on sugar-sweetened beverages: Political economy, and effects on prices, purchases, and consumption. *Food Policy* 2023;**117**:102441.

For peer review only - http://bmjopen.bmj.com/site/about/guidelines.xhtml

6 The World Bank. Global SSB Tax Database. https://ssbtax.worldbank.org/ (accessed 25 May 2023).

7 HM Revenue & Customs. Soft Drinks Industry Levy. 2016.https://www.gov.uk/government/publications/soft-drinks-industry-levy/soft-drinks-industry-levy#:~:text=from the levy.-,Policy objective,to reduce the sugar content (accessed 16 Jun 2020).

8 HM Revenue and Customs. Soft Drinks Industry Levy statistics background and references. 2022.https://www.gov.uk/government/statistics/soft-drinks-industry-levy-statistics/soft-drinks-industry-levy-statistics-background-and-references (accessed 10 Nov 2022).

9 Cabinet Office, Department of Health and Social Care, HM Treasury, *et al.* Childhood obesity: a plan for action. 2016.https://www.gov.uk/government/publications/childhood-obesity-a-plan-for-action (accessed 8 Apr 2021).

10 Department of Health and Social Care. Childhood obesity: a plan for action, chapter 2. 2018.

11 HM Revenue & Customs. Guidance: Check if you need to register for the Soft Drinks Industry Levy. 2018.https://www.gov.uk/guidance/check-if-you-need-to-register-for-the-soft-drinks-industry-levy (accessed 21 May 2023).

12 Gauke D. Budget 2016. 2016. https://www.gov.uk/government/publications/budget-2016-documents

13 HM Treasury, Osborne. G. Budget 2016: George Osborne's speech. 2016.https://www.gov.uk/government/speeches/budget-2016-george-osbornes-speech (accessed 6 Apr 2023).

14 Rogers NT, Cummins S, Forde H, *et al.* Associations between trajectories of obesity prevalence in English primary school children and the UK soft drink industry levy: an interrupted time series analysis of surveillance data. *medRxiv* Published Online First: 2022. doi:https://doi.org/10.1101/2022.09.16.22280030

15 Hilton S, Buckton CH, Patterson C, *et al.* Following in the footsteps of tobacco and alcohol? Stakeholder discourse in UK newspaper coverage of the Soft Drinks Industry Levy. *Public Health Nutr* 2019;**22**:2317–28. doi:10.1017/S1368980019000739

16 Economics O. The Economic Impact of the Soft Drinks Levy, Final Report. *London: Oxford Economics* 2016.

For peer review only

For peer review only - http://bmjopen.bmj.com/site/about/guidelines.xhtml

17 Penney TL, Adams J, White M. Industry reactions to the UK Soft Drinks Industry Levy: Unpacking the evolving discourse from announcement to implementation. *J Epidemiol Community Health (1978)* 2018;**72**:A43. doi:10.1136/jech-2018-SSMabstracts.88

18 Law C, Cornelsen L, Adams J, *et al.* An analysis of the stock market reaction to the announcements of the UK Soft Drinks Industry Levy. *Econ Hum Biol* 2020;**38**:100834. doi:10.1016/j.ehb.2019.100834

19 Law C, Cornelsen L, Adams J, *et al.* The impact of UK Soft Drinks Industry Levy on manufacturers' domestic turnover. *Econ Hum Biol* 2020;:100866.

20 Penney T, Adams J, White M. LB4 Industry reactions to the UK soft drinks industry levy: unpacking the evolving discourse from announcement to implementation. BMJ 2018. A43.1-A43. doi:10.1136/jech-2018-ssmabstracts.88

21 Hilton S, Buckton CH, Katikireddi SV, *et al.* Who says what about sugar-sweetened beverage tax? Stakeholders' framing of evidence: a newspaper analysis. *The Lancet* 2017;**390**:S44. doi:10.1016/s0140-6736(17)32979-3

22 Buckton CH, Hilton S, Patterson C, *et al.* DID PROPONENTS AND OPPONENTS OF THE SOFT DRINKS INDUSTRY LEVY USE THE NEWS MEDIA TO INFLUENCE THE POLICY DEBATE? A QUALITATIVE DISCOURSE ANALYSIS USING PRACTICAL REASONING. *J Epidemiol Community Health (1978)* 2018;**72**:A1–2. doi:10.1136/jech-2018-SSMabstracts.2

23 Buckton CH, Patterson C, Hyseni L, *et al.* The palatability of sugar-sweetened beverage taxation: A content analysis of newspaper coverage of the UK sugar debate. *PLoS One* 2018;**13**:1–15. doi:10.1371/journal.pone.0207576

24 Forde H, Penney TL, White M, *et al.* Understanding Marketing Responses to a Tax on Sugary Drinks: A Qualitative Interview Study in the United Kingdom, 2019. *Int J Health Policy Manag* Published Online First: 23 February 2022. doi:10.34172/ijhpm.2022.5465

25 Braun V, Clarke V. *Successful Qualitative Research: A Practical Guide for Beginners.* SAGE 2013.

26 White M. Evaluation of the health impacts of the UK Treasury Soft Drinks Industry Levy (SDIL). 2017;:1–46.https://www.journalslibrary.nihr.ac.uk/programmes/phr/1613001/#/

27 Harvey WS. Methodological Approaches for Interviewing Elites. *Geogr Compass* 2010;**4**:193–205. doi:10.1111/j.1749-8198.2009.00313.x

For peer review only - http://bmjopen.bmj.com/site/about/guidelines.xhtml

28 Berry JM, Browne W. Validity and Reliability Issues In Elite Interviewing. *PS Polit Sci Polit* 2003;**35**:679–82.

29 Harvey WS. Strategies for conducting elite interviews. *Qualitative Research* 2011;**11**:431–41. doi:10.1177/1468794111404329

30 Goldstein K. Getting in the Door: Sampling and Completing Elite Interviews. *Political Science & Politics* 2002;**35**:669–72. doi:10.1017/S1049096502001130

31 Stephens N. Collecting data from elites and ultra elites: Telephone and face-to-face interviews with macroeconomists. *Qualitative Research* 2007;**7**:203–16. doi:10.1177/1468794107076020

32 Braun V, Clarke V. Using thematic analysis in psychology. *Qual Res Psychol* 2006;**3**:77–101.

33 Braun V, Clarke V. Can I use TA? Should I use TA? Should I not use TA? Comparing reflexive thematic analysis and other pattern-based qualitative analytic approaches. *Couns Psychother Res* 2020;:37–47. doi:10.1002/capr.12360

34 Braun V, Clarke V. What can 'thematic analysis' offer health and wellbeing researchers? *Int J Qual Stud Health Well-being* 2014;**9**. doi:10.3402/qhw.v9.26152

35 Department of Health and Social Care. Calorie labelling in the out of home sector: implementation guidance. 2021.https://www.gov.uk/government/publications/calorie-labelling-in-the-out-of-home-sector/calorie-labelling-in-the-out-of-home-sector-implementation-guidance#:~:text=The%20out%20of%20home%20sector%20is%20generally%20considered%20to%20be,on%20or%20off%20the%20premises (accessed 14 Dec 2022).

36 Department for Digital Culture Media & Sport, Department of Health & Social Care. Introducing a total online advertising restriction for products high in fat, sugar and salt (HFSS). 2021.https://www.gov.uk/government/consultations/total-restriction-of-online-advertising-for-products-high-in-fat-sugar-and-salt-hfss/introducing-a-total-online-advertising-restriction-for-products-high-in-fat-sugar-and-salt-hfss (accessed 6 Jan 2023).

37 Office for Health Improvement and Disparities. Sugar, salt and calorie reduction and reformulation. 2017.https://www.gov.uk/government/collections/sugar-reduction (accessed 11 Jan 2023).

38 Stewart H. 'Sin taxes': Boris Johnson vows to review sugar levy. The Guardian. 2019.https://www.theguardian.com/politics/2019/jul/03/boris-johnson-vows-to-review-whether-sugar-tax-improves-health (accessed 19 Oct 2022).

For peer review only - http://bmjopen.bmj.com/site/about/guidelines.xhtml

For peer review only

39 Forde H, Penney T, White M, *et al.* A Framework for Understanding Marketing Responses to a Tax on Sugary Drinks: a Qualitative Interview Study in the United Kingdom, 2019. *Int J Health Policy Manag* 2022.

40 Lacy-Nichols J, Marten R, Crosbie E, *et al.* The public health playbook: ideas for challenging the corporate playbook. *Lancet Glob Health* 2022.

41 Wiist WH. The corporate play book, health, and democracy: the snack food and beverage industry's tactics in context. *Sick societies: Responding to the global challenge of chronic disease* 2011;:204–16.

42 Petticrew M, Katikireddi SV, Knai C, *et al.* 'Nothing can be done until everything is done': The use of complexity arguments by food, beverage, alcohol and gambling industries. *J Epidemiol Community Health (1978)* 2017;**71**:1078–83. doi:10.1136/jech-2017-209710

43 Theis D, White M. Is Obesity Policy in England Fit for Purpose? Analysis of Government Strategies and Policies, 1992–2020. *Milbank Quarterly* 2021;**99**:126–70. doi:10.1111/1468-0009.12498

44 Forde H, Penney T, White M, *et al.* Understanding Marketing Responses to a Tax on Sugary Drinks: a Qualitative Interview Study in the United Kingdom, 2019. *Int J Health Policy Manag* 2022.

45 Scarborough P, Adhikari V, Harrington RA, *et al.* Impact of the announcement and implementation of the UK Soft Drinks Industry Levy on sugar content, price, product size and number of available soft drinks in the UK, 2015-19: A controlled interrupted time series analysis. *PLoS Med* 2020;**17**:e1003025.

46 Boseley S. Jamie Oliver's Sugar Rush: a crusade to save Britain's health. The Guardian. 2015.

47 Tedstone A, Targett V, Allen R. Sugar Reduction: The evidence for action About Public Health England. *Public Health England* Published Online First: 2015.www.gov.uk/phe%5Cnwww.facebook.com/PublicHealthEngland

48 World Health Organization. Sugars intake for adults and children. 2015. https://www.who.int/publications/i/item/9789241549028 (accessed 14 Oct 2022).

49 Nieburg O. Kellogg shuns 'silver bullet' 30% UK cereals sugar cap. bakeryandsnacks.com. 2013.https://www.bakeryandsnacks.com/Article/2013/01/07/Kellogg-shuns-30-sugar-cap-proposal-for-UK-cereals (accessed 6 Jan 2023).

For peer review only

50 Rose D, Theis Z. What influences government policymaking? The case of childhood obesity in England. 2022.

51 Forde H, Boyland EJ, Scarborough P, *et al.* Exploring the potential impact of the proposed UK TV and online food advertising regulations: a concept mapping study. *BMJ Open* 2022;**12**. doi:10.1136/bmjopen-2021-060302

For peer review only

For peer review only - http://bmjopen.bmj.com/site/about/guidelines.xhtml

BMJ Open

Industry views of the UK Soft Drinks Industry Levy: a thematic analysis of elite interviews with food and drink industry professionals, 2018-20

Journal:	*BMJ Open*
Manuscript ID	bmjopen-2023-072223.R2
Article Type:	Original research
Date Submitted by the Author:	06-Jul-2023
Complete List of Authors:	Jones, Catrin; University of Cambridge, MRC Epidemiology Unit<br>Forde, Hannah; University of Cambridge, MRC Epidemiology Unit; University of Oxford, Nuffield Department of Primary Care Health Sciences<br>Penney, Tarra; York University, Global Food System and Policy Research, School of Global Health, Faculty of Health<br>Theis, Dolly; University of Cambridge, MRC Epidemiology Unit<br>Cummins, Steven; London School of Hygiene & Tropical Medicine, Population Health Innovation Lab, Department of Public Health, Environments & Society, Faculty of Public Health and Policy<br>Adams, J; University of Cambridge, MRC Epidemiology Unit<br>Law, Cherry; University of Reading, Department of Agri-Food Economics and Marketing<br>Rutter, Harry; University of Bath Department of Social and Policy Sciences, Social and Policy Sciences<br>Smith, Richard ; University of Exeter, Faculty of Health and Life Sciences<br>White, Martin; University of Cambridge, MRC Epidemiology Unit
**Primary Subject Heading**:	Health policy
Secondary Subject Heading:	Qualitative research
Keywords:	Health policy < HEALTH SERVICES ADMINISTRATION & MANAGEMENT, Public health < INFECTIOUS DISEASES, QUALITATIVE RESEARCH

SCHOLARONE™
Manuscripts

I, the Submitting Author has the right to grant and does grant on behalf of all authors of the Work (as defined in the below author licence), an exclusive licence and/or a non-exclusive licence for contributions from authors who are: i) UK Crown employees; ii) where BMJ has agreed a CC-BY licence shall apply, and/or iii) in accordance with the terms applicable for US Federal Government officers or employees acting as part of their official duties; on a worldwide, perpetual, irrevocable, royalty-free basis to BMJ Publishing Group Ltd ("BMJ") its licensees and where the relevant Journal is co-owned by BMJ to the co-owners of the Journal, to publish the Work in this journal and any other BMJ products and to exploit all rights, as set out in our licence.

The Submitting Author accepts and understands that any supply made under these terms is made by BMJ to the Submitting Author unless you are acting as an employee on behalf of your employer or a postgraduate student of an affiliated institution which is paying any applicable article publishing charge ("APC") for Open Access articles. Where the Submitting Author wishes to make the Work available on an Open Access basis (and intends to pay the relevant APC), the terms of reuse of such Open Access shall be governed by a Creative Commons licence – details of these licences and which Creative Commons licence will apply to this Work are set out in our licence referred to above.

Other than as permitted in any relevant BMJ Author's Self Archiving Policies, I confirm this Work has not been accepted for publication elsewhere, is not being considered for publication elsewhere and does not duplicate material already published. I confirm all authors consent to publication of this Work and authorise the granting of this licence.

For peer review only

Industry views of the UK Soft Drinks Industry Levy: a thematic analysis of elite interviews with food and drink industry professionals, 2018-20

Dr Catrin P Jones[a*], Dr Hannah Forde [a,<], Dr Tarra L Penney[b], Dr Dolly Theis [a], Prof Steven Cummins[c], Prof Jean Adams [a], Dr Cherry Law[d], Prof Harry Rutter[e], Prof Rich Smith[f], Prof Martin White[a].

a MRC Epidemiology Unit, School of Clinical Medicine, University of Cambridge, United Kingdom

b Global Food System and Policy Research, School of Global Health, Faculty of Health, York University, Canada

c Population Health Innovation Lab, Department of Public Health, Environments & Society, Faculty of Public Health and Policy, London School of Hygiene & Tropical Medicine, United Kingdom

d Department of Agri-Food Economics and Marketing, University of Reading, Reading, UK

e Department of Social and Policy Sciences, University of Bath, Bath, UK

f Faculty of Health and Life Sciences, University of Exeter, Exeter, UK

For peer review only

For peer review only - http://bmjopen.bmj.com/site/about/guidelines.xhtml

< Present address: Nuffield Department of Primary Care Health Sciences, University of Oxford, Oxford, United Kingdom

***Corresponding Author:** Dr Catrin Pedder Jones, MRC Epidemiology Unit, University of Cambridge School of Clinical Medicine, Box 285 Institute of Metabolic Science, Cambridge Biomedical Campus, Cambridge, CB2 0QQ, United Kingdom. Catrin.jones@mrc-epid.cam.ac.uk +44 (0)1223 769 114

Keywords: Soft drinks, Soft drinks industry levy, Food and drink industry, sugar sweetened beverage

Word count: *6550*

Abstract

Introduction: The UK Soft Drinks Industry Levy (SDIL), implemented in 2018, has been successful in reducing the sugar content, and purchasing, of soft drinks, with limited financial impact on industry. Understanding the views of food and drink industry professionals involved in reacting to the SDIL is important for policymaking. However, their perceptions of the challenges of implementation and strategic responses are unknown. The aim of this

For peer review only - http://bmjopen.bmj.com/site/about/guidelines.xhtml

study therefore was to explore how senior food and drink industry professionals viewed the SDIL. ***Methods and analysis:*** We undertook a qualitative descriptive study using elite interviews with 14 senior professionals working in the food and drink industry. Braun and Clarke's approach to thematic analysis was used to analyse the data, taking an inductive exploratory and descriptive approach not informed by prior theory or frameworks. Five main themes were identified: *(1) A level playing field…for some*; Industry accepted the SDIL as an attempt to create a level playing field but due to the exclusion of milk-based drinks, this was viewed as inadequate. *(2) Complex to implement, but no lasting negatives*; The SDIL was complex, expensive and time consuming to implement, with industry responses dependent on leadership buy-in. "(3) *Why us? – the SDIL unfairly targets the drinks industry*"; soft drinks are an unfair target when other categories also contain high sugar. "(4) *The consumer is king";* Consumers were a key focus of the industry response to this policy. "(5) *The future of the SDIL*"; There appeared to be a wider ripple effect, which primed industry to prepare for future regulation in support of health and environmental sustainability. ***Ethics and dissemination:*** The study was approved by the Humanities and Social Science Ethics Committee at the University of Cambridge, UK.

For peer review only

Strengths and Limitations

- This qualitative study explored how senior food and drink industry professionals viewed the SDIL.
- We undertook elite interviews with 14 professionals working in the food and drink industry, who have often been difficult to recruit in other studies.
- Braun and Clarke's thematic analysis taking a descriptive approach was used to analyse the data.
- Elite interviewing methods allow for the building of relationships to elicit meaningful responses from participants.
- Insights from senior food and drink industry professionals illustrate how sugar sweetened beverage taxes might be successfully implemented and improve understanding of industry responses to taxes and other food and drink policies.

For peer review only

Introduction

Diet-related non-communicable diseases are a major and growing problem, responsible for over 11 million deaths globally each year [1]. Sugar consumption is of particular concern, with the World Health Organization (WHO) recommending member states introduce sugar-sweetened beverage (SSB) taxes [2]. Reviews suggest that they reduce sales of, increases prices of and encourage reformulation of SSBs [3–5] and over 100 SSB taxes have been implemented worldwide covering 52% of the world's population [6]. SSB taxes have a variety of designs with 87% excise taxes [6]. The WHO recommend that a tiered SSB tax be introduced in companies with high administrative capacity, similar to that which has been introduced in the UK [2]. The Soft Drinks Industry Levy (SDIL) was announced on 16th March 2016 and implemented in the United Kingdom (UK) on 6th April 2018. According to the budget speech by George Osborne, Chancellor of the Exchequer at the time, it was designed to incentivise manufacturers of SSBs to reformulate their products [7] via charging a levy on soft drinks produced by companies when they leave the warehouse or when imported into the country [8]. Integrated in August 2016 as part of the UK Government's Childhood Obesity: A Plan For Action [9], the SDIL consists of two tiers (for particulars of the tax see box 1). A public consultation on the proposals between August - October 2016 set

For peer review only

out the plans for the tiers and exclusions as described in Box 1. Few changes were made as a result of this consultation and the SDIL was given royal assent on 27th April 2017. The Government published a second chapter of its childhood obesity plan in 2018, which suggested the SDIL may be extended to milk-based drinks, though this has not yet occurred [10].

For peer review only

Box 1: Soft Drinks Industry Levy Particulars [7]

Eligible drinks:

- ≥8 g total sugar per 100 mL charged at 24 pence per litre
- ≥5 g and <8 g total sugar per 100 mL charged at 18 pence per litre

Exemptions:

- Drinks containing more than 75% milk or 1.2% alcohol
- Alcohol substitute drinks
- Powdered drinks

For peer review only - http://bmjopen.bmj.com/site/about/guidelines.xhtml

- 100% fruit juices
- Manufacturers selling under one million litres of drinks per year[11]

The SDIL was one of the first SSB fiscal interventions explicitly designed to incentivise reformulation [7,12,13]. This aim was largely achieved, substantially reducing overall SSB sugar content, and inducing a major shift of drinks from the higher levy tier to the lower tier and untaxed bracket between 2016 and 2018 (Scarborough et al., 2020). Reformulation is reflected in purchases of sugar from SSBs, with [14]. Prior to implementation of the SDIL, the food and drinks industry (hereafter referred to as 'industry') viewed the SDIL as having a potentially negative impact on profits resulting in job losses [15–17]. A negative stock market reaction to the SDIL announcement was observed, but this only lasted two days [18]. Similarly, a negative impact on company domestic turnover was observed following the announcement of the SDIL, but this resolved by the time of its implementation [19].

Critical to the success of the SDIL is the implementation of and reaction to the regulations by the drink industry. Therefore, it is important to understand perspectives of the industry as well as those who work in it regarding the implementation of such taxes. Previous work has

For peer review only - http://bmjopen.bmj.com/site/about/guidelines.xhtml

For peer review only

investigated industry perspectives of the SDIL expressed through the news media [15,20–23] and the views of industry, civil society and academic participants on how marketing changed in response to the SDIL [24]. A notable gap in the literature, however, is perspectives of the SDIL from the commercial sector, not communicated publicly through news media nor focused solely on marketing responses. Important learning can be obtained by exploring the perspectives of commercial actors involved in responding to regulation. Interviews with senior members of industry can help examine the impact of the SDIL on both the soft drinks industry and wider food and drink industry, an avenue not previously explored. This study therefore aimed to address these knowledge gaps and inform policymaking by exploring the perspectives of senior industry professionals regarding the UK SDIL.

Methods

Study Design

This study adopted a qualitative descriptive design involving elite interviews with senior industry professionals.

Methodological Orientation

For peer review only - http://bmjopen.bmj.com/site/about/guidelines.xhtml

This research took an experiential qualitative approach, within a critical realist position. Participant perspectives and perceptions were prized over researcher interpretations, and reality was derived from our participants' words and meaning, rather than a reality constructed through researchers' interpretation of their words [25]. A descriptive approach was used to explore how the SDIL was viewed from the position of our participants.

Research Team

Prof Martin White (MW), Prof Steven Cummins (SC), Prof Jean Adams (JA), Prof Rich Smith (RS) and Prof Harry Rutter (HR) secured funding for the overall evaluation of the SDIL within which this study formed a part [26]. Interviews were conducted by Postdoctoral Research Associates Dr. Tarra L Penney (TLP) and Dr. Catrin P Jones (CPJ). TLP led the design of data collection and CPJ led the design of the analysis. MW & SC provided guidance on the design of both elements. TLP and CPJ recruited and interviewed participants. CPJ led the analysis with support from Dr Hannah Forde (HF). HF conducted secondary coding to support theme generation and interpretation. All authors previously mentioned, as well as Dr Dolly Theis (DT) and Dr Cherry Law (CL) were involved in data analysis and interpretation, as well as drafting this manuscript.

For peer review only

Participants

Senior professionals from the soft drinks, food and other drinks industries were recruited to this study using purposive and snowball sampling. We adopted 'elite interviewing' methods to maximise involvement of senior professionals in positions of influence within their organisation and with high levels of responsibility [27]. This technique provides a series of strategies to support recruitment of difficult to access key participants, and to ensure the validity and reliability of data [28]. The principles of elite interviewing were used to inform recruitment including stronger emphasis on the maintenance of trust, importance of interview tone of the interview, preparing appropriately, and engaging in and tailoring dialogue relevant to each informant, more so than in traditional interviews [29,30].

Individuals were considered eligible to participate based on the following criteria: a) currently or previously held a high-level industry position (at the managerial, director or chief officer level), b) their organisation and their professional role was directly or indirectly impacted by the SDIL and c) they could provide a novel perspective, determined by their job role or the company they work for not previously heard in our interviews, to ensure a range

For peer review only

of views. Recruitment typically involved an email introduction by a member of the team or informant contact, although CPJ also attended industry food events and recruited face to face. Initial contact was followed by an informal telephone conversation with TLP or CPJ to discuss the research purpose, team and informant interests and perspectives, ultimately proceeding to full participation via telephone interview.

Data Collection

Telephone interviews were conducted from June 2018 to June 2020. Participant information sheets were sent to potential participants prior to participating in the informal discussion. Informed consent was obtained verbally prior to commencement of the formal telephone interview, which was digitally audio-recorded. Interviews were undertaken using a minimally structured topic guide containing three broad areas of inquiry: a) Can you tell me about your role and organization? b) Can you tell me about your sector as a whole? c) What do you know about the UK Soft Drinks Industry Levy and its impacts? Elite interviewing necessitates informed and adaptive dialogue [29,31], meaning participants could engage in ways most relevant to their specific expertise or experiences within these broad areas. Interviews were transcribed verbatim by a trusted external company, and transcripts were

For peer review only - http://bmjopen.bmj.com/site/about/guidelines.xhtml

checked against the audio files by CPJ to identify any inaccuracies. Transcripts were anonymised prior to analysis by removing names of people, organisations, and brands.

Analysis

Analysis commenced once all interviews had been conducted and transcribed. Braun and Clarke's thematic analysis was used, taking an inductive exploratory and descriptive approach not informed by any prior theory or framework [32]. This approach is flexible due to lack of alignment with specific epistemological and ontological stance [33,34]. Six analytic steps were conducted: 1) familiarisation, 2) data coding, 3) initial theme generation, 4) theme development and review, 5) theme refining, defining and naming, 6) writing up.

CPJ listened to audio files and read transcripts at least twice to become familiar with them, whilst making notes on initial impressions and patterns (step 1). Following familiarisation, CPJ worked systematically through the entire data set and conducted complete coding of all data, in which segments of data were given a label to describe their area of interest. Coding was supported by NVivo software version 12. Semantic codes were derived directly from participants' speech or codes where phrases of speech were brief enough to be directly coded

For peer review only

(step 2). CPJ then sorted these initial codes into concise categories (overarching codes), which clearly described the content of the data (step 3). A reflective diary was kept throughout the coding process by CPJ to note reflections on findings and to ensure a data-driven analytic process. Please see supplementary file 1 for a detailed account of reflexivity.

HF also familiarised themself with the transcripts (step 1) and then examined CPJ's coding to ensure the codes were data driven with as little interpretation as possible (step 2). CPJ then collated codes that shared a common pattern into themes (step 3). Again, CPJ and HF met to discuss and refine the themes to ensure they were descriptive with minimal interpretation (step 4).

A document containing themes, codes within them, and extensive anonymised quotes was shared with all co-authors in two phases: phase 1 March 2022 and phase 2 October 2022 (step 5). This data clinic aimed to minimise researcher interpretation. A document presented theme descriptions and asked co-authors to answer the following questions for each theme: 1) Is the theme descriptive? 2) Does the theme represent the data accurately? 3) What do you think the theme tells us about the SDIL from the perspective of industry? JA, DT, CL

For peer review only

For peer review only - http://bmjopen.bmj.com/site/about/guidelines.xhtml

completed the data clinic document in phase 1. Themes were amended based on their reflections and the document updated in October 2022. SC, MW, HR & RS completed the data clinic form in phase 2. Final themes and the manuscript were written up by CPJ and reviewed by all co-authors (step 6).

Patient and Public Involvement Statement

This study is part of the 'Evaluation of the health impacts of the UK Treasury Soft Drinks Industry Levy (SDIL)' funded by NIHR (award no. 16/130/01). Project oversight is provided by an independent study steering committee (ISSC) which contains members of the public. The ISSC for the overall project met biannually from 2017 – 2023 and were asked to provide advice on methodology as well as interpretation of our findings.

Results

Fourteen participants were recruited (Table 1). Participants' roles within organisations were diverse; chief officers, directors, and managers with overall responsibility or with specialist responsibilities for finance, strategy, operations, marketing, public relations or nutrition. Interviews ranged in length from 26 to 62 minutes. Six additional participants were

For peer review only - http://bmjopen.bmj.com/site/about/guidelines.xhtml

approached and took part in informal discussions; three did not participate due to scheduling issues, and three refused to take part. Five inductively derived, interlinked themes and 15 subthemes were identified (Box 2).

For peer review only

Table 1: Participant details

**Sector Category**	**N**
Drink manufacturers	4
Food and drink manufacturers	3
Supermarkets	3
Industry associations	1
Out-of-home* food and drink manufacturers	1
Out-of-home retailers	1
Advertising consultants	1

*** "The out-of-home sector is generally considered to be any outlet where food or drink is prepared in a way that means it is ready for immediate consumption, on or off the premises"* [35]

Box 2: Theme and sub-theme summary.

For peer review only - http://bmjopen.bmj.com/site/about/guidelines.xhtml

Theme 1: A level playing field…for some<br>*The SDIL created a level playing field*<br>*Milk-based drinks increased the complexity in the out-of-home sector*<br>*Challenges for supermarkets with large product portfolios*<br>Theme 2: Complex to implement but no lasting negative effects<br>*Complexities in strategic response – price and product are key*<br>*Global companies and internal systems*<br>*Contradictory government messaging*<br>*Leadership buy-in dictates strategic response*<br>*Few long-lasting negative effects & SDIL provided opportunities*<br>Theme 3: Why us? – The SDIL unfairly targets the drinks industry<br>*Sugary drinks in isolation were unfair targets for regulation*<br>*Distrust of government's motivations to introduce the SDIL*<br>Theme 4: Consumer is king<br>*Consumer response to product changes resulting from the SDIL*<br>*Consumer momentum towards healthier products*<br>Theme 5: The future of the SDIL<br>*Extending to milk and fruit-based drinks*<br>*Impact on the wider food and drink industry and on other sectors*<br>*Proposal to reverse the SDIL*

For peer review only

Theme 1: A level playing field…for some

The SDIL created a level playing field

Industry professionals accepted that the SDIL helped create a level playing field, where no organisation lost out by taking action on health that their competitors did not *"… legislation*

For peer review only - http://bmjopen.bmj.com/site/about/guidelines.xhtml

level playing fields is so important and that's why with these big public health initiatives…I'm actually really quite pro government intervention" – Supermarket. Soft drinks manufacturers also discussed that the two years to prepare for the implementation of the SDIL was sufficient and they were happy they could develop an adequate response within that time. *"I'm not aware of any significant implementation or challenges that our members have encountered, I mean they did have time to adapt, the legislation was published in good time to allow them to understand exactly what they would be required to do" – Trade association*

However, participants also stated that a lack of understanding and consultation from government meant a 'true' level playing field for all sectors involved in the sales of sugary drinks had not been not achieved *"…you want to really do it smartly so everybody feels they're 100% equally affected and you don't get this…'my product is in scope, your product is out of scope'…it doesn't create the sense of unilateral 'let's do this'…which is what it should be, if that makes sense" – Food and drink manufacturer.* The lack of consultation by the government with sectors who were not soft drinks manufacturers (for example out of home retailers) and the exclusion of milk-based sugary drinks led to this perception *"…milk-*

For peer review only

based drinks often carried bigger serving sizes and had more total sugar in them than any of our products would. They were excluded from the levy as well which looked like a big shortcoming." Drink manufacturer.

Milk-based drinks increased the complexity in the out-of-home sector

Interviewees explained that, from their perspective, the government did not think clearly about the technical implications for retailers and out-of-home sector and that it was easier for soft drink manufacturers to respond to the levy than it was for other industry actors *"…I don't think they understood the ways of working and the preparation methods in the out-of-home sector…" – Out-of-home food and drink manufacturer.* A high level of complexity within the out-of-home sector to manufacture and produce drinks for immediate consumption led to higher implementation costs; specifically, the exclusion of milk-based drinks and specification around eligibility of drinks mixed with carbon dioxide, water and ice, and those with and without milk. *"…they were looking at the likes of drinks fountains for carbonated soft drinks because… a bag and box syrup, they would be mixed with ice or carbon dioxide to give the carbonation or either they could be mixed with water and that would capture those drinks in the out-of-home sector, but there was a vagueness to milk-based drinks" – Out-of-*

For peer review only

home food and drink manufacturer. Some queries to Her Majesty's Revenue and Customs (HMRC – the tax collecting authority in the UK) went unanswered, thus, the out-of-home sector had to interpret the legislation themselves and apply the SDIL according to their interpretation. Representatives of the out-of-home sector did not perceive two years as enough time to have prepared due to confusion surrounding eligibility. In contrast, soft drinks manufacturers stated they had had time to prepare.

Challenges for supermarkets with large product portfolios

Supermarkets felt disadvantaged compared to soft drink manufacturers by the complexities of their sector. They highlighted sector-specific challenges to adapting to the SDIL, including that their product portfolio not only contains branded drinks, about which they have to make decisions, but also private label (own brand) drinks *"…what branded suppliers chose to do was their choice…different brands choosing to reformulate, resize or inflate, which I think led to a fair bit of customer confusion as to what the hell was going on. " – Supermarket*. It was described as challenging and time consuming to manage such a large portfolio and make decisions on each product. Particularly as reformulation decisions and portion size reduction reportedly differed between brands yet had to be merchandised together within stores.

For peer review only - http://bmjopen.bmj.com/site/about/guidelines.xhtml

Retailers also felt that they were disadvantaged as their customers expressed confusion at differing responses by different brands – e.g. 'sugary' drinks reformulated to just below the SDIL threshold but containing both sugar and sweeteners confused customers, with queries directed at retailers rather than drinks manufacturers *"… we tried to make it as clear for customers by putting on all the [shelves] sugar levy applied, so they could very much see…But…when they see a sugar line that's not [included in the SDIL], that's when the questions start coming." – Supermarket.*

Theme 2: Complex to implement but no lasting negative effects

Complexities in strategic response – price and product are key

Industry responded to the SDIL by reviewing product portfolios and strategically selecting responses at the individual product level. This portfolio review approach is why responses differed between companies and between products. Research and development (R&D) and consumer testing were costly for industry during this process, and, linking to theme 1, there were increased costs for those companies with larger product portfolios (e.g. supermarkets). For the out-of-home sector, additional complications were noted due to confusion over eligibility of some milk-based drinks *"…government is very keen to always say "oh just*

For peer review only

reformulate, it will be easy" but it's not easy. It actually takes a lot of time and investment." – Drink manufacturer.

Consumer testing was vital during the reformulation and decision-making process and consumer preference dictated the strategy taken *"… we invested a significant amount of money…in developing lots and lots of different formulations with lower sugar to see and testing them with consumers in Great Britain to see whether those recipes…would be acceptable to consumers." – Drink manufacturer.* An additional challenge in reformulating drinks described by manufacturers was that sugar serves a functional purpose, in the mouthfeel of drinks mixed with ice and to prevent 'brain freeze', as well as to provide sweetness *"Because, actually, yes, we could stick sweeteners in everything, but, actually, sugar also has like a functional role" – Out-of-home retailer.*

For peer review only

Packaging, merchandising and placement were challenges to overcome, particularly for supermarkets. Decisions were made on own brand products but also on how to retail other branded products with different responses to the SDIL (e.g. reformulated drinks, reduced and increased portion sizes, rebranding) *"…there were a number of products that didn't*

For peer review only - http://bmjopen.bmj.com/site/about/guidelines.xhtml

reformulate but did drop size. So, again, there's just small considerations in that around how you merchandise it… So what sounds like a relatively simple change, of dropping from 330ml to, I don't know, 250ml, in reality kind of that complexity flows back through the value chain" – Supermarket.

Leadership buy-in dictates strategic response

Leadership buy-in to health, where senior management 'buy-in' to the idea that their company should be making pro-health decisions, was discussed as vital in dictating the strategic response to the SDIL *"… I think such a review requires strong leadership and … our COO was very clear that we needed to step in and we needed to do, you know, do the responsible, brave thing." -Drink manufacturer*. Participants described this buy-in as making the process simpler and a lack of buy-in as a barrier to making timely progress *"… having that strong leadership and, you know, complete buy-in from the top team and actually pretty much all the other levels of the organisation, then it's actually quite simple" – Drink manufacturer.*

Global companies and internal systems

For peer review only

The cost of setting up internal systems to account for and pay the SDIL was expensive, due to the requirement to report to HMRC, regardless of whether or not a company involved in the manufacture or selling of soft drinks was liable to pay the levy *"…It's ridiculous that, you know, it's cost us half a million pounds just to tell Treasury that actually we don't need to pay it." – Drink manufacturer.* The global nature of many of these companies was an additional challenge. Response strategies appropriate for a UK market may not be transferable to other countries, for example reformulation recipes vary due to differences in consumer palate and storage temperatures/facilities *"…that's [computer system] for the UK, and then Ireland have a separate system, France have a separate system, Mexico have a separate system. " – Food and drink manufacturer.*

Contradictory government messaging

There was confusion over whether manufacturers needed to pass on price increases to change consumer behaviour due to contradictory government messaging over the aim of the SDIL. Participants indicated that they thought price increases should have been passed on to target individual behaviour change; however, manufacturers stated they had no control over whether this occurred as retailers set the price for consumers *"…[the] government had*

For peer review only - http://bmjopen.bmj.com/site/about/guidelines.xhtml

slightly mixed messages so it was pretty clear from the Department of Health and PHE [Public Health England] *… that they expected to see prices passed on … I think the Treasury were trying to say, oh soft drinks manufacturers don't have to pass this on... Well, apart from the fact that most businesses won't absorb a cost if they can avoid it for obvious reasons, it was the opposite of what the Health Department and others wanted…" – Drink manufacturer.*

Few long-lasting negative effects & SDIL provided opportunities

Participants acknowledged that the SDIL did achieve its aim in stimulating product reformulation to avoid the levy. Although implementation was complex and costly, as previously illustrated, there were few long-lasting negative effects. Some participants suggested the SDIL provided opportunities *"I think some of them would have switched back but we've gained new consumers as well which is, you know, how we, which through sampling and advertising essentially." – Drink manufacturer*. However, participants were sceptical that the SDIL would achieve intended reductions in childhood obesity in the UK. *"… why [the SDIL] it was thought that that would be a, that policy in isolation would be sufficient to reduce obesity rates." – Drink manufacturer.*

For peer review only - http://bmjopen.bmj.com/site/about/guidelines.xhtml

Theme 3: Why us? – The SDIL unfairly targets the drinks industry

Sugary drinks in isolation were unfair targets for regulation

Participants felt that the SDIL unfairly targets the soft drinks industry. Participants expressed their frustration that a single food category was targeted when other food categories bear a significant proportion of the responsibility for childhood obesity. They expressed the view that multiple nutrients or calories across many food and drink sectors should be targeted by regulation if the government is serious about reducing childhood obesity, particularly as substitution to other non-regulated food categories could negate the impact of the SDIL on health *"…why would it be just the soft drink levy, why would you not target cakes and biscuits…that's what we didn't understand at the time." - Food and drink manufacturer..*

There was consensus among participants that it did not make sense for the government to target a category that they considered was already reducing sugar faster than other food categories. Although the SDIL had accelerated the reformulation progress for some, this was stated to be already occurring prior to the SDIL announcement. Participants expressed the view that the sector had been unfairly penalised, and that sectors which reformulate should be praised rather than targeted by regulation when other unregulated categories have contributed

For peer review only - http://bmjopen.bmj.com/site/about/guidelines.xhtml

little towards achieving health goals. *"… the soft drinks category was already well embarked on the journey to reformulation…part of the industry's disappointment and frustration about the announcement of the levy was that they were already absolutely going to deliver what the levy has now kind of made them deliver" – Trade association*

Distrust of government's motivations to introduce the SDIL

Participants stated that the SDIL was politically motivated, not an evidence-based policy. Government policies targeting obesity were described as contradictory and not aligned with one another, particularly the proposed ban on advertising of less healthy foods on TV and online [36]. According to participants, the advertising ban does not distinguish between reformulated and non-reformulated products, and acts as a disincentive to spending on reformulation if they cannot recoup their investment through advertising new products. *"So if you can take something from 40g of sugar to 20g of sugar but you'd only advertise on TV is it's 5(g), then why bother, right, and it also means that they can't tell the world, look at this amazing thing we've done, we've reformulated this" – Advertising consultant.*

For peer review only

Perceived disconnectedness between policies led to distrust in the government and a belief that government obesity policy is poorly planned. Distrust was compounded by some companies appearing to be successful at lobbying the government following the announcement, resulting in changes to the regulations as a result of this lobbying, rather than on the basis of health or nutrition, in particular the decision to exclude milk-based drinks. Participants stated this was motivated by some companies being able to gain a competitive advantage, as some milk-based drinks have higher sugar content than soft drinks. Participants also referred to the SDIL as a political tool to distract from other things in the budget in which it was announced *"… I think this was a decision taken within the Treasury by quite a small group of people and it was announced during a Budget by a Chancellor who was trying to distract from some other economic figures that he maybe wasn't too pleased about." – Drink manufacturer*. The fact that the proposal to establish the SDIL had been kept secret, and the announcement was a shock to many, led to this view. *"I think the timing was a surprise... Yeah and the way it was done without any form of consultation or pre-announcement." – Drink manufacturer.*

Theme 4: Consumer is king

For peer review only - http://bmjopen.bmj.com/site/about/guidelines.xhtml

Consumer response to product changes resulting from the SDIL

Industry participants discussed throughout all previous themes that meeting the wishes of consumers was the priority when responding to the SDIL. Taste preferences and tolerance of reformulation changes were critical and companies expressed concerns that consumers might dislike reformulated products if they changed dramatically in a short time period *"… obviously what's critical from our perspective is developing a product that consumers still like the taste of whilst reducing their sugar intake so that we were trying to marry-up those two things." – Drink manufacturer*. Company responses to the SDIL, as well as health and environmental issues more broadly, were vital to maintaining brand loyalty and company reputation in the eyes of consumers. The media were seen as influential in shaping consumer preferences and company reputation, as some newspapers had used graphics to show the sugar content of drinks and this was considered to have influenced purchasing patterns. According to informants a small group of very loyal consumers can cause a backlash publicly, which can be picked up by both the news media and social media.

Consumer momentum towards healthier products

For peer review only - http://bmjopen.bmj.com/site/about/guidelines.xhtml

Participants stated consumer purchasing patterns are changing, with consumers increasingly choosing lower sugar products, which may also have driven reformulation prior to the SDIL. The policy acted as a catalyst for increasing consumer demand for sugar reduction and some respondents also highlighted the role of social media in driving these trends. Consumers were also reported by participants as “moving away from" artificial ingredients, which leds to challenges in reformulation using non-nutritive sweeteners *"A lot of our consumers like ..., , they don’t want to have sweeteners, they don’t want to have preservatives" – Drink manufacturer*. Some participants suggested that consumers were not lost when sugar was reduced in their favourite products, due to consumer preferences moving towards prioritising health. It was important to participants and their organisations that consumers have enough choice and there were concerns that regulation could limit choice from some.

Theme 5: The future of the SDIL

Participants discussed the potential of expanding the SDIL to fruit and milk-based drinks, the wider threat to other products, reformulation in other categories, changes in other sectors as a result of the SDIL and the possibility of its reversal by government.

For peer review only - http://bmjopen.bmj.com/site/about/guidelines.xhtml

Extending to milk and fruit-based drinks

Concerns were expressed over the Chancellor's proposal to extend the SDIL to milk-based and fruit-based drinks at the time of the announcement *"I don't think politicians think it's done. Obviously we've got the review next year on whether milk-based drinks should be included, and then I think it's 2021 when they'll review the levels as well." – Drink manufacturer*. Participants stated the nutritional benefits of these meant that natural sugars (fructose and lactose) should not be subject to the same regulation as soft drinks. The vitamin and mineral content of these drinks was also discussed as a benefit to children who may not be consuming sufficient fruit, vegetables or calcium from other sources *"… Now you have products that are being developed with high levels of sugar in them so that really does need to be addressed but you don't want to go down the route of demonising milk because it is still a great source of nutrition." – Out-of-home food and drink manufacturer*. Reformulation of these drinks was considered particularly challenging, as naturally occurring sugars cannot be removed in the same way as added sugars in soft drinks.

Impact on the wider food and drink industry and in other sectors

For peer review only - http://bmjopen.bmj.com/site/about/guidelines.xhtml

A wider threat to other products, particularly those included in the PHE Sugar Reduction Strategy [37] (another element of the Childhood Obesity Plan that encouraged voluntary industry reformulation) was discussed. The SDIL demonstrated that the government was willing to implement policy to regulate the food industry in a way that has not been done before. Food and drink companies discussed their companies' attempts to reformulate products not included in the SDIL. The SDIL was described as a rallying call for industry to improve the healthfulness of products. It was also perceived to cause a ripple effect not just regarding health but also sustainability, environment, media and promotions. *"Yeah, I think there is a ripple effect. So, I think it can be both positive and negative. I think in terms of positive, I think it can force companies to reformulate and be more innovative in driving the use of other ways of sweetening products" - Food and drink manufacturer*

Proposal to reverse the SDIL

Comments made by Boris Johnson in his leadership campaign to become prime minister (July 2019), suggested he might consider repealing the SDIL [38]. These were not taken well by some participants; who indicated that companies had invested heavily in implementing the levy *"I suppose it does feel like a backtrack [reversing the SDIL]. Like we've made all this*

For peer review only

work and it was at the time quite painful in the sense of it was such a massive change through the supply chain so there was so many things to think about" – Out-of-home food and drink manufacturer. However, some participants suggested that reversing the SDIL would be well tolerated. *"I think, yeah, the industry would be happy to see the back of it because it's just cumbersome, it's just something, it's just another thing to administer." - Food and drink manufacturer.*

Discussion

Summary

Senior industry perspectives on the SDIL are described in five main themes. Theme 1: *A level playing field…for some*, Theme 2: *Complex to implement, but no lasting negatives,* Theme 3: *Why us? – the SDIL unfairly targets the drinks industry,* Theme 4: *The consumer is king*, and Theme 5: *The future of the SDIL.* The SDIL appeared to create a level playing field which industry accepted, however, this was perceived as inadequate due to the exclusion of milk-based drinks and targeting only SSBs, giving some a competitive advantage. Implementation of the SDIL was time consuming and complex, leading to high financial investment to prepare for it. Strategic response to the SDIL was dependent on leadership buy-

For peer review only - http://bmjopen.bmj.com/site/about/guidelines.xhtml

in and particularly governed by potential consumer responses to product changes associated with the policy. The announcement and subsequent implementation of the SDIL caused a ripple effect beyond the soft drinks industry. The wider food and drink industry perceived it as evidence of the government being willing to regulate to help achieve health goals.

Strengths and limitations

The use of elite interviewing techniques to build relationships with and solicit meaningful responses from participants is a strength of this work. These techniques allowed us to obtain the views of senior professionals from commercial organisations who have often been difficult to recruit to other studies [39]. As evident from the challenges described in the out-of-home sector and supermarkets, including respondents outside of manufacturing allowed wider exploration of the systemic impacts of the SDIL. A limitation of this work, however, is that interviews were carried out over a long period of time due to challenges in recruitment. Therefore, not all participants experienced the same political context, such as Boris Johnson's threats to reverse the SDIL in July 2019. Initial plans were for longitudinal data collection repeated across the time period of the study. Had all participants been interviewed closer to the implementation of the SDIL in 2018, then repeated in 2020, perspectives on the political

For peer review only - http://bmjopen.bmj.com/site/about/guidelines.xhtml

events occurring would have been captured from all participants. Unfortunately, challenges to recruitment and access to elite participants led to the abandonment of this plan. Although researcher neutrality was expressed to participants the position of interviewers as public health academics could have led to these recruitment challenges.

The positionality of the researchers may also have led to censoring of responses by some participants. Whilst we sought to descriptively represent industry perspectives, as well as acknowledge our own biases that are typically pro-health policy, it is important to acknowledge that the food and drink industry will have their own biases against health policy that is detrimental to their business survival, as evidenced in previous work [40,41]. Although it was not the aim of the work to explore participant responses in relation to the commercial determinants of health, it is possible that participant responses did not represent the reality of what occurred behind the scenes in the food and drink industry in relation to the SDIL. Overlap between some of the responses provided in this work and the 'typical' responses explored by other researchers as an industry 'playbook' [42] may support this assertion.

Interpretation and implications

For peer review only - http://bmjopen.bmj.com/site/about/guidelines.xhtml

Interviewees reported that the technical aspects of drink production, particularly in the out-of-home sector, were not adequately accounted for in the design of the SDIL. An unintended consequence of the milk-based drink exclusion, led to some organisations having to interpret the particulars of the SDIL whilst their queries to HMRC went unanswered. Experiences of participants in this work align with findings that UK Government policy is set up poorly for the purposes of adequate monitoring and evaluation [43]. Future policy should engage with the wider food and beverage sector once a policy is certain to be implemented, to design and communicate technicalities in ways that avoid industry having to interpret themselves what is required and provide timely responses to queries surrounding implementation. Further, respondents indicated that lobbying against the inclusion of milk-based sugar sweetened beverages in the SDIL resulted in this exclusion. Alongside policy engagement in the technicalities of production, an avenue for future research would be to understand in more detail the policy process surrounding the SDIL, particularly the influence of the food and drink industry on the policy particulars.

Reviewing their product portfolio was also discussed, where assessments of the product mix as a whole and by individual product were conducted when determining the response to the

For peer review only

SDIL. This aligns with previous findings that soft drink companies monitor their internal and external contexts to determine their products' market position in response to a stimulus such as the SDIL, and then respond with marketing or non-marketing activity to influence the purchasing of soft drinks [44]. A crucial external contextual component to response in our findings appears to be consumer response and preferences towards each product, as well as health as a whole.

The UK soft drinks industry was reformulating products to lower sugar alternatives several years before the SDIL was introduced [44,45]. Perspectives expressed by participants align with this and suggest that there is a shift towards healthier drinks as the primary offer for consumers, with the SDIL accelerating the pace of this change. Consumer preferences for healthier products, and our finding that industry prioritises these health preferences in their decision making, are likely to have triggered the soft drinks industry to reformulate products prior to the announcement of the SDIL. The advocacy (e.g. Jamie Oliver and Action on Sugar) in the early 2010s [46–48]and government threats to regulate industry [49] may have also increased consumer awareness about the health impact of sugar consumption and had a 'signalling effect' to consumers to reduce their sugar consumption [50]. Participants in our

For peer review only

For peer review only - http://bmjopen.bmj.com/site/about/guidelines.xhtml

study suggested that the SDIL was adopted by the Government because of the existing popularity of sugar reduction among the public. It is likely that the UK public was aware that SSBs harm health much earlier than the policy announcement, resulting from media activity, such as that related to Jamie Oliver's campaigning [46] PHE's [47]and WHO's reports on sugar [48]. Therefore, the importance of public momentum towards health could be regarded as a trigger for industry action independently from encouraging government action via policy.

Finally, participants expressed concern that policies introduced to combat obesity and other societal issues should be complementary not contradictory. The proposed ban on TV and online advertising of high fat, salt and sugar (HFSS) products by the UK Government [36] was viewed by industry to be misguided as they stated it may stop them being able to advertise their reformulated products; not just those impacted by the SDIL but products voluntarily reformulated which would still be classified as HFSS. Stakeholder requests for consistency across policy areas was also expressed by interviewees regarding this advertising ban [51]. This indicates that a more consistent approach to determining which products government wants industry to change would help ensure policies do not undermine one another and build trust in government amongst industry.

For peer review only

Conclusion

This study explored food and drink industry perspectives on the SDIL. We found that industry accepted that legislation was useful in levelling the commercially competitive playing field. However, in practice participants stated that the SDIL had not created a 'true' level playing field as little consideration had been given to excluded product categories during policy design. Technical aspects of implementation were not adequately included and led to complexity for out of home retailers. Legislation on SSBs needs to take account of all industry sectors it affects, including out of home retail, as well as the manufacturing sector. Participants stated that only targeting sugary soft drinks was unfair due to the progress already made in the category compared to others (e.g., confectionary). The critical role of consumers in creating momentum towards sugar reduction in SSBs prior to the SDIL announcement, as well as dictating response to the SDIL was discussed. It is hypothesised that pro-health public views could be a useful lever in encouraging positive industry action independently of food and drink regulation. The impact of the SDIL was felt beyond the soft drinks industry, driving other product sectors to reformulate in anticipation of future regulation.

For peer review only

Acknowledgements: For the purpose of Open Access, the author has applied a Creative Commons Attribution (CC BY) licence to any Author Accepted Manuscript version arising.

Competing Interests: None

Contributorship Statement: Dr. Catrin P Jones: Conceptualization, Methodology, Formal analysis, Investigation, Data Curation, Writing - Original Draft, Project administration. Dr. Hannah Forde: Formal analysis, Writing - Review & Editing. Dr Tarra L. Penney: Conceptualization, Methodology, Investigation, Data Curation, Writing - Review & Editing, Funding acquisition. Dr. Dolly R. Z. Theis: Formal analysis, Writing - Review & Editing Prof. Steven Cummins: Conceptualization, Methodology, Formal analysis, Writing - Review & Editing, Supervision, Funding acquisition. Prof. Jean Adams: Formal analysis, Writing - Review & Editing, Funding acquisition. Dr. Cherry Law: Formal analysis, Writing - Review & Editing. Prof. Harry Rutter: Formal analysis, Writing - Review & Editing, Funding acquisition. Prof. Richard Smith: Formal analysis, Writing - Review & Editing, Funding

For peer review only - http://bmjopen.bmj.com/site/about/guidelines.xhtml

acquisition. Prof. Martin White: Conceptualization, Methodology, Resources, Supervision, Project administration, Funding acquisition, Formal analysis, Writing - Review & Editing.

Funding: This project was funded by the NIHR Public Health Research programme (Grant Nos. 16/49/01 and 16/130/01). At the time this study was conducted CPJ, MW, ELR, HF, TLP, DT, JA, OA, SA, were also supported in part by: Programme grants to the MRC Epidemiology Unit from the Medical Research Council (grant No. MC_UU_12015/6 and MC_UU_00006/7); and the Centre for Diet and Activity Research (CEDAR), a UKCRC Public Health Research Centre of Excellence – funding from the British Heart Foundation, Cancer Research UK, the Economic and Social Research Council, the Medical Research Council, the National Institute for Health Research, and the Wellcome Trust, under the auspices of the UK Clinical Research Collaboration is gratefully acknowledged. HF received funding for her PhD studentship from the Economic and Social Research Council and Public Health England, and she has received further discretionary funding from the Economic and Social Research Council and Murray Edwards College, Cambridge. The views expressed are those of the authors and not necessarily those of the any of the above-named funders. The

For peer review only

funders had no role in study design, data collection and analysis, decision to publish, or preparation of the manuscript.

Data availability statement. To maintain participant anonymity and confidentiality, data is not available due to the identifiable nature of participants from transcripts and recordings.

Ethics approval statement: The study was approved by the Humanities and Social Science Ethics Committee at the University of Cambridge, UK. No approval ID was provided by this committee.

References

1 Afshin A, Sur PJ, Fay KA, *et al.* Health effects of dietary risks in 195 countries, 1990–2017: a systematic analysis for the Global Burden of Disease Study 2017. *The Lancet* 2019;**393**:1958–72. doi:10.1016/S0140-6736(19)30041-8

2 World Health Organization. Taxes on sugary drinks: Why do it? World Health Organization 2017.

3 Andreyeva T, Marple K, Marinello S, *et al.* Outcomes following taxation of sugar-sweetened beverages: a systematic review and meta-analysis. *JAMA Netw Open* 2022;**5**:e2215276–e2215276.

4 Teng AM, Jones AC, Mizdrak A, *et al.* Impact of sugar-sweetened beverage taxes on purchases and dietary intake: systematic review and meta-analysis. *Obesity Reviews* 2019;**20**:1187–204.

For peer review only - http://bmjopen.bmj.com/site/about/guidelines.xhtml

5 Cawley J, Frisvold D. Taxes on sugar-sweetened beverages: Political economy, and effects on prices, purchases, and consumption. *Food Policy* 2023;**117**:102441.

6 The World Bank. Global SSB Tax Database. https://ssbtax.worldbank.org/ (accessed 25 May 2023).

7 HM Revenue & Customs. Soft Drinks Industry Levy. 2016.https://www.gov.uk/government/publications/soft-drinks-industry-levy/soft-drinks-industry-levy#:~:text=from the levy.-,Policy objective,to reduce the sugar content (accessed 16 Jun 2020).

8 HM Revenue and Customs. Soft Drinks Industry Levy statistics background and references. 2022.https://www.gov.uk/government/statistics/soft-drinks-industry-levy-statistics/soft-drinks-industry-levy-statistics-background-and-references (accessed 10 Nov 2022).

9 Cabinet Office, Department of Health and Social Care, HM Treasury, *et al.* Childhood obesity: a plan for action. 2016.https://www.gov.uk/government/publications/childhood-obesity-a-plan-for-action (accessed 8 Apr 2021).

10 Department of Health and Social Care. Childhood obesity: a plan for action, chapter 2. 2018.

11 HM Revenue & Customs. Guidance: Check if you need to register for the Soft Drinks Industry Levy. 2018.https://www.gov.uk/guidance/check-if-you-need-to-register-for-the-soft-drinks-industry-levy (accessed 21 May 2023).

12 Gauke D. Budget 2016. 2016. https://www.gov.uk/government/publications/budget-2016-documents

13 HM Treasury, Osborne. G. Budget 2016: George Osborne's speech. 2016.https://www.gov.uk/government/speeches/budget-2016-george-osbornes-speech (accessed 6 Apr 2023).

14 Rogers NT, Cummins S, Forde H, *et al.* Associations between trajectories of obesity prevalence in English primary school children and the UK soft drink industry levy: an interrupted time series analysis of surveillance data. *medRxiv* Published Online First: 2022. doi:https://doi.org/10.1101/2022.09.16.22280030

15 Hilton S, Buckton CH, Patterson C, *et al.* Following in the footsteps of tobacco and alcohol? Stakeholder discourse in UK newspaper coverage of the Soft Drinks Industry Levy. *Public Health Nutr* 2019;**22**:2317–28. doi:10.1017/S1368980019000739

For peer review only

16 Economics O. The Economic Impact of the Soft Drinks Levy, Final Report. *London: Oxford Economics* 2016.

17 Penney TL, Adams J, White M. Industry reactions to the UK Soft Drinks Industry Levy: Unpacking the evolving discourse from announcement to implementation. *J Epidemiol Community Health (1978)* 2018;**72**:A43. doi:10.1136/jech-2018-SSMabstracts.88

18 Law C, Cornelsen L, Adams J, *et al.* An analysis of the stock market reaction to the announcements of the UK Soft Drinks Industry Levy. *Econ Hum Biol* 2020;**38**:100834. doi:10.1016/j.ehb.2019.100834

19 Law C, Cornelsen L, Adams J, *et al.* The impact of UK Soft Drinks Industry Levy on manufacturers' domestic turnover. *Econ Hum Biol* 2020;:100866.

20 Penney T, Adams J, White M. LB4 Industry reactions to the UK soft drinks industry levy: unpacking the evolving discourse from announcement to implementation. BMJ 2018. A43.1-A43. doi:10.1136/jech-2018-ssmabstracts.88

21 Hilton S, Buckton CH, Katikireddi SV, *et al.* Who says what about sugar-sweetened beverage tax? Stakeholders' framing of evidence: a newspaper analysis. *The Lancet* 2017;**390**:S44. doi:10.1016/s0140-6736(17)32979-3

22 Buckton CH, Hilton S, Patterson C, *et al.* DID PROPONENTS AND OPPONENTS OF THE SOFT DRINKS INDUSTRY LEVY USE THE NEWS MEDIA TO INFLUENCE THE POLICY DEBATE? A QUALITATIVE DISCOURSE ANALYSIS USING PRACTICAL REASONING. *J Epidemiol Community Health (1978)* 2018;**72**:A1–2. doi:10.1136/jech-2018-SSMabstracts.2

23 Buckton CH, Patterson C, Hyseni L, *et al.* The palatability of sugar-sweetened beverage taxation: A content analysis of newspaper coverage of the UK sugar debate. *PLoS One* 2018;**13**:1–15. doi:10.1371/journal.pone.0207576

24 Forde H, Penney TL, White M, *et al.* Understanding Marketing Responses to a Tax on Sugary Drinks: A Qualitative Interview Study in the United Kingdom, 2019. *Int J Health Policy Manag* Published Online First: 23 February 2022. doi:10.34172/ijhpm.2022.5465

25 Braun V, Clarke V. *Successful Qualitative Research: A Practical Guide for Beginners.* SAGE 2013.

26 White M. Evaluation of the health impacts of the UK Treasury Soft Drinks Industry Levy (SDIL). 2017;:1–46.https://www.journalslibrary.nihr.ac.uk/programmes/phr/1613001/#/

For peer review only

27 Harvey WS. Methodological Approaches for Interviewing Elites. *Geogr Compass* 2010;**4**:193–205. doi:10.1111/j.1749-8198.2009.00313.x

28 Berry JM, Browne W. Validity and Reliability Issues In Elite Interviewing. *PS Polit Sci Polit* 2003;**35**:679–82.

29 Harvey WS. Strategies for conducting elite interviews. *Qualitative Research* 2011;**11**:431–41. doi:10.1177/1468794111404329

30 Goldstein K. Getting in the Door: Sampling and Completing Elite Interviews. *Political Science & Politics* 2002;**35**:669–72. doi:10.1017/S1049096502001130

31 Stephens N. Collecting data from elites and ultra elites: Telephone and face-to-face interviews with macroeconomists. *Qualitative Research* 2007;**7**:203–16. doi:10.1177/1468794107076020

32 Braun V, Clarke V. Using thematic analysis in psychology. *Qual Res Psychol* 2006;**3**:77–101.

33 Braun V, Clarke V. Can I use TA? Should I use TA? Should I not use TA? Comparing reflexive thematic analysis and other pattern-based qualitative analytic approaches. *Couns Psychother Res* 2020;:37–47. doi:10.1002/capr.12360

34 Braun V, Clarke V. What can 'thematic analysis' offer health and wellbeing researchers? *Int J Qual Stud Health Well-being* 2014;**9**. doi:10.3402/qhw.v9.26152

35 Department of Health and Social Care. Calorie labelling in the out of home sector: implementation guidance. 2021.https://www.gov.uk/government/publications/calorie-labelling-in-the-out-of-home-sector/calorie-labelling-in-the-out-of-home-sector-implementation-guidance#:~:text=The%20out%20of%20home%20sector%20is%20generally%20considered%20to%20be,on%20or%20off%20the%20premises (accessed 14 Dec 2022).

36 Department for Digital Culture Media & Sport, Department of Health & Social Care. Introducing a total online advertising restriction for products high in fat, sugar and salt (HFSS). 2021.https://www.gov.uk/government/consultations/total-restriction-of-online-advertising-for-products-high-in-fat-sugar-and-salt-hfss/introducing-a-total-online-advertising-restriction-for-products-high-in-fat-sugar-and-salt-hfss (accessed 6 Jan 2023).

37 Office for Health Improvement and Disparities. Sugar, salt and calorie reduction and reformulation. 2017.https://www.gov.uk/government/collections/sugar-reduction (accessed 11 Jan 2023).

For peer review only

38 Stewart H. ‘Sin taxes’: Boris Johnson vows to review sugar levy. The Guardian. 2019.https://www.theguardian.com/politics/2019/jul/03/boris-johnson-vows-to-review-whether-sugar-tax-improves-health (accessed 19 Oct 2022).

39 Forde H, Penney T, White M, *et al.* A Framework for Understanding Marketing Responses to a Tax on Sugary Drinks: a Qualitative Interview Study in the United Kingdom, 2019. *Int J Health Policy Manag* 2022.

40 Lacy-Nichols J, Marten R, Crosbie E, *et al.* The public health playbook: ideas for challenging the corporate playbook. *Lancet Glob Health* 2022.

41 Wiist WH. The corporate play book, health, and democracy: the snack food and beverage industry’s tactics in context. *Sick societies: Responding to the global challenge of chronic disease* 2011;:204–16.

42 Petticrew M, Katikireddi SV, Knai C, *et al.* ‘Nothing can be done until everything is done’: The use of complexity arguments by food, beverage, alcohol and gambling industries. *J Epidemiol Community Health (1978)* 2017;**71**:1078–83. doi:10.1136/jech-2017-209710

43 Theis D, White M. Is Obesity Policy in England Fit for Purpose? Analysis of Government Strategies and Policies, 1992–2020. *Milbank Quarterly* 2021;**99**:126–70. doi:10.1111/1468-0009.12498

44 Forde H, Penney T, White M, *et al.* Understanding Marketing Responses to a Tax on Sugary Drinks: a Qualitative Interview Study in the United Kingdom, 2019. *Int J Health Policy Manag* 2022.

45 Scarborough P, Adhikari V, Harrington RA, *et al.* Impact of the announcement and implementation of the UK Soft Drinks Industry Levy on sugar content, price, product size and number of available soft drinks in the UK, 2015-19: A controlled interrupted time series analysis. *PLoS Med* 2020;**17**:e1003025.

46 Boseley S. Jamie Oliver’s Sugar Rush: a crusade to save Britain’s health. The Guardian. 2015.

47 Tedstone A, Targett V, Allen R. Sugar Reduction: The evidence for action About Public Health England. *Public Health England* Published Online First: 2015.www.gov.uk/phe%5Cnwww.facebook.com/PublicHealthEngland

48 World Health Organization. Sugars intake for adults and children. 2015. https://www.who.int/publications/i/item/9789241549028 (accessed 14 Oct 2022).

49 Nieburg O. Kellogg shuns ‘silver bullet’ 30% UK cereals sugar cap. bakeryandsnacks.com.

For peer review only - http://bmjopen.bmj.com/site/about/guidelines.xhtml

2013.https://www.bakeryandsnacks.com/Article/2013/01/07/Kellogg-shuns-30-sugar-cap-proposal-for-UK-cereals (accessed 6 Jan 2023).

50 Rose D, Theis Z. What influences government policymaking? The case of childhood obesity in England. 2022.

51 Forde H, Boyland EJ, Scarborough P, *et al.* Exploring the potential impact of the proposed UK TV and online food advertising regulations: a concept mapping study. *BMJ Open* 2022;**12**. doi:10.1136/bmjopen-2021-060302

For peer review only

For peer review only - http://bmjopen.bmj.com/site/about/guidelines.xhtml

Supplementary File 1: Researcher Reflexivity

Braun and Clarke discuss reflexivity as a fundamental characteristic of thematic analysis, involving critical reflection of researcher perspectives, and how these will be integrated within the analysis and interpretation of data [22]. The complete elimination of bias is not something that can be achieved in qualitative research and more importantly should not be an aim. Unlike statistical analysis, the researcher is the tool of analysis. The researcher therefore is an integral part of the analytic process and to conduct qualitative thematic analysis well, they must develop an understanding or how their own perspectives, position and view of reality helps illuminate will influence the analysis [22].

This study aimed to centre participants' words in a descriptive manner to preserve their intention. This approach was also chosen due to reflection by the research team on our positionality as public health academics. We aimed to understand industry perspectives regarding the SDIL; however, it is important to acknowledge that the personal and professional goals of the research team (authors on this paper) as public health researchers are likely to be different from those of people working in the food and drink industry. Therefore, a descriptive approach was selected which prizes participants' words and perspectives over and above researcher interpretations. Whilst our perspectives have still influenced the analysis, as they should in good qualitative practice, we sought to minimise the influence of our biases and negativity towards some of the practices of the food and drink industry, to truly 'listen' to the perspectives of our participants. As a result, a modified version of Braun and Clarke's thematic analysis was used; reflexivity was a priority throughout the analysis in line with the approach however we sought to be less interpretive than their more recent guidance proposes [22].

For peer review only

It is also important to note that, although we have taken a descriptive approach, the results represent participant perspectives. Whilst the researchers work to put aside their biases which may lean towards those more critical of the food and drink industry; statements, findings and themes found do not represent an objective truth, rather the reported perspectives of participants. We urge readers of this work to use their own critical reflection when interpreting and using these findings.

For peer review only

COREQ (COnsolidated criteria for REporting Qualitative research) Checklist

A checklist of items that should be included in reports of qualitative research. You must report the page number in your manuscript where you consider each of the items listed in this checklist. If you have not included this information, either revise your manuscript accordingly before submitting or note N/A.

Topic	Item No.	Guide Questions/Description	Reported on Page No.
**Domain 1: Research team and reflexivity**			
Personal characteristics			
Interviewer/facilitator	1	Which author/s conducted the interview or focus group?	
Credentials	2	What were the researcher's credentials? E.g. PhD, MD	
Occupation	3	What was their occupation at the time of the study?	
Gender	4	Was the researcher male or female?	
Experience and training	5	What experience or training did the researcher have?	
Relationship with participants			
Relationship established	6	Was a relationship established prior to study commencement?	
Participant knowledge of the interviewer	7	What did the participants know about the researcher? e.g. personal goals, reasons for doing the research	
Interviewer characteristics	8	What characteristics were reported about the inter viewer/facilitator? e.g. Bias, assumptions, reasons and interests in the research topic	
**Domain 2: Study design**			
Theoretical framework			
Methodological orientation and Theory	9	What methodological orientation was stated to underpin the study? e.g. grounded theory, discourse analysis, ethnography, phenomenology, content analysis	
Participant selection			
Sampling	10	How were participants selected? e.g. purposive, convenience, consecutive, snowball	
Method of approach	11	How were participants approached? e.g. face-to-face, telephone, mail, email	
Sample size	12	How many participants were in the study?	
Non-participation	13	How many people refused to participate or dropped out? Reasons?	
Setting			
Setting of data collection	14	Where was the data collected? e.g. home, clinic, workplace	
Presence of non-participants	15	Was anyone else present besides the participants and researchers?	
Description of sample	16	What are the important characteristics of the sample? e.g. demographic data, date	
Data collection			
Interview guide	17	Were questions, prompts, guides provided by the authors? Was it pilot tested?	
Repeat interviews	18	Were repeat inter views carried out? If yes, how many?	
Audio/visual recording	19	Did the research use audio or visual recording to collect the data?	
Field notes	20	Were field notes made during and/or after the inter view or focus group?	
Duration	21	What was the duration of the inter views or focus group?	
Data saturation	22	Was data saturation discussed?	
Transcripts returned	23	Were transcripts returned to participants for comment and/or	

For peer review only

Topic	Item No.	Guide Questions/Description	Reported on Page No.
		correction?	
**Domain 3: analysis and findings**			
Data analysis			
Number of data coders	24	How many data coders coded the data?	
Description of the coding tree	25	Did authors provide a description of the coding tree?	
Derivation of themes	26	Were themes identified in advance or derived from the data?	
Software	27	What software, if applicable, was used to manage the data?	
Participant checking	28	Did participants provide feedback on the findings?	
Reporting			
Quotations presented	29	Were participant quotations presented to illustrate the themes/findings? Was each quotation identified? e.g. participant number	
Data and findings consistent	30	Was there consistency between the data presented and the findings?	
Clarity of major themes	31	Were major themes clearly presented in the findings?	
Clarity of minor themes	32	Is there a description of diverse cases or discussion of minor themes?	

Developed from: Tong A, Sainsbury P, Craig J. Consolidated criteria for reporting qualitative research (COREQ): a 32-item checklist for interviews and focus groups. *International Journal for Quality in Health Care*. 2007. Volume 19, Number 6: pp. 349 – 357

Once you have completed this checklist, please save a copy and upload it as part of your submission. DO NOT include this checklist as part of the main manuscript document. It must be uploaded as a separate file.

For peer review only

BMJ Open

Industry views of the UK Soft Drinks Industry Levy: a thematic analysis of elite interviews with food and drink industry professionals, 2018-20

Journal:	*BMJ Open*
Manuscript ID	bmjopen-2023-072223.R3
Article Type:	Original research
Date Submitted by the Author:	14-Jul-2023
Complete List of Authors:	Jones, Catrin; University of Cambridge, MRC Epidemiology Unit<br>Forde, Hannah; University of Cambridge, MRC Epidemiology Unit; University of Oxford, Nuffield Department of Primary Care Health Sciences<br>Penney, Tarra; York University, Global Food System and Policy Research, School of Global Health, Faculty of Health<br>Theis, Dolly; University of Cambridge, MRC Epidemiology Unit<br>Cummins, Steven; London School of Hygiene & Tropical Medicine, Population Health Innovation Lab, Department of Public Health, Environments & Society, Faculty of Public Health and Policy<br>Adams, J; University of Cambridge, MRC Epidemiology Unit<br>Law, Cherry; University of Reading, Department of Agri-Food Economics and Marketing<br>Rutter, Harry; University of Bath Department of Social and Policy Sciences, Social and Policy Sciences<br>Smith, Richard ; University of Exeter, Faculty of Health and Life Sciences<br>White, Martin; University of Cambridge, MRC Epidemiology Unit
**Primary Subject Heading**:	Health policy
Secondary Subject Heading:	Qualitative research
Keywords:	Health policy < HEALTH SERVICES ADMINISTRATION & MANAGEMENT, Public health < INFECTIOUS DISEASES, QUALITATIVE RESEARCH

SCHOLARONE™
Manuscripts

I, the Submitting Author has the right to grant and does grant on behalf of all authors of the Work (as defined in the below author licence), an exclusive licence and/or a non-exclusive licence for contributions from authors who are: i) UK Crown employees; ii) where BMJ has agreed a CC-BY licence shall apply, and/or iii) in accordance with the terms applicable for US Federal Government officers or employees acting as part of their official duties; on a worldwide, perpetual, irrevocable, royalty-free basis to BMJ Publishing Group Ltd ("BMJ") its licensees and where the relevant Journal is co-owned by BMJ to the co-owners of the Journal, to publish the Work in this journal and any other BMJ products and to exploit all rights, as set out in our licence.

The Submitting Author accepts and understands that any supply made under these terms is made by BMJ to the Submitting Author unless you are acting as an employee on behalf of your employer or a postgraduate student of an affiliated institution which is paying any applicable article publishing charge ("APC") for Open Access articles. Where the Submitting Author wishes to make the Work available on an Open Access basis (and intends to pay the relevant APC), the terms of reuse of such Open Access shall be governed by a Creative Commons licence – details of these licences and which Creative Commons licence will apply to this Work are set out in our licence referred to above.

Other than as permitted in any relevant BMJ Author's Self Archiving Policies, I confirm this Work has not been accepted for publication elsewhere, is not being considered for publication elsewhere and does not duplicate material already published. I confirm all authors consent to publication of this Work and authorise the granting of this licence.

For peer review only

Industry views of the UK Soft Drinks Industry Levy: a thematic analysis of elite interviews with food and drink industry professionals, 2018-20

Dr Catrin P Jones[a*], Dr Hannah Forde [a,<], Dr Tarra L Penney[b], Dr Dolly Theis [a], Prof Steven Cummins[c], Prof Jean Adams [a], Dr Cherry Law[d], Prof Harry Rutter[e], Prof Rich Smith[f], Prof Martin White[a].

a MRC Epidemiology Unit, School of Clinical Medicine, University of Cambridge, United Kingdom

b Global Food System and Policy Research, School of Global Health, Faculty of Health, York University, Canada

c Population Health Innovation Lab, Department of Public Health, Environments & Society, Faculty of Public Health and Policy, London School of Hygiene & Tropical Medicine, United Kingdom

d Department of Agri-Food Economics and Marketing, University of Reading, Reading, UK

e Department of Social and Policy Sciences, University of Bath, Bath, UK

f Faculty of Health and Life Sciences, University of Exeter, Exeter, UK

For peer review only - http://bmjopen.bmj.com/site/about/guidelines.xhtml
For peer review only

< Present address: Nuffield Department of Primary Care Health Sciences, University of Oxford, Oxford, United Kingdom

***Correspondence to:**

Dr Catrin Pedder Jones

MRC Epidemiology Unit, University of Cambridge School of Clinical Medicine, Box 285 Institute of Metabolic Science, Cambridge Biomedical Campus, Cambridge, CB2 0QQ, United Kingdom

catrin.jones@mrc-epid.cam.ac.uk

+44 (0)1223 769 114

Keywords: Soft drinks, Soft drinks industry levy, Food and drink industry, sugar sweetened beverage

Word count: *6605*

Abstract

For peer review only - http://bmjopen.bmj.com/site/about/guidelines.xhtml

Objectives: The UK Soft Drinks Industry Levy (SDIL), implemented in 2018, has been successful in reducing the sugar content, and purchasing, of soft drinks, with limited financial impact on industry. Understanding the views of food and drink industry professionals involved in reacting to the SDIL is important for policymaking. However, their perceptions of the challenges of implementation and strategic responses are unknown. The aim of this study therefore was to explore how senior food and drink industry professionals viewed the SDIL.

Design: We undertook a qualitative descriptive study using elite interviews. Data were analysed using Braun and Clarke's approach to thematic analysis, taking an inductive exploratory and descriptive approach not informed by prior theory or frameworks.

Setting and participants: Interviews were conducted via telephone with 14 senior professionals working in the food and drink industry.

Results: Five main themes were identified: *(1) A level playing field…for some*; Industry accepted the SDIL as an attempt to create a level playing field but due to the exclusion of milk-based drinks, this was viewed as inadequate. *(2) Complex to implement, but no lasting negatives*; The SDIL was complex, expensive and time consuming to implement, with industry responses dependent on leadership buy-in. "(3) *Why us? – the SDIL unfairly targets*

For peer review only - http://bmjopen.bmj.com/site/about/guidelines.xhtml
For peer review only

the drinks industry"; soft drinks are an unfair target when other categories also contain high sugar. "(4) *The consumer is king";* Consumers were a key focus of the industry response to this policy. "(5) *The future of the SDIL*"; There appeared to be a wider ripple effect, which primed industry to prepare for future regulation in support of health and environmental sustainability.

Conclusions: Insights from senior food and drink industry professionals illustrate how sugar sweetened beverage taxes might be successfully implemented and improve understanding of industry responses to taxes and other food and drink policies.

Strengths and limitations of this study

- This qualitative study explored how senior food and drink industry professionals viewed the Soft Drinks Industry Levy.
- We undertook elite interviews with 14 professionals working in the food and drink industry, who have often been difficult to recruit in other studies.
- Braun and Clarke's thematic analysis, taking a descriptive approach, was used to analyse the data.

For peer review only

- Elite interviewing methods allow for the building of relationships to elicit meaningful responses from participants.
- A limitation of this work is that interviews were carried out over a long period of time due to challenges in recruitment.

For peer review only

For peer review only - http://bmjopen.bmj.com/site/about/guidelines.xhtml

Introduction

Diet-related non-communicable diseases are a major and growing problem, responsible for over 11 million deaths globally each year [1]. Sugar consumption is of particular concern, with the World Health Organization (WHO) recommending member states introduce sugar-sweetened beverage (SSB) taxes [2]. Reviews suggest that they reduce sales of, increases prices of and encourage reformulation of SSBs [3–5] and over 100 SSB taxes have been implemented worldwide covering 52% of the world's population [6]. SSB taxes have a variety of designs with 87% excise taxes [6]. The WHO recommend that a tiered SSB tax be introduced in companies with high administrative capacity, similar to that which has been introduced in the UK [2]. The Soft Drinks Industry Levy (SDIL) was announced on 16th March 2016 and implemented in the United Kingdom (UK) on 6th April 2018. According to the budget speech by George Osborne, Chancellor of the Exchequer at the time, it was designed to incentivise manufacturers of SSBs to reformulate their products [7] via charging a levy on soft drinks produced by companies when they leave the warehouse or when imported into the country [8]. Integrated in August 2016 as part of the UK Government's Childhood Obesity: A Plan For Action [9], the SDIL consists of two tiers (for particulars of the tax, see Box 1). A public consultation on the proposals between August - October 2016

For peer review only

set out the plans for the tiers and exclusions as described in Box 1. Few changes were made as a result of this consultation and the SDIL was given royal assent on 27th April 2017. The Government published a second chapter of its childhood obesity plan in 2018, which suggested the SDIL may be extended to milk-based drinks, though this has not yet occurred [10].

For peer review only

Box 1. Soft Drinks Industry Levy Particulars [7]

Eligible drinks:

- ≥8 g total sugar per 100 mL charged at 24 pence per litre
- ≥5 g and <8 g total sugar per 100 mL charged at 18 pence per litre

Exemptions:

- Drinks containing more than 75% milk or 1.2% alcohol
- Alcohol substitute drinks
- Powdered drinks

For peer review only - http://bmjopen.bmj.com/site/about/guidelines.xhtml

- 100% fruit juices
- Manufacturers selling under one million litres of drinks per year[11]

The SDIL was one of the first SSB fiscal interventions explicitly designed to incentivise reformulation [7,12,13]. This aim was largely achieved, substantially reducing overall SSB sugar content, and inducing a major shift of drinks from the higher levy tier to the lower tier and untaxed bracket between 2016 and 2018 (Scarborough et al., 2020). Reformulation is reflected in purchases of sugar from SSBs, with [14]. Prior to implementation of the SDIL, the food and drinks industry (hereafter referred to as 'industry') viewed the SDIL as having a potentially negative impact on profits resulting in job losses [15–17]. A negative stock market reaction to the SDIL announcement was observed, but this only lasted two days [18]. Similarly, a negative impact on company domestic turnover was observed following the announcement of the SDIL, but this resolved by the time of its implementation [19].

Critical to the success of the SDIL is the implementation of and reaction to the regulations by the drink industry. Therefore, it is important to understand perspectives of the industry as well as those who work in it regarding the implementation of such taxes. Previous work has

For peer review only

investigated industry perspectives of the SDIL expressed through the news media [15,20–23] and the views of industry, civil society and academic participants on how marketing changed in response to the SDIL [24]. A notable gap in the literature, however, is perspectives of the SDIL from the commercial sector, not communicated publicly through news media nor focused solely on marketing responses. Important learning can be obtained by exploring the perspectives of commercial actors involved in responding to regulation. Interviews with senior members of industry can help examine the impact of the SDIL on both the soft drinks industry and wider food and drink industry, an avenue not previously explored. This study therefore aimed to address these knowledge gaps and inform policymaking by exploring the perspectives of senior industry professionals regarding the UK SDIL.

Methods

Study design

This study adopted a qualitative descriptive design involving elite interviews with senior industry professionals.

Methodological orientation

For peer review only - http://bmjopen.bmj.com/site/about/guidelines.xhtml

This research took an experiential qualitative approach, within a critical realist position. Participant perspectives and perceptions were prized over researcher interpretations, and reality was derived from our participants' words and meaning, rather than a reality constructed through researchers' interpretation of their words [25]. A descriptive approach was used to explore how the SDIL was viewed from the position of our participants.

Research team

Prof Martin White (MW), Prof Steven Cummins (SC), Prof Jean Adams (JA), Prof Rich Smith (RS) and Prof Harry Rutter (HR) secured funding for the overall evaluation of the SDIL within which this study formed a part [26]. Interviews were conducted by Postdoctoral Research Associates Dr. Tarra L Penney (TLP) and Dr. Catrin P Jones (CPJ). TLP led the design of data collection and CPJ led the design of the analysis. MW & SC provided guidance on the design of both elements. TLP and CPJ recruited and interviewed participants. CPJ led the analysis with support from Dr Hannah Forde (HF). HF conducted secondary coding to support theme generation and interpretation. All authors previously mentioned, as well as Dr Dolly Theis (DT) and Dr Cherry Law (CL) were involved in data analysis and interpretation, as well as drafting this manuscript.

For peer review only

Participants

Senior professionals from the soft drinks, food and other drinks industries were recruited to this study using purposive and snowball sampling. We adopted 'elite interviewing' methods to maximise involvement of senior professionals in positions of influence within their organisation and with high levels of responsibility [27]. This technique provides a series of strategies to support recruitment of difficult to access key participants, and to ensure the validity and reliability of data [28]. The principles of elite interviewing were used to inform recruitment including stronger emphasis on the maintenance of trust, importance of interview tone of the interview, preparing appropriately, and engaging in and tailoring dialogue relevant to each informant, more so than in traditional interviews [29,30].

Individuals were considered eligible to participate based on the following criteria: a) currently or previously held a high-level industry position (at the managerial, director or chief officer level), b) their organisation and their professional role was directly or indirectly impacted by the SDIL and c) they could provide a novel perspective, determined by their job role or the company they work for not previously heard in our interviews, to ensure a range

For peer review only - http://bmjopen.bmj.com/site/about/guidelines.xhtml

of views. Recruitment typically involved an email introduction by a member of the team or informant contact, although CPJ also attended industry food events and recruited face to face. Initial contact was followed by an informal telephone conversation with TLP or CPJ to discuss the research purpose, team and informant interests and perspectives, ultimately proceeding to full participation via telephone interview. Recruitment ceased when networks were exhausted, and no further contacts identified.

Data collection

Telephone interviews were conducted from June 2018 to June 2020. Participant information sheets were sent to potential participants prior to participating in the informal discussion. Informed consent was obtained verbally prior to commencement of the formal telephone interview, which was digitally audio-recorded. Interviews were undertaken using a minimally structured topic guide containing three broad areas of inquiry: a) Can you tell me about your role and organization? b) Can you tell me about your sector as a whole? c) What do you know about the UK Soft Drinks Industry Levy and its impacts? Elite interviewing necessitates informed and adaptive dialogue [29,31], meaning participants could engage in ways most relevant to their specific expertise or experiences within these broad areas.

For peer review only - http://bmjopen.bmj.com/site/about/guidelines.xhtml

Interviews were transcribed verbatim by a trusted external company, and transcripts were checked against the audio files by CPJ to identify any inaccuracies. Transcripts were anonymised prior to analysis by removing names of people, organisations, and brands.

Analysis

Analysis commenced once all interviews had been conducted and transcribed. Braun and Clarke's thematic analysis was used, taking an inductive exploratory and descriptive approach not informed by any prior theory or framework [32]. This approach is flexible due to lack of alignment with specific epistemological and ontological stance [33,34]. Six analytic steps were conducted: 1) familiarisation, 2) data coding, 3) initial theme generation, 4) theme development and review, 5) theme refining, defining and naming, 6) writing up.

CPJ listened to audio files and read transcripts at least twice to become familiar with them, whilst making notes on initial impressions and patterns (step 1). Following familiarisation, CPJ worked systematically through the entire data set and conducted complete coding of all data, in which segments of data were given a label to describe their area of interest. Coding was supported by NVivo software version 12. Semantic codes were derived directly from

For peer review only - http://bmjopen.bmj.com/site/about/guidelines.xhtml

participants' speech or codes where phrases of speech were brief enough to be directly coded (step 2). CPJ then sorted these initial codes into concise categories (overarching codes), which clearly described the content of the data (step 3). A reflective diary was kept throughout the coding process by CPJ to note reflections on findings and to ensure a data-driven analytic process. Please see supplementary file 1 for a detailed account of reflexivity.

HF also familiarised themself with the transcripts (step 1) and then examined CPJ's coding to ensure the codes were data driven with as little interpretation as possible (step 2). CPJ then collated codes that shared a common pattern into themes (step 3). Again, CPJ and HF met to discuss and refine the themes to ensure they were descriptive with minimal interpretation (step 4).

A document containing themes, codes within them, and extensive anonymised quotes was shared with all co-authors in two phases: phase 1 March 2022 and phase 2 October 2022 (step 5). This data clinic aimed to minimise researcher interpretation. A document presented theme descriptions and asked co-authors to answer the following questions for each theme: 1) Is the theme descriptive? 2) Does the theme represent the data accurately? 3) What do you

For peer review only

think the theme tells us about the SDIL from the perspective of industry? JA, DT, CL completed the data clinic document in phase 1. Themes were amended based on their reflections and the document updated in October 2022. SC, MW, HR & RS completed the data clinic form in phase 2. Final themes and the manuscript were written up by CPJ and reviewed by all co-authors (step 6).

Patient and public involvement

This study is part of the 'Evaluation of the health impacts of the UK Treasury Soft Drinks Industry Levy (SDIL)' funded by NIHR (award no. 16/130/01). Project oversight is provided by an independent study steering committee (ISSC) which contains members of the public. The ISSC for the overall project met biannually from 2017 – 2023 and were asked to provide advice on methodology as well as interpretation of our findings.

Results

Fourteen participants were recruited (Table 1). Participants' roles within organisations were diverse; chief officers, directors, and managers with overall responsibility or with specialist responsibilities for finance, strategy, operations, marketing, public relations or nutrition.

For peer review only - http://bmjopen.bmj.com/site/about/guidelines.xhtml

Interviews ranged in length from 26 to 62 minutes. Six additional participants were approached and took part in informal discussions; three did not participate due to scheduling issues, and three refused to take part. Five inductively derived, interlinked themes and 15 subthemes were identified (Box 2).

For peer review only

Table 1. Participant details

Sector Category	N
Drink manufacturers	4
Food and drink manufacturers	3
Supermarkets	3
Industry associations	1
Out-of-home* food and drink manufacturers	1
Out-of-home retailers	1
Advertising consultants	1

*** "The out-of-home sector is generally considered to be any outlet where food or drink is prepared in a way that means it is ready for immediate consumption, on or off the premises"* [35]

For peer review only - http://bmjopen.bmj.com/site/about/guidelines.xhtml

Box 2. Theme and sub-theme summary

Theme 1: A level playing field…for some

The SDIL created a level playing field

Milk-based drinks increased the complexity in the out-of-home sector

Challenges for supermarkets with large product portfolios

Theme 2: Complex to implement but no lasting negative effects

Complexities in strategic response – price and product are key

Global companies and internal systems

Contradictory government messaging

Leadership buy-in dictates strategic response

Few long-lasting negative effects & SDIL provided opportunities

Theme 3: Why us? – The SDIL unfairly targets the drinks industry

Sugary drinks in isolation were unfair targets for regulation

Distrust of government's motivations to introduce the SDIL

Theme 4: Consumer is king

Consumer response to product changes resulting from the SDIL

Consumer momentum towards healthier products

Theme 5: The future of the SDIL

Extending to milk and fruit-based drinks

Impact on the wider food and drink industry and on other sectors

Proposal to reverse the SDIL

Theme 1: A level playing field…for some

The SDIL created a level playing field

For peer review only

Industry professionals accepted that the SDIL helped create a level playing field, where no organisation lost out by taking action on health that their competitors did not *"… legislation level playing fields is so important and that's why with these big public health initiatives…I'm actually really quite pro government intervention" – Supermarket.* Soft drinks manufacturers also discussed that the two years to prepare for the implementation of the SDIL was sufficient and they were happy they could develop an adequate response within that time. *"I'm not aware of any significant implementation or challenges that our members have encountered, I mean they did have time to adapt, the legislation was published in good time to allow them to understand exactly what they would be required to do" – Trade association*

However, participants also stated that a lack of understanding and consultation from government meant a 'true' level playing field for all sectors involved in the sales of sugary drinks had not been not achieved *"…you want to really do it smartly so everybody feels they're 100% equally affected and you don't get this… 'my product is in scope, your product is out of scope'…it doesn't create the sense of unilateral 'let's do this'…which is what it should be, if that makes sense" – Food and drink manufacturer.* The lack of consultation by

For peer review only - http://bmjopen.bmj.com/site/about/guidelines.xhtml

the government with sectors who were not soft drinks manufacturers (for example out of home retailers) and the exclusion of milk-based sugary drinks led to this perception *"…milk-based drinks often carried bigger serving sizes and had more total sugar in them than any of our products would. They were excluded from the levy as well which looked like a big shortcoming." Drink manufacturer.*

Milk-based drinks increased the complexity in the out-of-home sector

Interviewees explained that, from their perspective, the government did not think clearly about the technical implications for retailers and out-of-home sector and that it was easier for soft drink manufacturers to respond to the levy than it was for other industry actors *"…I don't think they understood the ways of working and the preparation methods in the out-of-home sector…" – Out-of-home food and drink manufacturer.* A high level of complexity within the out-of-home sector to manufacture and produce drinks for immediate consumption led to higher implementation costs; specifically, the exclusion of milk-based drinks and specification around eligibility of drinks mixed with carbon dioxide, water and ice, and those with and without milk. *"…they were looking at the likes of drinks fountains for carbonated soft drinks because… a bag and box syrup, they would be mixed with ice or carbon dioxide*

For peer review only - http://bmjopen.bmj.com/site/about/guidelines.xhtml

to give the carbonation or either they could be mixed with water and that would capture those drinks in the out-of-home sector, but there was a vagueness to milk-based drinks" – Out-of-home food and drink manufacturer. Some queries to Her Majesty's Revenue and Customs (HMRC – the tax collecting authority in the UK) went unanswered, thus, the out-of-home sector had to interpret the legislation themselves and apply the SDIL according to their interpretation. Representatives of the out-of-home sector did not perceive two years as enough time to have prepared due to confusion surrounding eligibility. In contrast, soft drinks manufacturers stated they had had time to prepare.

Challenges for supermarkets with large product portfolios

Supermarkets felt disadvantaged compared to soft drink manufacturers by the complexities of their sector. They highlighted sector-specific challenges to adapting to the SDIL, including that their product portfolio not only contains branded drinks, about which they have to make decisions, but also private label (own brand) drinks *"…what branded suppliers chose to do was their choice…different brands choosing to reformulate, resize or inflate, which I think led to a fair bit of customer confusion as to what the hell was going on. " – Supermarket.* It was described as challenging and time consuming to manage such a large portfolio and make

For peer review only - http://bmjopen.bmj.com/site/about/guidelines.xhtml

decisions on each product. Particularly as reformulation decisions and portion size reduction reportedly differed between brands yet had to be merchandised together within stores. Retailers also felt that they were disadvantaged as their customers expressed confusion at differing responses by different brands – e.g. 'sugary' drinks reformulated to just below the SDIL threshold but containing both sugar and sweeteners confused customers, with queries directed at retailers rather than drinks manufacturers *"… we tried to make it as clear for customers by putting on all the [shelves] sugar levy applied, so they could very much see…But…when they see a sugar line that's not [included in the SDIL], that's when the questions start coming." – Supermarket.*

Theme 2: Complex to implement but no lasting negative effects

Complexities in strategic response – price and product are key

Industry responded to the SDIL by reviewing product portfolios and strategically selecting responses at the individual product level. This portfolio review approach is why responses differed between companies and between products. Research and development (R&D) and consumer testing were costly for industry during this process, and, linking to theme 1, there were increased costs for those companies with larger product portfolios (e.g. supermarkets).

For peer review only - http://bmjopen.bmj.com/site/about/guidelines.xhtml
For peer review only

For the out-of-home sector, additional complications were noted due to confusion over eligibility of some milk-based drinks *"…government is very keen to always say "oh just reformulate, it will be easy" but it's not easy. It actually takes a lot of time and investment." – Drink manufacturer.*

Consumer testing was vital during the reformulation and decision-making process and consumer preference dictated the strategy taken *"… we invested a significant amount of money…in developing lots and lots of different formulations with lower sugar to see and testing them with consumers in Great Britain to see whether those recipes…would be acceptable to consumers." – Drink manufacturer.* An additional challenge in reformulating drinks described by manufacturers was that sugar serves a functional purpose, in the mouthfeel of drinks mixed with ice and to prevent 'brain freeze', as well as to provide sweetness *"Because, actually, yes, we could stick sweeteners in everything, but, actually, sugar also has like a functional role" – Out-of-home retailer.*

Packaging, merchandising and placement were challenges to overcome, particularly for supermarkets. Decisions were made on own brand products but also on how to retail other

For peer review only - http://bmjopen.bmj.com/site/about/guidelines.xhtml

branded products with different responses to the SDIL (e.g. reformulated drinks, reduced and increased portion sizes, rebranding) *"…there were a number of products that didn't reformulate but did drop size. So, again, there's just small considerations in that around how you merchandise it… So what sounds like a relatively simple change, of dropping from 330ml to, I don't know, 250ml, in reality kind of that complexity flows back through the value chain" – Supermarket.*

Leadership buy-in dictates strategic response

Leadership buy-in to health, where senior management 'buy-in' to the idea that their company should be making pro-health decisions, was discussed as vital in dictating the strategic response to the SDIL *"… I think such a review requires strong leadership and … our COO was very clear that we needed to step in and we needed to do, you know, do the responsible, brave thing." -Drink manufacturer.* Participants described this buy-in as making the process simpler and a lack of buy-in as a barrier to making timely progress *"… having that strong leadership and, you know, complete buy-in from the top team and actually pretty much all the other levels of the organisation, then it's actually quite simple" – Drink manufacturer.*

For peer review only - http://bmjopen.bmj.com/site/about/guidelines.xhtml

Global companies and internal systems

The cost of setting up internal systems to account for and pay the SDIL was expensive, due to the requirement to report to HMRC, regardless of whether or not a company involved in the manufacture or selling of soft drinks was liable to pay the levy *"…It's ridiculous that, you know, it's cost us half a million pounds just to tell Treasury that actually we don't need to pay it." – Drink manufacturer.* The global nature of many of these companies was an additional challenge. Response strategies appropriate for a UK market may not be transferable to other countries, for example reformulation recipes vary due to differences in consumer palate and storage temperatures/facilities *"…that's [computer system] for the UK, and then Ireland have a separate system, France have a separate system, Mexico have a separate system. " – Food and drink manufacturer.*

Contradictory government messaging

There was confusion over whether manufacturers needed to pass on price increases to change consumer behaviour due to contradictory government messaging over the aim of the SDIL. Participants indicated that they thought price increases should have been passed on to target

For peer review only

individual behaviour change; however, manufacturers stated they had no control over whether this occurred as retailers set the price for consumers *"…[the] government had slightly mixed messages so it was pretty clear from the Department of Health and PHE* [Public Health England] *… that they expected to see prices passed on … I think the Treasury were trying to say, oh soft drinks manufacturers don't have to pass this on... Well, apart from the fact that most businesses won't absorb a cost if they can avoid it for obvious reasons, it was the opposite of what the Health Department and others wanted…" – Drink manufacturer.*

Few long-lasting negative effects & SDIL provided opportunities

Participants acknowledged that the SDIL did achieve its aim in stimulating product reformulation to avoid the levy. Although implementation was complex and costly, as previously illustrated, there were few long-lasting negative effects. Some participants suggested the SDIL provided opportunities *"I think some of them would have switched back but we've gained new consumers as well which is, you know, how we, which through sampling and advertising essentially." – Drink manufacturer*. However, participants were sceptical that the SDIL would achieve intended reductions in childhood obesity in the UK.

For peer review only - http://bmjopen.bmj.com/site/about/guidelines.xhtml

"… why [the SDIL] it was thought that that would be a, that policy in isolation would be sufficient to reduce obesity rates." – Drink manufacturer.

Theme 3: Why us? – The SDIL unfairly targets the drinks industry

Sugary drinks in isolation were unfair targets for regulation

Participants felt that the SDIL unfairly targets the soft drinks industry. Participants expressed their frustration that a single food category was targeted when other food categories bear a significant proportion of the responsibility for childhood obesity. They expressed the view that multiple nutrients or calories across many food and drink sectors should be targeted by regulation if the government is serious about reducing childhood obesity, particularly as substitution to other non-regulated food categories could negate the impact of the SDIL on health *"…why would it be just the soft drink levy, why would you not target cakes and biscuits…that's what we didn't understand at the time." - Food and drink manufacturer..*

There was consensus among participants that it did not make sense for the government to target a category that they considered was already reducing sugar faster than other food categories. Although the SDIL had accelerated the reformulation progress for some, this was

For peer review only - http://bmjopen.bmj.com/site/about/guidelines.xhtml

stated to be already occurring prior to the SDIL announcement. Participants expressed the view that the sector had been unfairly penalised, and that sectors which reformulate should be praised rather than targeted by regulation when other unregulated categories have contributed little towards achieving health goals. *"… the soft drinks category was already well embarked on the journey to reformulation…part of the industry's disappointment and frustration about the announcement of the levy was that they were already absolutely going to deliver what the levy has now kind of made them deliver" – Trade association*

Distrust of government's motivations to introduce the SDIL

Participants stated that the SDIL was politically motivated, not an evidence-based policy. Government policies targeting obesity were described as contradictory and not aligned with one another, particularly the proposed ban on advertising of less healthy foods on TV and online [36]. According to participants, the advertising ban does not distinguish between reformulated and non-reformulated products, and acts as a disincentive to spending on reformulation if they cannot recoup their investment through advertising new products. *"So if you can take something from 40g of sugar to 20g of sugar but you'd only advertise on TV is*

For peer review only - http://bmjopen.bmj.com/site/about/guidelines.xhtml

it's 5(g), then why bother, right, and it also means that they can't tell the world, look at this amazing thing we've done, we've reformulated this" – Advertising consultant.

Perceived disconnectedness between policies led to distrust in the government and a belief that government obesity policy is poorly planned. Distrust was compounded by some companies appearing to be successful at lobbying the government following the announcement, resulting in changes to the regulations as a result of this lobbying, rather than on the basis of health or nutrition, in particular the decision to exclude milk-based drinks. Participants stated this was motivated by some companies being able to gain a competitive advantage, as some milk-based drinks have higher sugar content than soft drinks. Participants also referred to the SDIL as a political tool to distract from other things in the budget in which it was announced *"… I think this was a decision taken within the Treasury by quite a small group of people and it was announced during a Budget by a Chancellor who was trying to distract from some other economic figures that he maybe wasn't too pleased about." – Drink manufacturer*. The fact that the proposal to establish the SDIL had been kept secret, and the announcement was a shock to many, led to this view. *"I think the timing was a*

For peer review only

surprise... Yeah and the way it was done without any form of consultation or pre-announcement." – Drink manufacturer.

Theme 4: Consumer is king

Consumer response to product changes resulting from the SDIL

Industry participants discussed throughout all previous themes that meeting the wishes of consumers was the priority when responding to the SDIL. Taste preferences and tolerance of reformulation changes were critical and companies expressed concerns that consumers might dislike reformulated products if they changed dramatically in a short time period *"… obviously what's critical from our perspective is developing a product that consumers still like the taste of whilst reducing their sugar intake so that we were trying to marry-up those two things." – Drink manufacturer.* Company responses to the SDIL, as well as health and environmental issues more broadly, were vital to maintaining brand loyalty and company reputation in the eyes of consumers. The media were seen as influential in shaping consumer preferences and company reputation, as some newspapers had used graphics to show the sugar content of drinks and this was considered to have influenced purchasing patterns.

For peer review only - http://bmjopen.bmj.com/site/about/guidelines.xhtml

According to informants a small group of very loyal consumers can cause a backlash publicly, which can be picked up by both the news media and social media.

Consumer momentum towards healthier products

Participants stated consumer purchasing patterns are changing, with consumers increasingly choosing lower sugar products, which may also have driven reformulation prior to the SDIL. The policy acted as a catalyst for increasing consumer demand for sugar reduction and some respondents also highlighted the role of social media in driving these trends. Consumers were also reported by participants as “moving away from" artificial ingredients, which leds to challenges in reformulation using non-nutritive sweeteners *"A lot of our consumers like ..., , they don’t want to have sweeteners, they don’t want to have preservatives" – Drink manufacturer*. Some participants suggested that consumers were not lost when sugar was reduced in their favourite products, due to consumer preferences moving towards prioritising health. It was important to participants and their organisations that consumers have enough choice and there were concerns that regulation could limit choice from some.

Theme 5: The future of the SDIL

For peer review only - http://bmjopen.bmj.com/site/about/guidelines.xhtml

Participants discussed the potential of expanding the SDIL to fruit and milk-based drinks, the wider threat to other products, reformulation in other categories, changes in other sectors as a result of the SDIL and the possibility of its reversal by government.

Extending to milk and fruit-based drinks

Concerns were expressed over the Chancellor's proposal to extend the SDIL to milk-based and fruit-based drinks at the time of the announcement *"I don't think politicians think it's done. Obviously we've got the review next year on whether milk-based drinks should be included, and then I think it's 2021 when they'll review the levels as well." – Drink manufacturer*. Participants stated the nutritional benefits of these meant that natural sugars (fructose and lactose) should not be subject to the same regulation as soft drinks. The vitamin and mineral content of these drinks was also discussed as a benefit to children who may not be consuming sufficient fruit, vegetables or calcium from other sources *"… Now you have products that are being developed with high levels of sugar in them so that really does need to be addressed but you don't want to go down the route of demonising milk because it is still a great source of nutrition." – Out-of-home food and drink manufacturer*. Reformulation of

For peer review only

these drinks was considered particularly challenging, as naturally occurring sugars cannot be removed in the same way as added sugars in soft drinks.

Impact on the wider food and drink industry and in other sectors

A wider threat to other products, particularly those included in the PHE Sugar Reduction Strategy [37] (another element of the Childhood Obesity Plan that encouraged voluntary industry reformulation) was discussed. The SDIL demonstrated that the government was willing to implement policy to regulate the food industry in a way that has not been done before. Food and drink companies discussed their companies' attempts to reformulate products not included in the SDIL. The SDIL was described as a rallying call for industry to improve the healthfulness of products. It was also perceived to cause a ripple effect not just regarding health but also sustainability, environment, media and promotions. *"Yeah, I think there is a ripple effect. So, I think it can be both positive and negative. I think in terms of positive, I think it can force companies to reformulate and be more innovative in driving the use of other ways of sweetening products" - Food and drink manufacturer*

Proposal to reverse the SDIL

For peer review only - http://bmjopen.bmj.com/site/about/guidelines.xhtml

Comments made by Boris Johnson in his leadership campaign to become prime minister (July 2019), suggested he might consider repealing the SDIL [38]. These were not taken well by some participants; who indicated that companies had invested heavily in implementing the levy *"I suppose it does feel like a backtrack [reversing the SDIL]. Like we've made all this work and it was at the time quite painful in the sense of it was such a massive change through the supply chain so there was so many things to think about" – Out-of-home food and drink manufacturer*. However, some participants suggested that reversing the SDIL would be well tolerated. *"I think, yeah, the industry would be happy to see the back of it because it's just cumbersome, it's just something, it's just another thing to administer." - Food and drink manufacturer.*

Discussion

Summary

Senior industry perspectives on the SDIL are described in five main themes. Theme 1: *A level playing field…for some*, Theme 2: *Complex to implement, but no lasting negatives*, Theme 3: *Why us? – the SDIL unfairly targets the drinks industry,* Theme 4: *The consumer is king*, and Theme 5: *The future of the SDIL.* The SDIL appeared to create a level playing field

For peer review only - http://bmjopen.bmj.com/site/about/guidelines.xhtml

which industry accepted, however, this was perceived as inadequate due to the exclusion of milk-based drinks and targeting only SSBs, giving some a competitive advantage. Implementation of the SDIL was time consuming and complex, leading to high financial investment to prepare for it. Strategic response to the SDIL was dependent on leadership buy-in and particularly governed by potential consumer responses to product changes associated with the policy. The announcement and subsequent implementation of the SDIL caused a ripple effect beyond the soft drinks industry. The wider food and drink industry perceived it as evidence of the government being willing to regulate to help achieve health goals.

Strengths and limitations

The use of elite interviewing techniques to build relationships with and solicit meaningful responses from participants is a strength of this work. These techniques allowed us to obtain the views of senior professionals from commercial organisations who have often been difficult to recruit to other studies [39]. As evident from the challenges described in the out-of-home sector and supermarkets, including respondents outside of manufacturing allowed wider exploration of the systemic impacts of the SDIL. A limitation of this work, however, is that interviews were carried out over a long period of time due to challenges in recruitment.

For peer review only

Therefore, not all participants experienced the same political context, such as Boris Johnson's threats to reverse the SDIL in July 2019. Initial plans were for longitudinal data collection repeated across the time period of the study. Had all participants been interviewed closer to the implementation of the SDIL in 2018, then repeated in 2020, perspectives on the political events occurring would have been captured from all participants. Unfortunately, challenges to recruitment and access to elite participants led to the abandonment of this plan. Although researcher neutrality was expressed to participants the position of interviewers as public health academics could have led to these recruitment challenges.

The positionality of the researchers may also have led to censoring of responses by some participants. Whilst we sought to descriptively represent industry perspectives, as well as acknowledge our own biases that are typically pro-health policy, it is important to acknowledge that the food and drink industry will have their own biases against health policy that is detrimental to their business survival, as evidenced in previous work [40,41]. Although it was not the aim of the work to explore participant responses in relation to the commercial determinants of health, it is possible that participant responses did not represent the reality of what occurred behind the scenes in the food and drink industry in relation to the SDIL.

For peer review only - http://bmjopen.bmj.com/site/about/guidelines.xhtml

Overlap between some of the responses provided in this work and the ‘typical’ responses explored by other researchers as an industry ‘playbook’ [42] may support this assertion.

Interpretation and implications

Interviewees reported that the technical aspects of drink production, particularly in the out-of-home sector, were not adequately accounted for in the design of the SDIL. An unintended consequence of the milk-based drink exclusion, led to some organisations having to interpret the particulars of the SDIL whilst their queries to HMRC went unanswered. Experiences of participants in this work align with findings that UK Government policy is set up poorly for the purposes of adequate monitoring and evaluation [43]. Future policy should engage with the wider food and beverage sector once a policy is certain to be implemented, to design and communicate technicalities in ways that avoid industry having to interpret themselves what is required and provide timely responses to queries surrounding implementation. Further, respondents indicated that lobbying against the inclusion of milk-based sugar sweetened beverages in the SDIL resulted in this exclusion. Alongside policy engagement in the technicalities of production, an avenue for future research would be to understand in more

For peer review only - http://bmjopen.bmj.com/site/about/guidelines.xhtml

detail the policy process surrounding the SDIL, particularly the influence of the food and drink industry on the policy particulars.

Reviewing their product portfolio was also discussed, where assessments of the product mix as a whole and by individual product were conducted when determining the response to the SDIL. This aligns with previous findings that soft drink companies monitor their internal and external contexts to determine their products' market position in response to a stimulus such as the SDIL, and then respond with marketing or non-marketing activity to influence the purchasing of soft drinks [44]. A crucial external contextual component to response in our findings appears to be consumer response and preferences towards each product, as well as health as a whole.

The UK soft drinks industry was reformulating products to lower sugar alternatives several years before the SDIL was introduced [44,45]. Perspectives expressed by participants align with this and suggest that there is a shift towards healthier drinks as the primary offer for consumers, with the SDIL accelerating the pace of this change. Consumer preferences for healthier products, and our finding that industry prioritises these health preferences in their

For peer review only - http://bmjopen.bmj.com/site/about/guidelines.xhtml

decision making, are likely to have triggered the soft drinks industry to reformulate products prior to the announcement of the SDIL. The advocacy (e.g. Jamie Oliver and Action on Sugar) in the early 2010s [46–48]and government threats to regulate industry [49] may have also increased consumer awareness about the health impact of sugar consumption and had a 'signalling effect' to consumers to reduce their sugar consumption [50]. Participants in our study suggested that the SDIL was adopted by the Government because of the existing popularity of sugar reduction among the public. It is likely that the UK public was aware that SSBs harm health much earlier than the policy announcement, resulting from media activity, such as that related to Jamie Oliver's campaigning [46] PHE's [47]and WHO's reports on sugar [48]. Therefore, the importance of public momentum towards health could be regarded as a trigger for industry action independently from encouraging government action via policy.

Finally, participants expressed concern that policies introduced to combat obesity and other societal issues should be complementary not contradictory. The proposed ban on TV and online advertising of high fat, salt and sugar (HFSS) products by the UK Government [36] was viewed by industry to be misguided as they stated it may stop them being able to advertise their reformulated products; not just those impacted by the SDIL but products

For peer review only

voluntarily reformulated which would still be classified as HFSS. Stakeholder requests for consistency across policy areas was also expressed by interviewees regarding this advertising ban [51]. This indicates that a more consistent approach to determining which products government wants industry to change would help ensure policies do not undermine one another and build trust in government amongst industry.

Conclusion

This study explored food and drink industry perspectives on the SDIL. We found that industry accepted that legislation was useful in levelling the commercially competitive playing field. However, in practice participants stated that the SDIL had not created a 'true' level playing field as little consideration had been given to excluded product categories during policy design. Technical aspects of implementation were not adequately included and led to complexity for out of home retailers. Legislation on SSBs needs to take account of all industry sectors it affects, including out of home retail, as well as the manufacturing sector. Participants stated that only targeting sugary soft drinks was unfair due to the progress already made in the category compared to others (e.g., confectionary). The critical role of consumers in creating momentum towards sugar reduction in SSBs prior to the SDIL

For peer review only - http://bmjopen.bmj.com/site/about/guidelines.xhtml

announcement, as well as dictating response to the SDIL was discussed. It is hypothesised that pro-health public views could be a useful lever in encouraging positive industry action independently of food and drink regulation. The impact of the SDIL was felt beyond the soft drinks industry, driving other product sectors to reformulate in anticipation of future regulation.

Acknowledgements: For the purpose of Open Access, the author has applied a Creative Commons Attribution (CC BY) licence to any Author Accepted Manuscript version arising.

Competing interests: None.

Contributors: Dr. Catrin P Jones: Conceptualization, Methodology, Formal analysis, Investigation, Data Curation, Writing - Original Draft, Project administration. Dr. Hannah Forde: Formal analysis, Writing - Review & Editing. Dr Tarra L. Penney: Conceptualization, Methodology, Investigation, Data Curation, Writing - Review & Editing, Funding acquisition. Dr. Dolly R. Z. Theis: Formal analysis, Writing - Review & Editing

For peer review only

Prof. Steven Cummins: Conceptualization, Methodology, Formal analysis, Writing - Review & Editing, Supervision, Funding acquisition. Prof. Jean Adams: Formal analysis, Writing - Review & Editing, Funding acquisition. Dr. Cherry Law: Formal analysis, Writing - Review & Editing. Prof. Harry Rutter: Formal analysis, Writing - Review & Editing, Funding acquisition. Prof. Richard Smith: Formal analysis, Writing - Review & Editing, Funding acquisition. Prof. Martin White: Conceptualization, Methodology, Resources, Supervision, Project administration, Funding acquisition, Formal analysis, Writing - Review & Editing.

Funding: This project was funded by the NIHR Public Health Research programme (Grant Nos. 16/49/01 and 16/130/01). At the time this study was conducted CPJ, MW, ELR, HF, TLP, DT, JA, OA, SA, were also supported in part by: Programme grants to the MRC Epidemiology Unit from the Medical Research Council (grant No. MC_UU_12015/6 and MC_UU_00006/7); and the Centre for Diet and Activity Research (CEDAR), a UKCRC Public Health Research Centre of Excellence – funding from the British Heart Foundation, Cancer Research UK, the Economic and Social Research Council, the Medical Research Council, the National Institute for Health Research, and the Wellcome Trust, under the auspices of the UK Clinical Research Collaboration is gratefully acknowledged. HF received

For peer review only

funding for her PhD studentship from the Economic and Social Research Council and Public Health England, and she has received further discretionary funding from the Economic and Social Research Council and Murray Edwards College, Cambridge. The views expressed are those of the authors and not necessarily those of the any of the above-named funders. The funders had no role in study design, data collection and analysis, decision to publish, or preparation of the manuscript.

Data availability statement: To maintain participant anonymity and confidentiality, data is not available due to the identifiable nature of participants from transcripts and recordings.

Ethics approval statement: The study was approved by the Humanities and Social Science Ethics Committee at the University of Cambridge, UK (no approval ID was provided).

References

1 Afshin A, Sur PJ, Fay KA, *et al.* Health effects of dietary risks in 195 countries, 1990–2017: a systematic analysis for the Global Burden of Disease Study 2017. *The Lancet* 2019;**393**:1958–72. doi:10.1016/S0140-6736(19)30041-8

2 World Health Organization. Taxes on sugary drinks: Why do it? World Health Organization 2017.

For peer review only - http://bmjopen.bmj.com/site/about/guidelines.xhtml

3 Andreyeva T, Marple K, Marinello S, *et al.* Outcomes following taxation of sugar-sweetened beverages: a systematic review and meta-analysis. *JAMA Netw Open* 2022;**5**:e2215276–e2215276.

4 Teng AM, Jones AC, Mizdrak A, *et al.* Impact of sugar-sweetened beverage taxes on purchases and dietary intake: systematic review and meta-analysis. *Obesity Reviews* 2019;**20**:1187–204.

5 Cawley J, Frisvold D. Taxes on sugar-sweetened beverages: Political economy, and effects on prices, purchases, and consumption. *Food Policy* 2023;**117**:102441.

6 The World Bank. Global SSB Tax Database. https://ssbtax.worldbank.org/ (accessed 25 May 2023).

7 HM Revenue & Customs. Soft Drinks Industry Levy. 2016.https://www.gov.uk/government/publications/soft-drinks-industry-levy/soft-drinks-industry-levy#:~:text=from the levy.-,Policy objective,to reduce the sugar content (accessed 16 Jun 2020).

8 HM Revenue and Customs. Soft Drinks Industry Levy statistics background and references. 2022.https://www.gov.uk/government/statistics/soft-drinks-industry-levy-statistics/soft-drinks-industry-levy-statistics-background-and-references (accessed 10 Nov 2022).

9 Cabinet Office, Department of Health and Social Care, HM Treasury, *et al.* Childhood obesity: a plan for action. 2016.https://www.gov.uk/government/publications/childhood-obesity-a-plan-for-action (accessed 8 Apr 2021).

10 Department of Health and Social Care. Childhood obesity: a plan for action, chapter 2. 2018.

11 HM Revenue & Customs. Guidance: Check if you need to register for the Soft Drinks Industry Levy. 2018.https://www.gov.uk/guidance/check-if-you-need-to-register-for-the-soft-drinks-industry-levy (accessed 21 May 2023).

12 Gauke D. Budget 2016. 2016. https://www.gov.uk/government/publications/budget-2016-documents

13 HM Treasury, Osborne. G. Budget 2016: George Osborne’s speech. 2016.https://www.gov.uk/government/speeches/budget-2016-george-osbornes-speech (accessed 6 Apr 2023).

14 Rogers NT, Cummins S, Forde H, *et al.* Associations between trajectories of obesity prevalence in English primary school children and the UK soft drink industry levy: an

For peer review only

interrupted time series analysis of surveillance data. *medRxiv* Published Online First: 2022. doi:https://doi.org/10.1101/2022.09.16.22280030

15 Hilton S, Buckton CH, Patterson C, *et al.* Following in the footsteps of tobacco and alcohol? Stakeholder discourse in UK newspaper coverage of the Soft Drinks Industry Levy. *Public Health Nutr* 2019;**22**:2317–28. doi:10.1017/S1368980019000739

16 Economics O. The Economic Impact of the Soft Drinks Levy, Final Report. *London: Oxford Economics* 2016.

17 Penney TL, Adams J, White M. Industry reactions to the UK Soft Drinks Industry Levy: Unpacking the evolving discourse from announcement to implementation. *J Epidemiol Community Health (1978)* 2018;**72**:A43. doi:10.1136/jech-2018-SSMabstracts.88

18 Law C, Cornelsen L, Adams J, *et al.* An analysis of the stock market reaction to the announcements of the UK Soft Drinks Industry Levy. *Econ Hum Biol* 2020;**38**:100834. doi:10.1016/j.ehb.2019.100834

19 Law C, Cornelsen L, Adams J, *et al.* The impact of UK Soft Drinks Industry Levy on manufacturers' domestic turnover. *Econ Hum Biol* 2020;:100866.

20 Penney T, Adams J, White M. LB4 Industry reactions to the UK soft drinks industry levy: unpacking the evolving discourse from announcement to implementation. BMJ 2018. A43.1-A43. doi:10.1136/jech-2018-ssmabstracts.88

21 Hilton S, Buckton CH, Katikireddi SV, *et al.* Who says what about sugar-sweetened beverage tax? Stakeholders' framing of evidence: a newspaper analysis. *The Lancet* 2017;**390**:S44. doi:10.1016/s0140-6736(17)32979-3

22 Buckton CH, Hilton S, Patterson C, *et al.* DID PROPONENTS AND OPPONENTS OF THE SOFT DRINKS INDUSTRY LEVY USE THE NEWS MEDIA TO INFLUENCE THE POLICY DEBATE? A QUALITATIVE DISCOURSE ANALYSIS USING PRACTICAL REASONING. *J Epidemiol Community Health (1978)* 2018;**72**:A1–2. doi:10.1136/jech-2018-SSMabstracts.2

23 Buckton CH, Patterson C, Hyseni L, *et al.* The palatability of sugar-sweetened beverage taxation: A content analysis of newspaper coverage of the UK sugar debate. *PLoS One* 2018;**13**:1–15. doi:10.1371/journal.pone.0207576

24 Forde H, Penney TL, White M, *et al.* Understanding Marketing Responses to a Tax on Sugary Drinks: A Qualitative Interview Study in the United Kingdom, 2019. *Int J Health Policy Manag* Published Online First: 23 February 2022. doi:10.34172/ijhpm.2022.5465

For peer review only

25 Braun V, Clarke V. *Successful Qualitative Research: A Practical Guide for Beginners*. SAGE 2013.

26 White M. Evaluation of the health impacts of the UK Treasury Soft Drinks Industry Levy (SDIL). 2017;:1–46.https://www.journalslibrary.nihr.ac.uk/programmes/phr/1613001/#/

27 Harvey WS. Methodological Approaches for Interviewing Elites. *Geogr Compass* 2010;**4**:193–205. doi:10.1111/j.1749-8198.2009.00313.x

28 Berry JM, Browne W. Validity and Reliability Issues In Elite Interviewing. *PS Polit Sci Polit* 2003;**35**:679–82.

29 Harvey WS. Strategies for conducting elite interviews. *Qualitative Research* 2011;**11**:431–41. doi:10.1177/1468794111404329

30 Goldstein K. Getting in the Door: Sampling and Completing Elite Interviews. *Political Science & Politics* 2002;**35**:669–72. doi:10.1017/S1049096502001130

31 Stephens N. Collecting data from elites and ultra elites: Telephone and face-to-face interviews with macroeconomists. *Qualitative Research* 2007;**7**:203–16. doi:10.1177/1468794107076020

32 Braun V, Clarke V. Using thematic analysis in psychology. *Qual Res Psychol* 2006;**3**:77–101.

33 Braun V, Clarke V. Can I use TA? Should I use TA? Should I not use TA? Comparing reflexive thematic analysis and other pattern-based qualitative analytic approaches. *Couns Psychother Res* 2020;:37–47. doi:10.1002/capr.12360

34 Braun V, Clarke V. What can 'thematic analysis' offer health and wellbeing researchers? *Int J Qual Stud Health Well-being* 2014;**9**. doi:10.3402/qhw.v9.26152

35 Department of Health and Social Care. Calorie labelling in the out of home sector: implementation guidance. 2021.https://www.gov.uk/government/publications/calorie-labelling-in-the-out-of-home-sector/calorie-labelling-in-the-out-of-home-sector-implementation-guidance#:~:text=The%20out%20of%20home%20sector%20is%20generally%20considered%20to%20be,on%20or%20off%20the%20premises (accessed 14 Dec 2022).

36 Department for Digital Culture Media & Sport, Department of Health & Social Care. Introducing a total online advertising restriction for products high in fat, sugar and salt (HFSS). 2021.https://www.gov.uk/government/consultations/total-restriction-of-online-advertising-for-products-high-in-fat-sugar-and-salt-hfss/introducing-a-total-

For peer review only

online-advertising-restriction-for-products-high-in-fat-sugar-and-salt-hfss (accessed 6 Jan 2023).

37 Office for Health Improvement and Disparities. Sugar, salt and calorie reduction and reformulation. 2017.https://www.gov.uk/government/collections/sugar-reduction (accessed 11 Jan 2023).

38 Stewart H. 'Sin taxes': Boris Johnson vows to review sugar levy. The Guardian. 2019.https://www.theguardian.com/politics/2019/jul/03/boris-johnson-vows-to-review-whether-sugar-tax-improves-health (accessed 19 Oct 2022).

39 Forde H, Penney T, White M, *et al.* A Framework for Understanding Marketing Responses to a Tax on Sugary Drinks: a Qualitative Interview Study in the United Kingdom, 2019. *Int J Health Policy Manag* 2022.

40 Lacy-Nichols J, Marten R, Crosbie E, *et al.* The public health playbook: ideas for challenging the corporate playbook. *Lancet Glob Health* 2022.

41 Wiist WH. The corporate play book, health, and democracy: the snack food and beverage industry's tactics in context. *Sick societies: Responding to the global challenge of chronic disease* 2011;:204–16.

42 Petticrew M, Katikireddi SV, Knai C, *et al.* 'Nothing can be done until everything is done': The use of complexity arguments by food, beverage, alcohol and gambling industries. *J Epidemiol Community Health (1978)* 2017;**71**:1078–83. doi:10.1136/jech-2017-209710

43 Theis D, White M. Is Obesity Policy in England Fit for Purpose? Analysis of Government Strategies and Policies, 1992–2020. *Milbank Quarterly* 2021;**99**:126–70. doi:10.1111/1468-0009.12498

44 Forde H, Penney T, White M, *et al.* Understanding Marketing Responses to a Tax on Sugary Drinks: a Qualitative Interview Study in the United Kingdom, 2019. *Int J Health Policy Manag* 2022.

45 Scarborough P, Adhikari V, Harrington RA, *et al.* Impact of the announcement and implementation of the UK Soft Drinks Industry Levy on sugar content, price, product size and number of available soft drinks in the UK, 2015-19: A controlled interrupted time series analysis. *PLoS Med* 2020;**17**:e1003025.

46 Boseley S. Jamie Oliver's Sugar Rush: a crusade to save Britain's health. The Guardian. 2015.

For peer review only

47 Tedstone A, Targett V, Allen R. Sugar Reduction: The evidence for action About Public Health England. *Public Health England* Published Online First: 2015.www.gov.uk/phe%5Cnwww.facebook.com/PublicHealthEngland

48 World Health Organization. Sugars intake for adults and children. 2015. https://www.who.int/publications/i/item/9789241549028 (accessed 14 Oct 2022).

49 Nieburg O. Kellogg shuns 'silver bullet' 30% UK cereals sugar cap. bakeryandsnacks.com. 2013.https://www.bakeryandsnacks.com/Article/2013/01/07/Kellogg-shuns-30-sugar-cap-proposal-for-UK-cereals (accessed 6 Jan 2023).

50 Rose D, Theis Z. What influences government policymaking? The case of childhood obesity in England. 2022.

51 Forde H, Boyland EJ, Scarborough P, *et al.* Exploring the potential impact of the proposed UK TV and online food advertising regulations: a concept mapping study. *BMJ Open* 2022;**12**. doi:10.1136/bmjopen-2021-060302

For peer review only

Supplementary File 1: Researcher Reflexivity

Braun and Clarke discuss reflexivity as a fundamental characteristic of thematic analysis, involving critical reflection of researcher perspectives, and how these will be integrated within the analysis and interpretation of data [22]. The complete elimination of bias is not something that can be achieved in qualitative research and more importantly should not be an aim. Unlike statistical analysis, the researcher is the tool of analysis. The researcher therefore is an integral part of the analytic process and to conduct qualitative thematic analysis well, they must develop an understanding or how their own perspectives, position and view of reality helps illuminate will influence the analysis [22].

This study aimed to centre participants' words in a descriptive manner to preserve their intention. This approach was also chosen due to reflection by the research team on our positionality as public health academics. We aimed to understand industry perspectives regarding the SDIL; however, it is important to acknowledge that the personal and professional goals of the research team (authors on this paper) as public health researchers are likely to be different from those of people working in the food and drink industry. Therefore, a descriptive approach was selected which prizes participants' words and perspectives over and above researcher interpretations. Whilst our perspectives have still influenced the analysis, as they should in good qualitative practice, we sought to minimise the influence of our biases and negativity towards some of the practices of the food and drink industry, to truly 'listen' to the perspectives of our participants. As a result, a modified version of Braun and Clarke's thematic analysis was used; reflexivity was a priority throughout the analysis in line with the approach however we sought to be less interpretive than their more recent guidance proposes [22].

For peer review only

It is also important to note that, although we have taken a descriptive approach, the results represent participant perspectives. Whilst the researchers work to put aside their biases which may lean towards those more critical of the food and drink industry; statements, findings and themes found do not represent an objective truth, rather the reported perspectives of participants. We urge readers of this work to use their own critical reflection when interpreting and using these findings.

For peer review only

COREQ (COnsolidated criteria for REporting Qualitative research) Checklist

A checklist of items that should be included in reports of qualitative research. You must report the page number in your manuscript where you consider each of the items listed in this checklist. If you have not included this information, either revise your manuscript accordingly before submitting or note N/A.

Topic	Item No.	Guide Questions/Description	Reported on Page No.
**Domain 1: Research team and reflexivity**			
Personal characteristics			
Interviewer/facilitator	1	Which author/s conducted the interview or focus group?	
Credentials	2	What were the researcher's credentials? E.g. PhD, MD	
Occupation	3	What was their occupation at the time of the study?	
Gender	4	Was the researcher male or female?	
Experience and training	5	What experience or training did the researcher have?	
Relationship with participants			
Relationship established	6	Was a relationship established prior to study commencement?	
Participant knowledge of the interviewer	7	What did the participants know about the researcher? e.g. personal goals, reasons for doing the research	
Interviewer characteristics	8	What characteristics were reported about the inter viewer/facilitator? e.g. Bias, assumptions, reasons and interests in the research topic	
**Domain 2: Study design**			
Theoretical framework			
Methodological orientation and Theory	9	What methodological orientation was stated to underpin the study? e.g. grounded theory, discourse analysis, ethnography, phenomenology, content analysis	
Participant selection			
Sampling	10	How were participants selected? e.g. purposive, convenience, consecutive, snowball	
Method of approach	11	How were participants approached? e.g. face-to-face, telephone, mail, email	
Sample size	12	How many participants were in the study?	
Non-participation	13	How many people refused to participate or dropped out? Reasons?	
Setting			
Setting of data collection	14	Where was the data collected? e.g. home, clinic, workplace	
Presence of non-participants	15	Was anyone else present besides the participants and researchers?	
Description of sample	16	What are the important characteristics of the sample? e.g. demographic data, date	
Data collection			
Interview guide	17	Were questions, prompts, guides provided by the authors? Was it pilot tested?	
Repeat interviews	18	Were repeat inter views carried out? If yes, how many?	
Audio/visual recording	19	Did the research use audio or visual recording to collect the data?	
Field notes	20	Were field notes made during and/or after the inter view or focus group?	
Duration	21	What was the duration of the inter views or focus group?	
Data saturation	22	Was data saturation discussed?	
Transcripts returned	23	Were transcripts returned to participants for comment and/or	

For peer review only

Topic	Item No.	Guide Questions/Description	Reported on Page No.
		correction?	
**Domain 3: analysis and findings**			
Data analysis			
Number of data coders	24	How many data coders coded the data?	
Description of the coding tree	25	Did authors provide a description of the coding tree?	
Derivation of themes	26	Were themes identified in advance or derived from the data?	
Software	27	What software, if applicable, was used to manage the data?	
Participant checking	28	Did participants provide feedback on the findings?	
Reporting			
Quotations presented	29	Were participant quotations presented to illustrate the themes/findings? Was each quotation identified? e.g. participant number	
Data and findings consistent	30	Was there consistency between the data presented and the findings?	
Clarity of major themes	31	Were major themes clearly presented in the findings?	
Clarity of minor themes	32	Is there a description of diverse cases or discussion of minor themes?	

Developed from: Tong A, Sainsbury P, Craig J. Consolidated criteria for reporting qualitative research (COREQ): a 32-item checklist for interviews and focus groups. *International Journal for Quality in Health Care*. 2007. Volume 19, Number 6: pp. 349 – 357

Once you have completed this checklist, please save a copy and upload it as part of your submission. DO NOT include this checklist as part of the main manuscript document. It must be uploaded as a separate file.

For peer review only
